# Abstracts: PICS-AICS Virtual Symposium September 10–12, 2020

**DOI:** 10.1007/s00246-020-02408-w

**Published:** 2020-07-31

## 1

### Surgical Treatment of Infants with Critical Aortic Stenosis

Elnur Imanov^1^, Surkhay Musayev^1^, Farida Hajyeva^1^, Samir Allahverdiyev^1^, Sabina Hasanova^1^, Leman Rüstemzade^1^, Fuad Abdullayev^2^, Vasiliy Lazoryshynets^3^, Oleksandr Pliska^4^

^1^Health Ministry of Republic of Azerbaijan Education Therapeutic Clinic of Azerbaijan Medical University, Baku, Azerbaijan. ^2^4Health Ministry of Republic of Azerbaijan Scientific Center of Surgery Named After M.A. Topchubashov Pediatric Cardiac Surgery and Neonatal Surgery center, Baku, Azerbaijan. ^3^Amosov National Institute of Cardiovascular Surgery, Kiev, Ukraine. ^4^Dragomanov National Pedagogical University, Kiev, Ukraine

**Abstract**

Critical aortic stenosis is a complex pathology in newborns requiring emergency care. The treatment of such patients is a complex and complicated problem. Treatment methods include balloon valvuloplasty and open surgical valvuloplasty. The aim of the study was to analyze our own experience in surgical treatment and balloon valvuloplasty of critical aortic stenosis in newborns.

From 2006 to 2019, the State Institution “National Institute of Cardiovascular Surgery named after N. M. Amosova of the NAMS of Ukraine” and Ministry of Health of Azerbaijan, Scientific-Research Surgical Institute named after M.A. Topchubashov Pediatric Cardiac Surgery Center treated 58 infants with aortic stenosis. At the same time, 47 (81%) patients (group I) underwent X-ray endovascular balloon valvuloplasty of aortic stenosis, and 11 (19%) patients (group II) underwent surgical treatment.

After balloon valvuloplasty, a significant decrease in the gradient on the aortic valve was observed in group I patients and an increase in the ejection fraction of the left ventricle. However, in a mid-term observation, the pressure gradient on the aortic valve in most patients increased, and aortic insufficiency began to increase. After surgical correction, good immediate and mid-range results were noted with respect to the gradient on the aortic valve and the degree of insufficiency.

Both surgical valvuloplasty and balloon valvuloplasty are effective treatments for aortic stenosis in newborns with good immediate results. The study indicates that balloon valvuloplasty is an acceptable alternative to surgical treatment in patients with signs of severe heart failure, but tends to increase aortic insufficiency in the long term. The length of stay of patients with balloon valvuloplasty is twice shorter for such surgical valvuloplasty, which significantly reduces the economic costs of treatment.

## 2

### Immediate vs. Staged Multivessel PCI Strategy for Patients with STEMI and Multivessel Disease: A Systematic Review and Meta-analysis

Hu Mengjin, Yang Yuejin

Fuwai Hospital, National Center for Cardiovascular Diseases, Chinese Academy of Medical Sciences and Peking Union Medical College, Beijing, China, Beijing, China

**Abstract**

**Background**: The recent guidelines and randomized trials favor multivessel percutaneous coronary intervention strategy (MV-PCI) for patients with ST-segment elevation myocardial infarction (STEMI) and multivessel disease (MVD). However, the optimal strategy of MV-PCI remains unknown.

**Methods and Results**: We conducted PUBMED, EMBASE, Web of Science, the Cochrane database (CENTRAL), clinicaltrial.gov, and Google Scholar for studies comparing immediate versus staged MV-PCI in patients with STEMI and MVD. Primary endpoints were short-term (in-hospital or 30 days) major adverse cardiovascular events (MACE), all-cause mortality, cardiac death, myocardial infarction, and revascularization. Secondary endpoints included long-term (6 months or more, longest follow-up) MACE, all-cause mortality, cardiac death, myocardial infarction, and revascularization. 18 (4 randomized) trials with 8100 patients fulfilled the inclusion criteria, of whom 3646 (45.0%) received immediate MV-PCI and 4454(55.0%) received staged MV-PCI. Relative to staged MV-PCI, immediate MV-PCI was associated with higher short-term MACE (odds ratio [OR], 1.99; 95% confidence interval [CI], 1.13–3.50; P = 0.02), short- and long-term all-cause mortality (OR, 3.96; 95% CI, 2.07–7.59; P < 0.0001, OR, 2.12; 95% CI, 1.46–3.07; P < 0.0001, respectively), and short-term cardiac death (OR, 4.78; 95% CI, 2.17–10.53; P = 0.0001). There was a non-significant trend toward higher long-term MACE (OR, 1.23; 95% CI, 0.98–1.54; P = 0.07) and cardiac death (OR, 1.75; 95% CI, 0.93–3.30; P = 0.08) with immediate MV-PCI versus staged MV-PCI. Revascularization and myocardial infarction between immediate versus staged MV-PCI strategies were similar.

**Conclusions**: This meta-analysis suggests that among patients with STEMI and MVD, staged MV-PCI may be the optimal revascularization strategy.

## 3

### Atretic Aortic Coarctation: Usefulness of Microcatheter and CTO Wires

Marco Alejandro Solórzano Vázquez, Daniel Eugenio Lopez Ibarra, María Fernanda Corona Reynoso

UMAE T1 IMSS, Leon Gto, Mexico

**Abstract**

This is the case of a 43-year-old male with a history of hypertension in treatment with Telmisartan/HCTZ 80/25 mg OD, Amlodipine 10 mg OD, Metoprolol 100 mg BID.

He was referred to our center as a case of uncontrolled secondary hypertension due to aortic coarctation. Physical examination revealed a systolic murmur II/IV in the left sternal border, in the left infraclavicular area, and under the left scapula. A supine arm–leg blood pressure gradient of 30 mmHg (upper limbs 180/100 mmHg). ECG denotes left ventricular hypertrophy. Echocardiography findings in the suprasternal view showed coarctation of aorta after the left subclavian artery with no flow through aortic lumen.

**TC Angiography:** Reconstruction on axial, sagittal, and coronal slices was analyzed in order to view the relationship between proximal and distal segments of a possible aortic isthmus atresia. A previous attempt to treat this coarctation was performed 6 months earlier without success with bilateral radial thrombosis at the first visit to our center.

**Procedure:** We performed double arterial access through left brachial and right femoral route. Simultaneous antegrade and retrograde aortograms through 6Fr multipurpose Guide catheters gave an idea of the true length of occlusion or the so-called thickness of the separating membrane which varied from 3 to 5 mm. The case was attempted retrogradely with a Turnpike spiral microcatheter (Teleflex, Wayne, PA, USA) and wire escalation technique with a ProVia 6, 9, and 12 guidewire (Medtronic, Minneapolis, Minnesota US). Initially, the wire crossed to a false lumen. Fortunately, the rotational advancement when rotated in a clockwise direction of the mirocatheter allowed us to reshape the guidewire by changing the angle of primary bend and length to find a true lumen and using multiple angiographic views (LAO and PA), in order to finally cross into the true lumen. The true lumen was confirmed by passing a 2.6F microcatheter and a 20 mm EnSnare through brachial route. A 2.0 × 15 mm coronary balloon was used to predilate the lesion, followed by balloon-assisted tracking to cross to the distal aortic arch. The wire changed to 0.035″ system, following balloon dilatation with a 8 × 40 mm balloon. Afterwards we advanced using a 14Fr Mullins sheath (Cook, Bloomington, IN, USA) across the lesion. A 8Zig 39 mm CP stent was mounted over a 18 × 40 mm Maxi LD (Cordis, Hialeah, FL, USA) and was deployed at 6 atm. Final angiography showed good angiographic results and zero pressure gradient with no dissection.

**Results:** The patient was discharged on day 2 from hospital. His blood pressure improved on the next month with a reduction of pharmacological treatment to half dose. A CT angiogram done a month after the procedure showed good result and no dissection.

**Conclusions:** We conclude that aortic atresia in adults can be treated percutaneously using coronary CTO hardware and skills with good success, especially if the team is expert in using chronic total occlusion coronary guidewires and the Cath Lab is equipped with covered stents for possible aortic dissection and rupture.

## 4

### Early Results of PT Valve in Native Right Ventricular Outflow Tract for Patients with Severe Pulmonary Regurgitation

Shu Chen, Xiaoke Shang, Changdong Zhang, Bin Wang, Man Liao, Nianguo Dong

Union Hospital, Tongji Medical College, Huazhong University of Science and Technology, Wuhan, China

**Abstract**

**Background:** Transcatheter pulmonary valve replacement (TPVR) is a new, less invasive alternative to surgical valve replacement. We report the first in man implantation of a novel transcatheter pulmonary valve (TPV) for patients with severe pulmonary regurgitation (PR) in native right ventricular outflow tract (RVOT).

**Method**: Patients with severe residual PR were selected on a case-by-case basis according to the anatomical features of RVOT and design of the valve. The device (Med-Zenith PT Valve) is a porcine pericardial tissue valve mounted on a self-expanding nitinol frame with unique symmetric dumbbell-shape designed. Patient demographics and pre-procedural, intraprocedural, and follow-up data were reviewed.

**Result:** Twenty-two patients (17 Males/5 Females) with severe PR (grade 4 +) were enrolled in this study with mean age of 28.8 ± 10.5 years and weight of 58.6 ± 10.7 kg. All patients had previous surgery for tetralogy of Fallot (TOF)/double ventricular outflow tract (DORV). Thirteen patients were symptomatic with New York Heart Association (NYHA) heart function III/IV and 9 with NYHA II at baseline. The mean diameter of distal main pulmonary artery (MPA), sub-local residual pulmonary leaflet, and RVOT aneurysm measured in Computed tomography angiography (CTA) were 33.6 ± 6.1 mm, 34.7 ± 6.0 mm, and 41.9 ± 9.3 mm. The landing zone lied usually within these three levels. Mean devices size used was 25.3 ± 1.3 mm waist/39.1 ± 4.9 mm flare. Successful valve implantation was achieved in all patients. No device malposition, coronary obstruction, reduced flow to the PA branches, or paravalvular leak were noted during the procedures. Mean pulmonary artery diastolic pressure increased from 5.8 ± 3.1 mmHg to 11.3 ± 2.5 mmHg (P < 0.05). In one month’s follow-up, Magnetic Resonance Imaging (MRI) revealed regression of right ventricle remodeling with right ventricle end-diastolic volume index (RVEDVI) decreased from 182.7 ± 27.5 ml/m^2^ to 121.9 ± 27.5 ml/m^2^ after intervention (P < 0.05). After successful valve implantation, the mean transpulmonary valve pressure gradient measured with echocardiography was 7.1 ± 2.9 mmHg.

**Conclusion:** This first in man study demonstrates the initial safety, efficacy, and feasibility of the Med-Zenith PT Valve in the treatment of severe PR.

**Key words:** Transcatheter pulmonary valve replacement, Med-Zenith PT Valve, Native Right Ventricular Outflow Tract, pulmonary regurgitation, Tetralogy of Fallot

## 5

### Juxtaposition of Atrial Appendages and ASD Device Closure—The Truth in the Lie

Sreekanthan Sundararaghavan, Jonathan Choo Tze Liang, Tenghong Tan

Singhealth, Singapore, Singapore

**Abstract**

Device closure of Secundum ASD has become the standard of care in most countries. Success of the procedure lies in understanding the atrial septal anatomy. Abnormal septal anatomy adds to the complexity of the procedure and could increase the complication rates especially embolization.

We describe the imaging planes in a case of secundum ASD with juxtaposed atrial appendage that was successfully closed using an Amplatzer septal occluder.

An 11-year-old female with a diagnosis of secundum ASD and juxtaposition of the atrial appendage underwent successful deployment of a 18-mm Amplatzer septal occluder with the help of TEE guidance. The atrial septal orientation was clearly ascertained using orthogonal planes and 3D data sets as there is a great potential to misconstrue the mouth of the juxtaposed appendage to be the defect thereby minimizing the risk of embolization.

**Conclusion:** The lie of the atrial septum is altered significantly in the presence of juxtaposition of atrial appendage. Appropriate delineation of the plane of the septum by TEE and 3D data sets is important to get the appropriate angles in order to successfully deploy the septal occluder.

## 6

### Evaluation of Short and Intermediate-Term Follow-Up Results of Percutaneous Closure of Ventricular Septal Defects Using Different Devices: A Single-Center Experience from Mansoura, Egypt

Hala Elmarsafawy, Mona Hafez, Gehan Elsawah, Asmaa Bakr, Shaimaa Rakha

Mansoura University, Mansoura, Egypt

**Abstract**

**Background and Aim:** Ventricular septal defects are the most common congenital cardiac defects (30% of all CHD). Transcatheter closure of VSDs has advanced rapidly, with the introduction of variable device designs. Many studies addressed the safety and immediate results, but follow-up data are still limited. Therefore, this work presents our short and intermediate-term experience of percutaneous VSD closure.

**Methods:** Between September 2012 and September 2019, 68 patients with VSDs underwent transcatheter closure. Patients should have a significant left-to-right shunt through VSD with anatomy suitable for transcatheter closure. All the patients were generally anesthetized with procedures performed under TEE or TTE with fluoroscopic guidance. Patient demographics, defect measurements, procedure details including device types, short-term complications, and follow-up data were collected.

**Results:** The mean age of patients was 9.6 ± 3.7 years, the median bodyweight of the patients was 30.5 (21.25–44.5) kg, 38 (55.9%) were males, and 67(98.5%) cases were successful procedures. The median follow-up duration was 37 months (range 3–83 months). The mean defect diameter was 3.5  ±  1.4 mm. The most common type was perimembranous VSD (33 cases), one of them was associated with PDA which was closed in the same setting, 6 cases had muscular outlet VSD, 17 cases with mid muscular VSD, and 11 cases had residual postsurgical closure. The devices used were as follows 30 (44.1%) pfm Nit-Occlud^®^ Lê VSD Coil, 21 (30.9%) Hyperion™ VSD Muscular Occluders, 5(7.4) Amplatzer muscular occlude, 11(16.2%) Amplatzer Duct Occluder ADO I, and 1 (1.5%) ADO II. One ADO I device embolized in perimembranous VSD, which was retrieved successfully. No significant difference in the success rate between groups. A complete heart block was detected in 2 cases immediately post procedure and resolved with steroid intake. Residual flow after the device insertion was seen immediately in 9 cases, which resolved in 6 cases within 6 months of follow-up. No cardiac erosion was detected in any of the followed cases and no mortality occurred during the intermediate-term follow-up.

**Conclusion:** Percutaneous closure success of VSDs was comparable among different devices. Periprocedural complications were limited with favorable intermediate-term outcome with no significant morbidity and no mortality.

## 7

### Hypoxemia Following Balloon Pulmonary Valvuloplasty for Critical Pulmonary Stenosis

Rajiv Devanagondi, Glenn Leonard

University of Rochester Medical Center, Rochester, USA

**Abstract**

**Background:** Infants with critical pulmonary valve stenosis (PS) require augmented pulmonary blood flow by maintaining ductal patency to establish normal oxygen saturation. Balloon pulmonary valvuloplasty effectively treats critical PS. However, some infants require prolonged supplemental oxygen post catheterization to maintain normal oxygen saturation due to decreased compliance of the hypertrophied right ventricle, and increased right-to-left atrial level shunt. Supplemental oxygen duration post procedure, and factors associated with prolonged oxygen treatment, have not been described previously.

**Methods:** Catheterization, echocardiogram, and progress notes were retrospectively reviewed for all patients following balloon pulmonary valvuloplasty for critical PS between 1/1/2000 and 3/1/2019. Patients with additional congenital heart disease (apart from PFO or small ASD) were excluded. Supplemental oxygen was continued post procedure to maintain resting oxygen saturation > 88% by pulse oximetry, though intermittent, self-resolved desaturations to 80% were not treated with additional oxygen. Data were reported as *n* (%) and median (IQR or range). Continuous variables were compared using Wilcoxon ranked-sum test. Categorical variables were compared using Fisher’s exact test. *p* < 0.05 was considered statistically significant.

**Results:** Twenty-six infants had balloon pulmonary valvuloplasty at age 2 (1–5) days with weight 3.4 (2.8–3.8) kg. Four (15%) were premature. Eleven (42%) needed supplemental oxygen pre-catheterization including 4 (15%) that were intubated. By echocardiogram, baseline right ventricle (RV) to pulmonary artery (PA) peak gradient (PG) was 82 (69–93) mmHg, pulmonary valve diameter 7 (6.5–7.9) mm and Z-score − 1.6 (− 1.9 to − 0.5), and tricuspid valve diameter 12.1 (10.5–13.2) mm and Z-score +0.3 (− 1 to +0.8). Seven (37%) had ≥ mild RV systolic dysfunction, 2 (8%) had mild RV hypoplasia, and 11 (42%) had ≥ mild RV enlargement. The baseline RV-PA PG by catheter was 61 (45–73) mmHg and baseline RV/systemic pressure ratio 1.5 (1.3–1.8). Following valvuloplasty, RV-MPA PG was reduced to 15 (12–20) mmHg and RV/systemic pressure ratio 0.8 (0.6–0.9). Prostaglandin duration post procedure was 1 (0–2.3) days and oxygen supplementation was 1 (range: 0–15) day, with 8 patients receiving ≥ 5 days supplemental oxygen prior to hospital discharge. Two (8%) patients were discharged home with supplemental oxygen. Infants with ≥ 5 days supplemental oxygen post-procedure had smaller tricuspid valve diameter (10.4 vs. 13 mm, *p* = 0.03), and increased baseline RV/systemic pressure ratio (1.7 vs. 1.4, *p* = 0.04). There were no other statistically significant differences between groups and specifically lower gestational age, need for oxygen or intubation pre-valvuloplasty, smaller pulmonary valve diameter, greater post-procedure RV-PA PG, and RV dysfunction were not associated with longer duration of supplemental oxygen post-valvuloplasty. No patient had additional transcatheter or surgical procedures prior to discharge.

**Conclusions:** Balloon pulmonary valvuloplasty effectively treats critical pulmonary stenosis, though the duration of post-procedure oxygen supplementation is highly variable, from 0 to 15 days in this series. Patients with smaller tricuspid valves and increased RV pressure at baseline were more likely to have a prolonged oxygen requirement post procedure.

## 8

### Short-Term Outcomes Of Percutaneous Device Closure of Patent Ductus Arteriosus Using The Amplatzer Duct Occluder Device in Comparison To The Nitoccluder PDA-R Device

Khaled Refaat

Benisuef University, Cairo, Egypt

**Abstract**

Patent Ductus Arteriosus (PDA) is a common form of congenital heart disease. It has been estimated to occur in 1 in 2500–5000 live births. As an isolated lesion, it represents 9–12% of all congenital heart diseases. The benefits of the transcatheter closure of PDA compared to surgical closure seem obvious in terms of shorter in-hospital stay, high success rates, no scar, and insignificant morbidity.

**Objectives:** To investigate the safety, effectiveness, and hemodynamic effects of percutaneous closure of patent ductus arteriosus (PDA) using Amplatzer ductal occluder (ADO) (AGA medical, Golder Valley, MN, USA) in comparison to the Nit-Occluder PDA-R (pfmmedical, Koln, Germany), a novel PDA occluding device.

**Methods:** A prospective randomized study that included 100 patients with diagnosed PDA, patients enrolled in this study were divided into two groups: Group I: 50 patients with PDA closed by Nit-occluder PDA-R device. Group II: 50 patients with PDA closed by the amplatzer duct occluder device. Device performance and immediate and short-term outcomes were assessed.

**Results:** There were significant decrease in left ventricular dimensions in both groups, the procedure time, fluoroscopy time, and residual shunt rates were similar between the two groups. Procedural success rate was 100% in both groups. Although the residual shunt rate was higher in the PDA-R group immediately after the procedure, the difference was not statistically significant (16.0% vs. 12.0%; *p* value 0.762). No deaths occurred in any of the groups, and there were no differences in complication rates during the short- and mid-term follow-up periods.

**Conclusion:** The PDA-R device can be used for PDA closure because of its safety, effectiveness, and simplicity in use. According to our short- and mid-term findings, the results it yields are similar to those of the ADO; thus, it may be the preferred choice owing to its low cost.

**Keywords:** Patent ductus arteriosus; amplatzer duct occluder; transcatheter closure; Novel PDA-R device; percutaneous treatment.

## 9

### Short and Intermediate-Term Safety and Efficacy of Percutaneous Device Closure for Secundum Atrial Septal Defects using Occlutech Figulla^®^ Occluder N

Khaled Refaat

Benisuef University, Cairo, Egypt

**Abstract**

**Objectives:** To investigate the safety, effectiveness, and hemodynamic effects of percutaneous atrial septal defect (ASD) closure using the Occlutech^®^ devices in a prospective trial.

**Background:** Transcatheter closure has become the method of choice for most patients with secundum ASD. Although the Occlutech device may have some advantageous characteristics, there is a paucity of data on outcomes after the use of this relatively new device.

**Methods:** Observational, single-arm study, including 111 patients who underwent ASD closure between October 2013 and December 2015. Device performance, and short and intermediate-term outcomes were assessed.

**Results:** Median age and ASD sizes were 7.8 years (8 months–59 years) and 16.5 mm (4.8–38 mm), respectively. Deficient or absent retro-aortic rim was observed in 30 patients (27%). All patients had dilated right-side chambers. Pulmonary artery systolic pressure > 35 mmHg was observed in 57 (51%) patients who had significantly larger ASDs (*p* = 0.009) and larger RV lengths (*p* = 0.006). Implantation of an Occlutech device (mean size of 19.4 ± 8 mm) with successful closure was reported in 95.5%. Closure success was linked to larger IVC rims (p = 0.009). An IVC rim ≥ 7.2 mm is 97.1% sensitive, while IVC rim ≥ 11.2 mm is 100% specific for closure success. Median follow-up of 6 months was obtained in all patients. Successful closure leads to significant regression of RV and pulmonary artery dimensions at 1, 3, and 6 months of follow-up (*p* < 0.001).

**Conclusions:** Transcatheter closure of secundum ASDs using the Occlutech septal Occluder is safe and effective in children, adolescents, and adults. The device performed well in a wide range of anatomical scenarios resulting in excellent short and intermediate-term outcomes. Sufficient IVC rim is the most important factor in predicting successful closure. An IVC rim ≥ 7.2 mm is 97.1% sensitive, while an IVC rim ≥ 11.2 mm is 100% specific for closure success.

**Keywords:** Atrial septal defect, Transcatheter closure, Pulmonary artery systolic pressure, Right ventricle.

## 10

### Predictors for Dilated Aorta in Repaired and Unrepaired Tetralogy of Fallot

Khaled Refaat

Benisuef University, Cairo, Egypt

**Abstract**

**Abstract**

Aortic root pathology has been described in patients with Tetralogy of Fallot, although the most common reason for repeat surgery in the adult after TOF repair relates to problems in the right ventricular outflow tract, the aortic root is often forgotten.

**Objective:** We sought to determine those patients with known Fallot tetralogy at risk for progressive dilatation of the thoracic aorta and explore the common predictors present in this patient group.

**Methods and Results:** A multicenter observational study which enrolled 100 patients (50 surgically repaired and 50 before surgical repair of nTOF) with standardized reassessment of echocardiographic parameters and multislice CT angiography of the heart and great vessels data. The data were reviewed and analyzed according to the demographic, morphological, surgical, and clinical details. We used standard nomograms and Z-score for aortic root dimensions at the level of aortic annulus, sino-tubular junction and sinus of Valsalva based on body surface area. For surgically repaired patients, all the measured diameters across aortic annulus, STJ, and sinus of Valsalva were larger in the dilated unrepaired group with mean and median of 24.63 (3.99) and 25 (15–35), 27.2 (4.26) and 27 (17–40), 35.97 (4.59) and 36 (24–45) mm, respectively, compared to a mean and median of 13.2 (2.62) and 13 (9–17), 14.53 (2.90) and 14 (10–19), 20.53 (3.40) and 21 (14–25) mm, respectively, in the not dilated unrepaired group with significant statistical difference (p value < 0.0001). Also Z-score among unrepaired dilated TOF patients was larger in comparison to the non-dilated unrepaired group with significant statistical difference (p value < 0.0001). For unrepaired patients, all the measured diameters across aortic annulus, STJ, and sinus of Valsalva were larger in the dilated unrepaired group with mean and median of 24.63 (3.99) and 25 (15–35), 27.2 (4.26) and 27 (17–40), 35.97 (4.59) and 36 (24–45) mm, respectively, compared to a mean and median of 13.2 (2.62) and 13 (9–17), 14.53 (2.90) and 14 (10–19), 20.53 (3.40) and 21 (14–25) mm, respectively, in the not dilated unrepaired group with significant statistical difference (*p* value < 0.0001). Also Z-score among unrepaired dilated TOF patients at the level of annulus, STJ, and sinus of Valsalva was larger in comparison to the non-dilated unrepaired group with significant statistical difference (*p* value < 0.0001).

**Conclusions:** The first important finding of this study is the occurrence of significant aortic root dilatation in 22% of patients after intracardiac repair of TOF. Older age at repair, long shunt to repair interval, and residual ventricular septal defect are the most common variables associated with aortopathy and aortic regurgitation in such group of patients. The second important finding is the occurrence of aortic root dilatation in 70% of patients before surgical repair of TOF; whereas male sex and TOF with pulmonary atresia appeared to be the most common variables associated with aortopathy and aortic regurgitation in this group of patients.

**Keywords:** Tetralogy of Fallot, Aortopathy, Ventricular Septal Defect, Aortic Regurgitation, Pulmonary Regurgitation, Pulmonary Atresia

## 11

### Radiation Savings in Heart Transplant Patients Using MR-Guided Right-Heart Catheterization Technique

Jennifer Schramm, Ileen Cronin, Laura Olivieri, Tacy Downing, Joshua Kanter

Children’s National Hospital, Washington, USA

**Abstract**

**Background:** Lifetime radiation exposure for heart transplant patients is significant due to need for ongoing surveillance biopsy and coronary angiography. Magnetic resonance (MR)-guided right-heart catheterization (RHC) uses rapid MR image acquisition and reconstruction to aid catheter manipulation, eliminating radiation exposure from the hemodynamic catheterization. However, the magnitude of radiation reduction is unknown. The aim of this study is to understand the typical radiation reduction associated with MR-guided RHC and to quantify the additional contribution from x-ray-guided endomyocardial biopsy (EMB) and coronary angiography.

**Methods:** This is an IRB-approved, retrospective review of patient radiation exposure during surveillance catheterization in 16 transplant patients. Twenty-four MR-guided RHC procedures were weight and age matched with 24 controls with non-complex, 4-chamber circulations who underwent x-ray-guided RHC. Radiation doses for EMB and coronary angiography were also collected from the MR-guided RHC group. The average dose area product (DAP) per kg was calculated and effective dose was estimated using previously published conversions.

**Results:** The median radiation exposure for an x-ray guided RHC was 0.4 uGy*m^2^/kg (range 0.05–3.3). When converted to effective dose, the median total dose was 0.07 mSv (0.006–0.4). In the MR-guided RHC cohort, where patients underwent EMB only (*n* = 9), a median of 1.06 uGy*m^2^/kg (0.2–12) or 0.2 mSv (range 0.02–2) of exposure occurred. In the same cohort for patients who underwent EMB with coronary angiography (*n* = 15), there was a median of 26.0 uGy*m^2^/kg (9–82) or 4.0 mSv (range 1.3–10.6) of exposure.

**Conclusions:** MR-guided RHC is increasingly used to improve visualization and decrease radiation exposure in patients requiring cardiac catheterization. However, the absolute radiation reduction of MR-guided hemodynamics is minimal. In this population, at least 55 MR-guided RHCs would be required to offset the radiation exposure from one annual coronary catheterization. Research efforts should focus on developing MR-conditional equipment for EMB and coronary angiography.

## 12

### Cardiac Catheterization for Hemoptysis in a Children’s Hospital Cardiac Catheterization Laboratory: A 15-Year Experience

Takeshi Sasaki^1,2^, Tomas Forbes^1^, Robert Ross^1^, Yuki Kawasaki^1,2^, Daisuke Kobayashi^1^

^1^Division of Cardiology, Children’s Hospital of Michigan, Detroit, USA. ^2^Department of Pediatric Cardiology, Osaka City General Hospital Pediatric Medical Center, Osaka, Japan

**Abstract**

**Objectives:** The aim of this study was to evaluate the diagnostic utility of cardiac catheterization and the efficacy of transcatheter intervention in patients with hemoptysis.

**Background:** Cardiac catheterization may play a role on identifying the etiologies of hemoptysis with the potential for transcatheter intervention.

**Methods:** This was a retrospective study of all the patients who were brought to the pediatric cardiac catheterization laboratory for the indication of hemoptysis over a 15-year period (2006–2020).

**Results:** Twenty-one patients underwent 28 cardiac catheterizations. The median age was 17.4 years (range 0.3 to 60.0), and the underlying cardiac diagnoses were normal heart *n* = 3, pulmonary hypertension 1, heart transplant 1, pulmonary arteriovenous malformation 1, pulmonary vein disease 3, biventricular congenital heart diseases 5, and single ventricles 7. The diagnostic utility of catheterization was 81% (17/21). At two-thirds (18/28) of catheterizations, transcatheter interventions were performed in 14/21 (67%) patients: aortopulmonary collateral embolization 14, aortopulmonary and venovenous collateral embolization 1, and pulmonary arteriovenous malformation embolization 3. Although recurrent hemoptysis was frequent (50%) post intervention, the final effectiveness of transcatheter interventions was 79% (11/14 patients). Overall mortality was 19% (4/21), all in those presenting with massive hemoptysis.

**Conclusions:** Cardiac catheterization was shown to have good diagnostic utility for hemoptysis especially in patients with underlying congenital heart disease. Despite the high mortality and recurrent hemoptysis rate, transcatheter interventions was effective in our cohort.

## 13

### PDA Stenting in Duct-Dependent Pulmonary Circulation by Different Approach: Single-Center Experience

Hala Agha, Osama Abd El Aziz, Sahar Shaker, Amal El-Sisi, Sonia El Seidi, Ola Kamal, Rodina Sobhy, Aya Fatouh, Amira Esmat

Cairo University, Cairo, Egypt

**Abstract**

**Background:** The implantation of stents to keep the ductus arteriosus patent in cyanotic congenital heart disease with duct-dependent pulmonary circulation is an alternative to the modified Blalock–Taussig surgery.

**Objectives**: To study the feasibility and outcome of ductal stenting by different approach in patients with duct-dependent pulmonary circulation.

**Patients and Methods:** Between January 2018 and December 2019, ninety-six patients with duct-dependent pulmonary circulation underwent PDA stenting as first palliative procedure. Post-procedure follow-up was done by clinical evaluation, assessment of Sao2, and echocardiography evaluation for the stent patency and the size of the pulmonary arteries. These parameters were compared with baseline values.

**Results**: The median age of 96 patients was 22 (2–120) days and their median weight was 3 (2–5.5) kg. The procedure was successful in 81.25% of patients, 72.92% has tortuous PDA. The femoral artery approach was used in 75% of procedures, axillary access in 19.79%, carotid access in 3.8%, and femoral vein approach in 2.6%. Seventy-nine coronary stents were deployed in 78 patients. One patient needs two stents to cover total ductal length, and drug-eluting stent was used in 17 (21.8%) patients. Median right PA (RPA) and left PA (LPA) Z-scores were − 0.79 (− 1.91 to 0.12) and − 1.02 (− 1.82 to − 0.38), respectively. The success rate was higher in straight PDA and in relatively younger patients. Post-stenting mean oxygen saturation (SO_2_) increased significantly from baseline. No mortality was reported during the procedure. Minor complications in seven patients, while stent thrombosis in two patients and stent embolization in another two. At a median follow-up (4–6) month for 43 patients, MSCT was done for 37 patients, median RPA and LPA Z-scores, [0.93 (0.28–1.82), 0.40 (0.24–1.01)], respectively, were significantly increase compared to baseline. Four patients required reinterventions. Eighteen patients underwent cardiac surgery during the follow-up period. Urgent BT shunt was done for six patient, palliative surgery was done for nine, and corrective surgery for three patients.

**Conclusion**: Ductal stenting is an effective palliative procedure for patients with duct-dependent PBF. It maintains adequate SO2 and promotes balanced PA growth at mid-term follow-up.

**Keywords:** Duct-dependent pulmonary circulation, PDA stenting, Approach, Outcome

## 14

### Silent Patent Ductus Arteriosus, Shall We Treat?

Saif Aljemmali, Sawsan Awad

Rush University Medical Center, Chicago, Illinois, USA

**Abstract**

**Introduction:** Hemodynamically insignificant patent ductus arteriosus (PDA) defined as PDA that does not cause congestive heart failure or left atrium and left ventricle dilation. Hemodynamically insignificant PDA can produce turbulent high-velocity flow across its lumen, thereby increasing the risk of bacterial endarteritis. Therefore, the majority of pediatric cardiologists recommend closure of hemodynamically insignificant PDAs that produce an audible murmur. However, closure of non-audible small PDAs remains controversial.

**Purpose:** This study aims to evaluate the current general management of non-audible patent ductus arteriosus (PDA) that persists beyond infancy. We hypothesize that non-audible hemodynamically insignificant PDAs have turbulent high-velocity blood flow passing through them more or less similar to audible PDAs, therefore, carrying the same risk for developing infective endarteritis.

**Methodology:** This is a retrospective chart review of all pediatric patients ≥ 9 months of age with hemodynamically insignificant PDAs in the period between July 2009 and July 2019 at Rush University Medical Center. Patients were divided into audible PDA group and non-audible PDAs group. Flow velocity, aortic, and pulmonic diameters of PDAs were examined and compared in both groups.

**Results:** Fifteen patients were included. Ten patients (~ 67%) had non-audible hemodynamically insignificant PDAs, and five patients (~ 33%) had audible hemodynamically insignificant PDAs. There were no statistical differences between flow velocities, aortic, and pulmonic diameters of non-audible PDAs versus audible PDAs (*p* value = 0.12, 0.07, and 0.52, respectively).

**Conclusion:** Hemodynamically insignificant PDAs, whether audible or non-audible, have the same risk stratification of developing infective endarteritis and should have identical ways of management.

## 15

### Catheter Intervention as a Palliative procedure in Children and Adolescents with Pulmonary Hypertension

Amal El-Sisi, Mohamed Samir, Mohamed Mossad

Cairo University Children Hospital, Cairo, Egypt

**Abstract**

Pulmonary Hypertension is defined by a threefold condition: a mean PAP of 20 mmHG, a LAP lower than 15 mmHG, and a PVR of 3 Wood units. A lot of progress has been achieved when it comes to medications allowing for longevity and a better quality of life for PH patients; however, these medications are not curative. Interventions are also palliative as patients would eventually need lung or heart–lung transplants. Interventions include septostomies or stenting the interatrial septum. More recently, reversed Potts shunts—both surgical and transcatheter—have also been performed on PH patients. Other PH interventions can be applied for cases of univentricular repair such as Glenn and Fontan procedures. These may include blocking extra-pulmonary blood supply, or interrupting forward flow, using plugs or other devices. For Fontan cases, circulation opening and stenting the closed fenestration can also help, especially when Fontan intervention fails.

Interventions in PH in children and adolescents are not common. We report 20 cases who experienced different interventions for PH, with an age range of 9–25 years old and 75% female patients. Of the 20, 10 had idiopathic Ph, 4 were post Glenn, 2 post PA band, and 4 post Fontan. Interventions include balloon dilatation of IAS (6), stent IAS (3), Stent ASD device (1), Shunt occlusion (2), Stent PDA coil (1), PDA occlusion (1), and forward flow interruption (6). Follow-up of idiopathic PH group revealed an overall improvement in symptoms and an increased desaturation. As for the univentricular heart group, symptoms also improved and more prominently, these cases exhibited a drop in PH. Through these cases, we conclude that intervention for PH in children and adolescents is a safe and feasible palliative procedure, which improves symptoms in PH for both idiopathic and post-surgery univentricular heart patients.

## 16

### Monastir Left Main Stenting Registry (Mona Main): Left Main Stenting Registry in Fattouma Bourguiba University Hospital in Tunisia Cardio B Department: Retrospective Study (2018, 2019)

Karim Fahmy

Helwan University, Cairo, Egypt

**Abstract**

**Review of Literature:** Percutaneous treatment of unprotected left main coronary artery (LMCA) disease has undergone a remarkable evolution over the past few decades. The first case series of angioplasty procedures reported by Grüntzig in 1979 included treatment of unprotected LMCA disease. Early experience, however, demonstrated high early mortality and poor long-term outcomes after balloon angioplasty of unprotected LMCA disease.

**Aim of the Study and Methodology:** Two years of retrospective registry for the patient underwent left main stenting in Fattouma Bourguiba University Hospital Cardio B Department are investigated regarding the procedure and provisional vs 2-stent technique, which technique is used in bifurcation stenting.

**Results and Discussion:** The patients were males (80%), diabetics (50%), and radial access was used in 60% in of cases with no significant bleeding complications either from Femoral or Radial access. The left main was affected ostially in 70% of cases and was treated by stenting while in 30% the left main was affected distally and also was treated by stenting. The patients were presented by anterior myocardial infarction in 40% of cases, while NON-STEMI in 40% of cases. In 30% of cases, the patients were stented in other sites than left main before stenting in the left main also 10% were stented in the left main before and we faced in-stent restenosis and were treated by stenting with excellent result and follow-up in clinic. In 10% of cases we treated by using 2-stent technique (TAP) technique and 90% were treated by 1-stent provisional stenting with TIMI III Flow in both LAD and LCX. We had 1 case died on table which was presented by anterior myocardial infarction thrombolysed 8 h after presentation arrested after stenting and died on table 40% of cases were treated by pre-dilation before stenting while 60% undergone as direct stenting with excellent TIMI III distal flow. 50% of cases had post-dilatation after stenting with excellent TIMI III distal flow. 30% of cases were treated by Sirolimus-eluting stents while 70% were treated by Everolimus-Eluting stents with excellent follow-up for all cases in our outpatient clinic.

**Conclusion:** Left main stenting is uprising method for treatment of left main stenosis vs CABG. The second-generation drug-eluting stents are willing regarding TLR and MACE with less bleeding complication in radial and femoral access.

**References**

1. O’Keefe J.H. Jr., Hartzler G.O., Rutherford B.D., et al. (1989) Left main coronary angioplasty: early and late results of 127 acute and elective procedures. Am J Cardiol 64:144–147.
2. Park S.J., Park S.W., Hong M.K. et al. (1998) Stenting of unprotected left main coronary artery stenoses: immediate and late outcomes. J Am Coll Cardiol 31:37–42.

## 17

### Feasibility of Device closure of Ostium Secundum Atrial Septal Defect(OS ASD) in Less Than 1-Year-Old children—A Single-Center Experience

Dr. M. Kalyanasundaram, Pediatric cardiologist, G.K.N.M. Hospital, Coimbatore, India

**Abstract**

Herein we report successful device closure of large-sized OS ASD in 59 children with less than 1 year of age done over the period of 9 years. The study population age and weight ranged from 7½ to 12 months and 5.7 to 10.3 kg. Symptomatic children with dilated right heart were taken as criteria for the device closure. The sizes of the devices used were from 10 to 22 mm with high rate of successful deployment (97%). All patients who underwent successful device closure tolerated the procedure well without any major complications

**Introduction:** Device closure of OS ASD in younger children with larger defects is a challenging task in view of requiring larger-sized sheaths and devices and thin pliable rims compared to older children. The size of the defects more than 8 mm are categorized large-sized defects.

**Materials and Methods:** Fifty-nine children (male 30 and female 29) with age group of 7 ½ months to 1 year and the weight ranged from 5.7 kg to 10.3 kg were included in our study population. We used Amplatzer septal occluder and delivery system in all our cases. All the devices were deployed using mostly left and in some cases right upper pulmonary venous approach under the guidance of TTE, TEE, and fluoroscopy. In 20 out of 59 children, we did not use TEE.

**Results:** Out of 61 children taken for the device closure of ASD, 2 children did not undergo the procedure due to failure to position the device. Fifty-nine patients had successful device closure.

**Discussion:** We had to up size the device in 5 and downsize in 2 patients after we found the device were mismatched on fluoroscopy. This was probably due to difficulty in identifying the thin atrial septal rims in echocardiogram. During the procedure none experienced major complications. But few patients developed premature atrial complexes and two cases had atrial bigeminy which subsided within 12 h of the procedure. The sizes of the devices selected were 2 to 3 mm more than the actual defect size measured by either TTE or TEE. The sizes of the devices used ranged from 10 to 22 mm. Out of 59 patients, 18 patients received 10 to 14 mm, 29 patients received 16 to 20 mm, and 2 patients received 22 mm devices. The patients were discharged from the hospital in the next day with dual antiplatelets.

**Advantages in early closure:**

Thin and floppy margins were our concerns but all had successful deployment. Delayed intervention resulted in unsuitable for device closure due to resorption of thin margins especially IVC rim.
2. Early intervention avoided recurrent OPD visit or admissions there by saving the total cost of treatment.
3. Low risk of complications with experienced operator.

**Conclusion:** Since the transcatheter closure of ASD is feasible, relatively safer, and cheaper in children less than 1 year of age, there is no need of delaying the closure of the large-sized ASDs to the later age.

## 18

### Unexpected Complication of Fontan Operation Causing Cyanosis, and Impaired Coronary Circulation

Jacek Kusa^1,2^, Pawel Czesniewicz^2^, Magdalena Slupska^2^

^1^Pediatric Cardiology Department, Medical University of Silesia, Katowice, Poland. ^2^Pediatric Cardiology Department, Regional Specialized Hospital-Research and Development Centre, Wroclaw, Poland

**Abstract**

**Background:** Patients after Fontan procedure sometimes develop extremely rare complications, most of which can be treated in the cathlab.

**Materials and Methods:** The 9-year-old boy after Fontan completion 7 years earlier, percutaneous closure of fenestration and aortopulmonary collateral (in 4th and 6th year of life, respectively) was admitted to our hospital due to increasing desaturation (from 98 to 88%) and worse exercise tolerance with increasing fatigue. ECG revealed ST-segment elevation in precordial leads. In the performed angiographic and hemodynamic examination, the properly functioning Fontan circulation was confirmed, but at the same time the presence of a fistula between the extracardiac tunnel and the coronary sinus was revealed. Fistula had a diameter of 4.5 mm and a length of 14 mm. It connects to the coronary sinus through a network of numerous vessels. Blood flow from the fistula caused stasis of blood in the coronary circulation.

The procedure was performed under general endotracheal anesthesia, through femoral vein access. The right coronary Judkins catheter was used to enter to the ostium of fistula. The manual angiography was performed to confirm the previous diagnosis and establish the morphology precisely. Then the catheter was inserted deeper, but just before the coronary sinus. Through the same catheter the Amplatzer Vascular Plug 4–7 mm was introduced and released from delivery system. The right position of the implant was confirmed. Over the next 3 days the infusion of heparin was maintained, and then acenocoumarol was introduced with the recommendation lifetime use.

**Results:** During follow-up, the increase of arterial saturation till 97% was observed and normalization of ST-segment in all leads was found. The physical efficiency improved significantly.

**Conclusions:** The appearance of new symptoms of unknown etiology in patients with Fontan circulation should always be an indication for cardiac catheterization. In most cases, it is possible to find the source of the cyanosis and percutaneously close it. Some abnormal vascular connections can also lead to coronary ischemia, which can be easily treated in the same way.

## 19

### Cardiac Catheterization During Early Postoperative Superior Cavopulmonary Anastomosis Procedure: Nicklaus Children’s Hospital Experience

Saleem Almasarweh, Jun Sasaki, Daniel Duarte, Lourdes Prieto, Patcharapong Suntharos

Nicklaus Children’s Hospital, Miami, USA

**Abstract**

**Background:** Despite the improvement in surgical techniques and postoperative care, cardiac catheterizations are frequently performed in the postoperative period after superior cavopulmonary anastomosis (SCPA) procedure. Our objectives are to describe types of interventions, indications, and characteristics of patients who required early catheterization.

**Methods:** We retrospectively reviewed all patients who underwent SCPA from January 2006 to December 2019. Preoperative data and postoperative course were reviewed. Early post-SCPA cardiac catheterization was defined as catheterization performed before discharge.

**Results:** A total of 195 patients underwent SCPA during the study period, 120 (61.5%) were male. One hundred and twelve patients (57.4%) had single right ventricle, 57 patients (29.2%) had single left ventricle, and 26 patients (13.3%) had two ventricles that could not be separated. Bilateral superior vena cava (SVC) was found in 24 patients (12.3%) and interrupted inferior vena cava was found in 7/195 (3.5%). In the interstage period, 51 patients (26.2%) underwent at least one additional cardiac catheterization, 32/51 (62.7%) required an intervention. During pre-SCPA cardiac catheterization, 28/194 patients (14.4%) underwent an intervention, the median mean PA pressure was 14 mmHg (9–24 mmHg) with a median transpulmonary gradient of 7 mmHg (2–19 mmHg), the median single ventricle end-diastolic pressure was 9 mmHg (4–19 mmHg), the median SVC and systemic saturations were 51% (26–75%) and 78% (45–94%), respectively. The median age and weight at the time of SCPA were 166 days and 6.2 kg, respectively. The median post-SCPA length of stay (LOS) was 8 days with 3% early mortality (6/195). Thirty-two patients (16.4%) underwent 43 post-SCPA catheterizations, 5 patients had 2, 1 patient had 3, and 1 patient had 4 catheterizations. The median postoperative day when the first catheterization was performed was 6.5 days (1–90 days). The indications were hypoxia in 32 patients, persistent pleural effusions in 11 patients, hemodynamic instability requiring ECMO support in 3 patients, aortic arch obstruction in 2 patients, SVC syndrome in 1 patient, and over circulation due to an additional aortopulmonary shunt in 1 patient. Five procedures were hemodynamic evaluation only. Fifty interventions were performed, the most common interventions were pulmonary artery stent angioplasty (40%), pulmonary artery balloon angioplasty (20%), aortic arch balloon angioplasty (12%), venovenous collateral occlusion (10%), and aortopulmonary collateral occlusion (6%).

Patients were divided into two groups with (group A) and without (group B) early postoperative catheterization. Group A had longer post-stage 1 LOS (mean 43 vs 28 days, *p* = *0.05*), additional catheterizations in the interstage period (43% vs 22.7%, *p* = 0.01), and lower pre-SCPA SVC saturation (mean 46% vs 51%, *p* < 0.01). At the time of SCPA, group A was younger (mean 162 vs 199 days, *p* < *0.01*) with lower weight (mean 5.8 vs 6.4 kg, *p* = 0.03) and longer cardiopulmonary bypass time (126.9 vs 91.6 min, *p* < 0.01).

**Conclusions:** Early post-SCPA cardiac catheterization is common. Several interesting characteristics were found to be significantly different in patients who required early catheterization.

## 20

### Spontaneous closure of PDA after unsuccessful transcatheter attempts to close PDA in preterm babies

Ahmed Deniwar, Duraisamy Balaguru

Division of Pediatric Cardiology. University of Texas McGovern Medical School at Houston, Houston, USA

**Abstract**

**Background:** Patent ductus arteriosus (PDA) is much more common in preterm infants, due to multiple factors. Transcatheter closure of PDA in preterm babies is increasing after approval of Amplatzer Piccolo device in the United States. We report two babies in whom spontaneous closure of PDA occurred after unsuccessful transcatheter closure attempts.

**Case summary:** Patient 1 is 3-month-old, ex-28-week preterm girl. Current weight 3.5 kg. The PDA was tubular (Type C) with diameter 4.4 mm. Amplatzer Piccolo 5-4 device was placed in PDA. Overnight, baby remained intubated and sedated using Dexmedetomidine. Next morning, chest X-ray, Echocardiogram, and CT scan confirmed embolization of device to left pulmonary artery (LPA). Repeat catheterization was performed and the embolized device was retrieved. Amplatzer duct occlude device (6/4) was deployed, but was also removed due to unsatisfactory position. Decision was made not to pursue further attempts at transcatheter PDA closure. Echocardiogram next morning showed a much smaller PDA and repeat echocardiogram 5 days later showed complete closure of PDA. (There has been a follow-up echocardiogram before discharge that showed closed PDA).

Patient 2 is 5-week-old ex-28-week preterm boy. Current weight 1.26 kg. Multiple dysmorphic features led to diagnosis of Cornelia de Lange syndrome. By angiogram, PDA was type F (Hockey-stick shape) with diameter 4 mm. Amplatzer Piccolo Occluder (5-2) device was deployed. After echocardiographic confirmation, the device was released. Upon release, the device was noted to move back into main pulmonary artery (MPA). Therefore, attempts were made to retrieve the device using gooseneck snare. During these attempts, the device dislodged to LPA and pericardial effusion occurred. After pericardiocentesis, there was immediate recurrence. Sternotomy was performed and a perforation in RVOT was repaired. Next morning, echocardiogram showed no PDA, device in proximal LPA with mild LPA stenosis only. At 4 months after procedure, device is still in MPA/LPA junction with mild LPA stenosis.

**Discussion:** We are presenting two preterm babies who had unsuccessful attempts at transcatheter device closure of PDA and subsequently found to have spontaneous closure in both babies—one in 5 days and the other within 24 h. We speculate that mechanical stimulation of wall of PDA by the devices may initiate a process of spontaneous PDA closure. Other mechanisms for spontaneous closure of the PDA after birth including high pO_2_ and low circulation prostaglandin have been proposed. Further studies are required to delineate the mechanism of spontaneous closure.
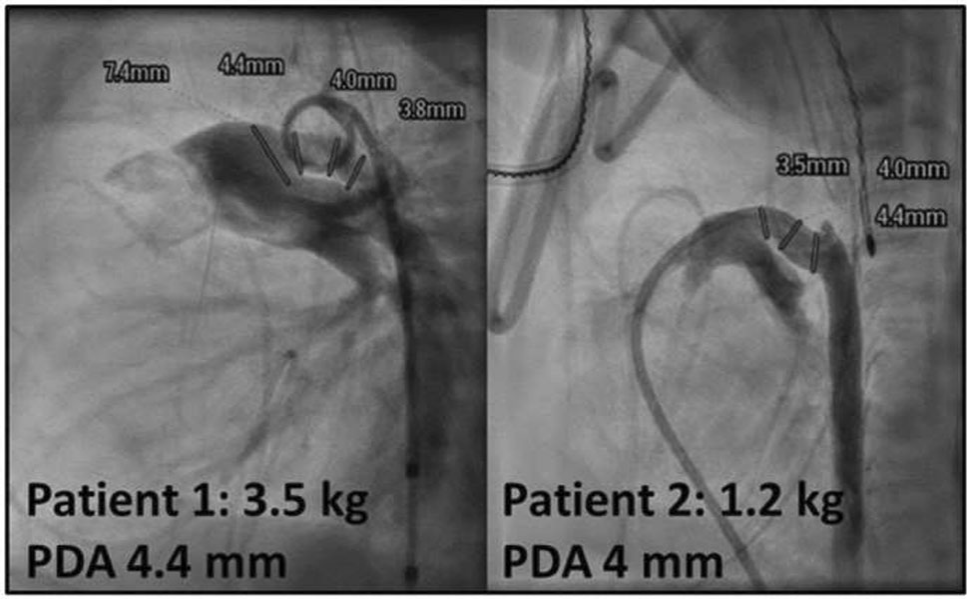


## 21

### Transcatheter Mitral Valve-in-Valve Implantation with an Edwards SAPIEN 3 Valve in an Adult with Congenital Heart Disease

Heike Schneider, Claudius Jacobshagen, Thomas Paul, Claudia Dellas

University Medicine Goettingen, Goettingen, Germany

**Abstract**

Transcatheter valve-in-valve (ViV) implantation has emerged as an alternative treatment option to repeat surgical replacement in high-risk patients for degenerated bioprosthetic valves. But data regarding these procedures in adults with congenital heart disease (ACHD) remain limited.

We present the case of a 60-year-old lady born with a partial atrioventricular septal defect that was surgically corrected at the age of 7 years. Left-sided AV-valve replacement was performed for significant left-sided AV-valve stenosis and moderate regurgitation with a bioprosthetic valve (CE Perimount Modell Magna Mitral ease (25 mm) at the age of 54 years. The bioprosthesis was favored over mechanical valve replacement because of systemic lupus erythematosus with cerebral vasculitis and increased risk for cerebral bleeding to avoid long-term anticoagulation with a vitamin K antagonist. The patient was kept on 100 mg ASS once daily. Comorbidities included arterial hypertension, chronic renal insufficiency, moderate aortic stenosis, and regurgitation.

Five years later, the patient complained about dyspnea and decreased exercise tolerance due to severe stenosis of the bioprosthesis with a mean echocardiographic gradient of 12 mmHg. Because of high-risk for open-heart surgery transcatheter ViV implantation was favored.

Cardiac catheterization was performed under general anesthesia, heparin was given to achieve an ACT > 200 ms and cephazolin was administered intravenously as prophylaxis. Transseptal puncture guided by TEE was followed by balloon dilation of the intraatrial septum with an 8 mm Atlas balloon. Under 3D-TEE guidance the stenotic mitral valve was crossed, and a ‘Safire’ wire was placed into the LV apex. To allow unhindered ViV implantation, the stenotic calcified bioprosthetic valve was predilated with the ES balloon. By previous CT evaluation and using the App ‘ViV Mitral,’ it was decided to implant a 26 mm ES 3 valve. This was subsequently performed without any problems. The 26 mm ES 3 valve was placed in good position, there was no insufficiency and the mean gradient was reduced to 3 mmHg post implant. Postinterventional atrial fibrillation was successfully cardioverted and gastric bleeding required gastroscopic clipping and transfusion. Anticoagulation was changed from initial dual antiplatelet therapy to apixaban 5 mg bid. At 3 months of follow-up, the patient’s clinical status has improved significantly and the mean gradient across the ES 3 valve remained between 3 and 4 mmHg.

**Conclusion:** Transcatheter mitral ViV implantation may be a good option in selected high-risk ACHD patients with good short-term results and low complication rate. Long-term data of transcatheter atrioventricular ViV implantation, originally developed for the aortic position, are still lacking.

## 22

### Successful Transcatheter Balloon Valvuloplasty of Previously Placed Transcatheter Pulmonary Valve Years After Initial Placement: A Report of 2 Cases

Andrea Otero Luna, Britton Keeshan, Jeremy Asnes

Yale University, New Haven, USA

**Abstract**

**Background**: Transcatheter pulmonary valve replacement (TPVR) is currently an accepted technique to treat conduit and bioprosthetic valve failure. Residual or progressive gradient across the transcatheter valve is thought to be a risk factor for development of bacterial endocarditis and RV dysfunction. Outcomes of balloon valvuloplasty years after initial TPVR to address residual stenosis are unknown.

**Methods**: We report our recent experience in 2 patients with moderate to severe obstruction across the Melody valve who underwent successful balloon valvuloplasty after more than 4 years from initial implantation

**Results**: Two patients were included in this series. Patient one was 14 years old with VACTERL sequence and pulmonary atresia, VSD, confluent central pulmonary arteries, and right aortic arch. Initial palliation included a central shunt when he was 6 days old with subsequent Rastelli procedure with VSD closure and RPA augmentation when he was 6 months old. At 30 months, patient had RV-PA conduit replacement with 17 mm aortic homograft for severe conduit obstruction. Patient developed moderate conduit obstruction and at 5 years of age, patient underwent successful TPVR with an 18 mm Medtronic Melody Valve following pre-stenting. There was no residual gradient across Melody valve following TPVR. Overtime the patient developed progressive stenosis across the valve consistent with somatic growth with multiple admissions for aspiration pneumonia and bacterial endocarditis rule-outs. At 14 years old, there was moderate stenosis by echocardiogram (peak 32 mmHg, mean 19 mmHg) and decision was made to perform balloon valvuloplasty. Patient underwent successful valve expansion up to 22 mm with an excellent result. There was trivial pulmonary insufficiency by intracardiac echo and no residual gradient. There were no complications.

Patient two was a 13-year-old patient with complete atrioventricular canal defect with tetralogy of Fallot who underwent complete surgical repair in the newborn period with VSD closure and a transannular patch. He subsequently developed recurrent stenosis in his right ventricular outflow tract and had repeat surgical intervention at 4 months old. He developed progressive stenosis across his RV outflow tract patch and underwent successful TPVR with 18 mm Medtronic Melody valve at 7 years old following pre-stenting with 5 mmHg residual gradient at end of procedure. Four years later he was noted to have severe stenosis across the Melody valve (peak 70 mmHg, mean 38 mmHg). The Melody valve was subsequently balloon dilated using a 20 mm high-pressure balloon with resultant 2 mmHg gradient and mild Melody valve insufficiency. There were no complications.

**Conclusion**: Successful balloon valvuloplasty of melody valve is feasible even after 9 years from initial implantation of the Melody valve without complications or significant valve compromise.

## 23

### Pulse Loss After Cardiac Catheterization in Infants: Does Sheath Size Matter?

Kaitlin Swanson, Jillian Gorski, Mark Hoyer, Michael Ross, Ryan Alexy

Indiana University, Indianapolis, USA

**Abstract**

**Objective:** The aim of this study was to review the prevalence of pulse loss in infants after cardiac catheterization at our institution, and to understand the benefit, if any, of using small 3.3 French sheaths in this population.

**Methods:** A retrospective cohort study including all infants 0–12 months of age who had a cardiac catheterization with femoral arterial access at our institution between January 2015 and June 2019 was performed. Variables included patient age, weight, case duration, and size of arterial sheath. The primary outcome was clinical pulse loss. Secondary outcomes included treatment with heparin and diagnosis of an arterial thrombus. We used Fisher’s Exact and Chi-squared tests for comparing the incidence of our binary outcomes, clinical pulse loss, and heparin administration, among the different sheath sizes. We performed multiple logistic regression analyses, examining the association of variables with each outcome of interest.

**Results:** 401 patients under 12 months old were analyzed. The prevalence of clinical pulse loss was 14.9%; 6.5% were treated with a heparin bolus, 11.2% had an ultrasound performed for persistent pulse loss, and 7.5% were diagnosed with a femoral arterial thrombus by ultrasound. Of these 401 patients, 82 had a 3.3F sheath, 298 had a 4F sheath, and 21 had a 5F sheath. The prevalence of pulse loss in patients with 3.3F sheaths was 8.5%, compared to 16.8% in patients with 4F sheaths, and 14.2% with 5F sheaths. Fisher’s exact test showed no statistically significant difference among the groups for incidence of pulse loss (*p* value 0.170) or heparin treatment (*p* value 0.379). However, when multiple logistic regression were performed, sheath size was significantly associated with pulse loss; odds ratio for pulse loss was 2.92 (95% CI 1.23–6.95) for 4 Fr catheters compared to 3.3 Fr catheters (*p* value = 0.015). A similar trend was found for treatment with heparin; odds ratio for administration was 2.46 (95% CI 0.69–8.69) for 4 Fr catheters compared to 3.3 Fr catheters, however, this did not meet our threshold for significance (*p* value 0.164).

**Conclusions:** Pulse loss following cardiac catheterization in children is a relatively common complication, especially in babies with small femoral arteries. Historically 4F sheaths were the smallest sheaths available, however, during the last decade, smaller sheaths have been developed. This retrospective study showed that in this high-risk patient population of small infants, a smaller 3.3 Fr sheath (Super Sheath, PediaVascular) is less likely to cause pulse loss than a standard 4F sheath. Since the conclusion of this study, our institution has routinely used the Prelude Ideal sheath from Merit Medical which accepts 4F catheters but has the outer diameter of a typical 3F sheath. The hope is this even smaller sheath will further decrease the prevalence of pulse loss in this vulnerable population.

## 24

### Hybrid Placement and Subsequent Hybrid Successful Repositioning of Unbuttoned Amplatzer Septal Occluder Through Anterior Mini-Thoracotomy in a Premature Infant

Rupesh Kumar Natarajan, Varun Aggarwal, John Bass, James Berry, Massimo Griselli, Gurumurthy Hiremath

University of Minnesota, Minneapolis, USA

**Abstract**

A 4-month-old female, former 24 weeks, weighing 4.0 kg, with moderate secundum atrial septal defect (ASD) in the setting of severe respiratory disease of prematurity and pulmonary hypertension (on inhaled nitric oxide and sildenafil). Baseline hemodynamics were significant for moderately elevated pulmonary artery pressures (mPAp 27 mmHg) and normal PVR 2.3 iWU improved with vasoreactivity testing. Due to a small patient size and a tortuous thin IVC, a decision was made to close the ASD per-atrially through a hybrid anterior mini-thoracotomy approach. A regular mini TEE probe could not be passed and a ‘micro TEE’ probe was used which limited the ability to get good images. By TEE, the defect measured 0.6–0.7 cm with adequate rims all around except the retro-aortic rim (2 mm).

Surgical right anterior mini-thoracotomy through a 5th intercostal space was performed and purse string suture was placed in the right atrium. An 18 gauge needle and a 0.035″ Emerald J tip wire was advanced through the purse string across the ASD and positioned tip in the left lower pulmonary vein using echo guidance. A 6 Fr short sheath, hand shaped to provide torque capability, was advanced over the wire and tip positioned in the left atrium. An 8-mm Amplatzer Septal Occluder (ASO) (St Jude Medical Inc.), prepped in usual fashion, was deployed under TEE guidance with no residual atrial level shunting although the images were suboptimal with the micro TEE probe.

At follow-up, the occluder was appeared to have missed catching the superior and posterior rim, 2 days later it was malpositioned and had unbuttoned with the right atrial disc partly in the left atrium. The child was taken back to the cath lab, under TEE guidance, an 8 fr sheath was introduced through the same anterior mini-thoracotomy incision and through the right atrial purse string suture. A 10-mm Amplatz Gooseneck Snare (eV3 Endovascular, Inc.) was positioned over the microscrew and snared. The right atrial disc was partially recaptured, and device redeployed across the atrial septum. Epicardial echocardiography was used in conjunction with TEE which confirmed good capture of all rims. Child was extubated after 6 days and was weaned off CPAP 12 days later. At 3 weeks of follow-up, the device was well seated with small residual shunt through the waist and was discharged home.

Dislocation of ASO placed by hybrid approach through mini-thoracotomy has been previously described in two adult studies. Occluder dislodged into the right atrium in all five cases and underwent immediate surgical repair with cardiopulmonary bypass (CPB). In our case, detection of malposition was early when the occluder was still partially attached to the septum. This enabled us to attempt hybrid snare retrieval through mini-thoracotomy which is a novel and feasible approach for malpositioned ASO devices that has not been previously reported. It could be considered as a good alternative to transcatheter percutaneous or surgical retrieval techniques whenever possible given limited exposure to fluoroscopy and avoiding CPB.

## 25

### Clarifying Morphoanatomy of Superior Sinus Venosus Interatrial Communication—A Computed Tomographic Study of 100 Patients

Jay Relan, Saurabh K. Gupta, Rengarajan Rajagopal, Sivasubramanian Ramakrishnan, Gurpreet S. Gulati, Sanjiv Sharma, Shyam S. Kothari, Anita Saxena, Vinay K. Bahl

All India Institute of Medical Sciences, Delhi, India

**Abstract**

**Aims**: Superior sinus venosus defect is the most common extraseptal interatrial communication. The morphology of superior caval vein—right atrium junction and pulmonary veins is variable and determines the modality and the technique of repair. In this study, we sought to clarify the morphoanatomy of sinus venosus interatrial communication using computed tomography.

**Methods and Results**: A total of 100 computed tomographic angiograms of patients with superior sinus venosus defect were systematically analyzed using a pre-defined proforma. The mean age was 31.4 ± 16.9 years (Range—0.33–66 years). The mean transverse and supero-inferior diameters of the sinus venosus defect were 18.4 ± 5.7 mm and 17.0 ± 5.3 mm, respectively. In sixty-nine (69%) patients, superior caval vein was overriding the atrial septum, with more than 50% override in five patients. All patients had anomalous drainage of right superior pulmonary vein. Additionally, right middle and inferior pulmonary vein drained anomalously in 95 and 18 patients, respectively. Thirty-six patients had at least one pulmonary vein from the upper lobe of the right lung connecting to superior caval vein more than 2 cm cranial to the superior caval vein-right atrium junction. Six patients had discrepant drainage of the lung segments of the same lobe with some segments draining to the right atrium while other segments draining normally to the left atrium. Twenty-one patients had an associated persistent left superior caval vein.

**Conclusion:** Morphology of superior caval vein-right atrium junction and connection of anomalously draining pulmonary veins in superior sinus venosus defects were quite variable. The anomalous drainage of pulmonary veins is not limited to right upper lobe. In a large number of patients, middle lobe drains anomalously while a minority also have anomalous drainage of lower lobe. Detailed morphological assessment by computed tomography can help in surgical planning as well as selecting patients suitable for interventional closure.

## 26

### Reducing Transcatheter Valve Endocarditis Incidence with PTFE-Covered “Pre-Stents”

Reid Ponder^1^, Hannah El-Sabrout^1^, Soraya Sadeghi^1^, Arpine Davtyan^2^, Jamil Aboulhosn^3^, Howaida El-Said^2^, Daniel Levi^3^

^1^University of California, Los Angeles, Los Angeles, USA. ^2^Rady’s Children Hospital, San Diego, USA. ^3^Ronald Reagan UCLA Medical Center, Los Angeles, USA

**Abstract**

**Background**: Annualized endocarditis rates as high as 2.3% have been reported following Melody valve transcatheter pulmonary valve replacement (TCPVR). It has been suggested that “pre-stenting” with a PTFE-covered stent can lower the rate of endocarditis.

**Methods**: The experiences at Mattel-UCLA and Rady-UCSD were reviewed for incidence of endocarditis in transcatheter valves. Melody valves implanted with or without a PTFE-covered pre-stent were compared, as well as Melody vs. Sapien valves, which come with a PTFE covering. Kaplan–Meier curves were generated to compare freedom from endocarditis between these different valves and implantation procedures.

**Results**: Since 2010, there were 399 Melody valve and 124 Sapien valve TCPVRs performed between UCLA and UCSD. Of the Melody valve implants, there were 27 performed after placement of a PTFE-covered CP stent. There were no cases of endocarditis with Melody valve implants after use of covered pre-stents and only one case of Sapien valve endocarditis. The Kaplan–Meier curves shown below demonstrate the divergence of endocarditis incidence in valves covered by PTFE or implanted within PTFE stents.
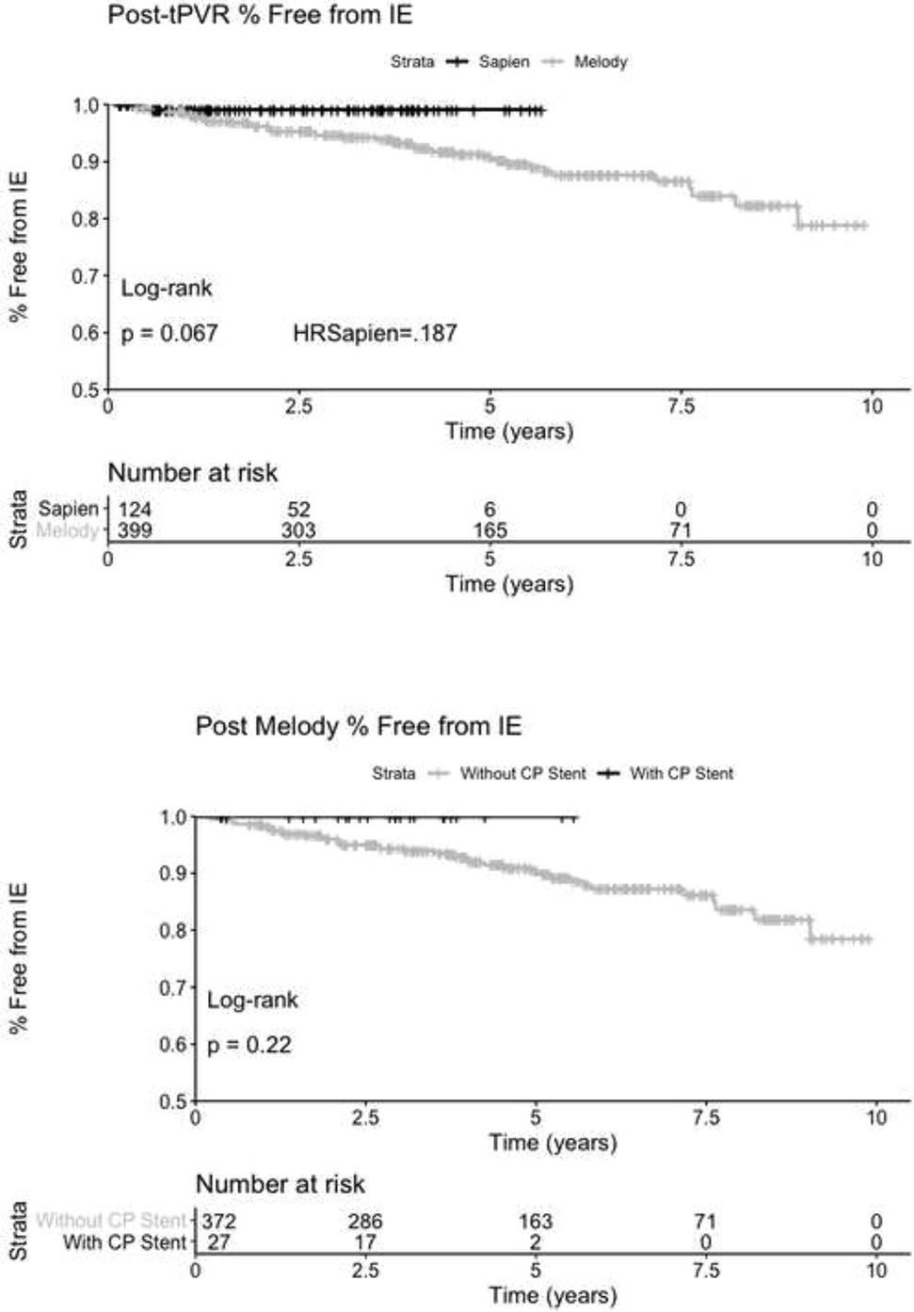


**Conclusion**: In a large dual-center experience, neither Melody valves implanted within PTFE stents nor PTFE-covered Sapien valves showed significant incidence of endocarditis. These patients will continue to be followed to determine if this effect is significant with time.

## 27

### Self-Expanding Nitinol Growth Stent for Neonatal Coarctation

Daniel Levi, Nima Nia

UCLA, Los Angeles, USA

**Abstract**

**Background**: Use of stenting for aortic and pulmonary stenosis in newborns is limited by the exponential growth of vasculature. While the blood vessel grows, the stent does not. Prototype self-expanding stents designed to grow with the aorta were tested in a chronic animal model and the effects of radial force on growing tissue was investigated.

**Methods**: Self-expandable nitinol stents with 4 different radial forces were designed and manufactured. Prototype stents were implanted in the abdominal aorta or iliac artery of four 50 kg, 3-month-old pigs for a duration of 90 or 180 days. Average final weight of animals after 180 days was 139 kg. Prior to explant, stents were assessed by angiography and then by micro-CT and histopathology after explantation.

**Results**: Two stents were explanted after 90 days and the remainder at 180 days. None of the pigs had major complications nor were there issues with deployment, dissection, aneurysm formation, or fracture. Figure A1 and A2 shows the angiography of the native aorta before and 180 days after stenting. Figure B shows a graph of vessel growth; the native vessel grew in the range of 1.4 mm to 3.1 mm/6 months while the stented vessels always grew at higher rates ranging from 1.7 mm to 6.6 mm/6 months. There was a 22–27% growth in diameter for stented vessels versus 13% for native vessels. Histological findings (Figure C) were comparable among all stents with overall mild injury score, and with all stents showing mature neointima and focal mild inflammation without extension to the underlying arterial wall. Higher radial force stents caused asymmetric deformation of arteries with increased, but localized inflammation.

**Conclusion**: Self-expanding stents implanted in the aorta are able to expand in the aorta without inhibiting growth or causing vascular injury. This strategy could have promise in the treatment of neonatal coarctations and pulmonary artery stenoses.
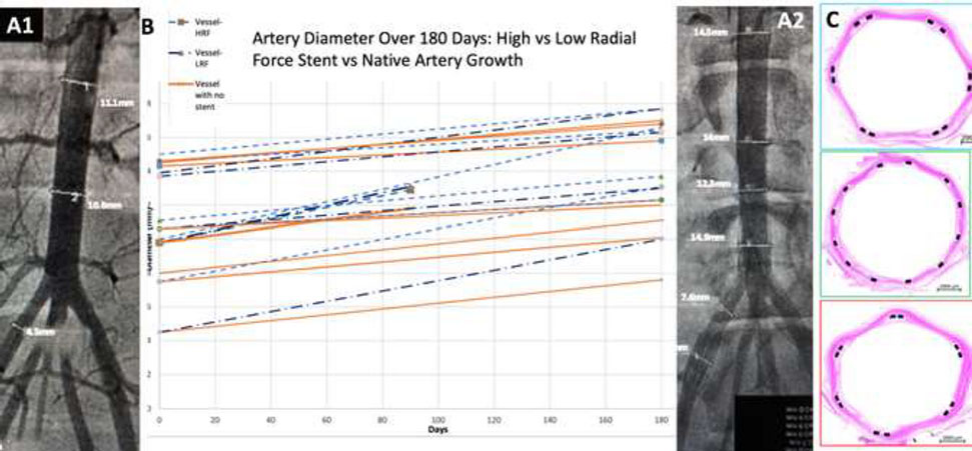


## 28

### Our Experience of Device Closure of Ostium Secundum Atrial Septal Defect (OS ASD) Under Transthoracic Echocardiography (TTE) and Fluoroscopy Guidance Without Using of Transesophageal Echocardiography (TEE)

Dr. M. Kalyanasundaram, Pediatric Cardiologist, GKNM Hospital, Coimbatore, India

**Abstract**

We report successful device closure of OS ASD in 58 patients (male 23, Female 35) with age ranged from 8 months to 75 years (median age 4 years) and weight ranged from 5.5 to 84 kg (median weight 14.6 kg) done between 15.10.19 and 20.3.20 with success rate of 95% and no major complications.

**Introduction:** Most of the centers use TEE and fluoroscopy and some centers use only TEE and TTE without fluoroscopy guidance to deploy the ASD device. We observed some patients complaining of pain and irritation in the throat and some had cough with blood-tinged sputum after ASD device closure was done with TEE guidance. We thought these complains were probably due to injury to the throat and esophagus by the TEE probe manipulation. Sometimes we encountered the problem of fully opened LA disc failure to align with the atrial septum while it was deployed under TEE guidance. In some of these cases, the device got aligned well with the septum once TEE probe was pulled up above the left atrium (LA) level. We hypothesized that compression of LA by the TEE probe was the reason for failure of the device to align with septum

**Methods and Materials:** 61 patients (male 24, Female 37) with age ranged from 8 months to 75 years (median age 4 years) and weight ranged from 5.5 to 84 kg (median weight 14.6 kg) were included in our study. All patients received loading doses of antiplatelets one day prior to the procedure. Procedure was done under general anesthesia with endotracheal intubation in 41 patients and under IV sedation in 20 patients. We used GE N95 echocardiography machine and fluoroscopy in all our patients to deploy the device.

**Discussion:** Out of 61 patients, device was deployed in 58 patients using TTE with success rate of 95%. We switched over to TEE in 3 patients, since septal margins were not clear in 2 patients before the procedure and in 1 patient after the deployment of device. Device closure of ASD only with TTE guidance was attempted to circumvent the post-procedure throat pain and irritation and also to avoid haemoptysis due to TEE probe injury. None of our 58 patients had these symptoms though it was difficult to get the throat pain history from small children. In our previous experience with TEE-guided deployment we encountered in some patients the problem of aligning the device with septum in spite of LA disc was fully opened. But the device aligned well with septum once the probe was pulled up above the LA level. This we attributed to the LA compression by the TEE probe preventing the fully expanded LA disc to align with the septum. None our patients in this study had such a deployment problem

**Conclusion:** Device closure of ASD under the guidance of TTE and fluoroscopy but without TEE is feasible, safe, and easy to align the device with septum in view of absence of compression of LA by TEE probe.

## 29

### A Systematic Approach to Pulmonary Valve Replacement in the Current Era

R. Allen Ligon MD^1^, Steve Bibevski MD, PhD^1^, Mark M. Ruzmetov MD, PhD^1^, Kak-Chen Chan MD^1^, Todd Roth MD^1^, Immanuel I. Turner^2^, Frank G. Scholl MD^1^, Larry A. Latson MD^1^

^1^Joe DiMaggio Children’s Hospital - Memorial Healthcare System, Hollywood, USA. ^2^Joe DiMaggio Children’s Hospital - Memorial Healthcare System, Hollywood, USA

**Abstract**

**Objective**: Pulmonary valve replacement (PVR) has become a common procedure in congenital heart disease patients and can be accomplished via surgical, transcatheter, or hybrid approaches. There are significant inherent advantages, especially from the patient perspective, to transcatheter PVR. However, transcatheter valves are only applicable in patients with a suitable landing zone. Further, there are inherent advantages to avoiding unnecessary use of cardiopulmonary bypass (CPB) in patients who require surgical cardiovascular interventions. Since 2017, we have adopted a standardized approach to PVR that includes all approaches to PVR. This includes off-pump hybrid PVR being preferred to standard on-bypass surgical PVR when the RVOT is too large for available transcatheter valves. The objective of this work is to review the results of our standardized institutional approach to PVR.

**Methods**: A retrospective review of all PVR cases and patient follow-up between February 2017 and February 2020. Patients underwent clinically indicated pre- and intraprocedure testing including cardiac MRI, transcatheter right ventricular outflow tract (RVOT) balloon sizing and coronary compression (CC) testing. Hybrid PVR entailed off-pump RVOT plication with subsequent percutaneous transcatheter PVR during the same procedure. We present our results of this systematic approach, aimed at minimizing the use of CPB.

**Results:** PVR was indicated in 55 pts. Primary transcatheter PVR was attempted in 37, a hybrid procedure was performed in 11, and on-pump surgical PVR was performed in 9. Median age at PVR was 27 years (6–65 years) and the most common diagnosis was tetralogy of Fallot (*n* = 39). Primary transcatheter PVR was successful in 35/37, with 2 converted to surgical PVR for stent/valve migration. Surgical valve implantation on cardiopulmonary bypass was utilized for positive CC testing (*n* = 4), stent/valve system migration (*n* = 2), or patient preference over the newer hybrid approach (*n* = 3). The hybrid group had a mean RVOT diameter of 34 mm (32–38 mm) by balloon-sizing pre-RVOT plication and then 25 mm (22–27 mm) post plication. All hybrid procedures were performed without CPB, except for one patient who underwent surgically-guided delivery of the transcatheter PVR due to change in RVOT anatomy/orientation pre and post plication. Median length of stay following procedure was 1 day for transcatheter PVR, 5 days for surgical, and 3 days for hybrid (*p* = 0.02). Median follow-up period was 1.5 years and there have been no reported episodes of endocarditis. There have been 3-valve reinterventions to date including one balloon valve dilation in the transcatheter PVR cohort, one balloon valve dilation, and a subsequent transcatheter valve-in-valve PVR in the surgical cohort. One hybrid patient expired (11 months post-index procedure) for multiple reasons, but including a coronary artery insult possibly occurring at the time of RVOT plication.

**Conclusion:** A systematic approach to PVR utilizing all approaches in pre-defined order of preference leads to consistent outcomes in a wide variety of anatomic configurations. Transcatheter PVR may be accomplished in the majority of patients with RVOT dysfunction. In patients with an unsuitably large outflow, utilizing a hybrid off-pump RVOT plication with subsequent transcatheter PVR is effective and avoids the need for cardiopulmonary bypass.

## 30

### Animal Testing of an HDBraid PDA Device for Low Birth Weight Infants

Daniel Levi

UCLA, Los Angeles, USA

**Abstract**

**Background**: Use of a microbraiding technology has been used to produce the *WEB*, a neurovascular aneurysm treatment recently approved by FDA. Novel, patented HDBraid technology has been used to produce the *LOBO* (Okami Medical, Aliso Viejo), an FDA-cleared peripheral occlusion device. HDBraid technology has also been used to produce a device design specifically for closure of PDAs in preterm infants using a microcatheter for device delivery.

**Methods**: Two-disk PDA devices were manufactured from 144 wires using the previously described and proprietary braiding technology and traditional shape setting. The device has two discs (4 mm and 7 mm) designed to elongate in a tubular fetal-type PDA (Figure 1). The PDA device is compatible with most commercially available 0.027″ microcatheters. The ductus of three newborn piglets was recannulized using balloon angioplasty with a 5 or 6 mm Tyshak Mini balloon (*NuMed*, Hopkinton, NY) in order to create 3 mm PDAs. The PDAs were occluded in both antegrade and retrograde fashion with the PDA device using ECHO and fluoroscopic guidance (Figure 2). Angiograms and ECHOs were performed to document occlusion and devices were examined postmortem as well. The ability to deliver, visualize, and release the device was assessed as well as time to complete angiographic occlusion.

**Results**: PDAs were crossed and successfully balloon open in three piglets weighing 1.9, 2.4, and 2.8 kg. After angioplasty, the PDA dimensions ranged from a diameter of 2.8–4.5 mm with lengths ranging from 5.7–7.4 mm. All devices were delivered and released without difficulty. The proximal and distal markers on the devices were easily visualized by fluoroscopy and the device was also able to be imaged by ECHO. Two PDAs were occluded antegrade and one retrograde. In one animal, the device was recaptured prior to release and redeployed. All PDAs were completely occluded within 5 min of device delivery without obstruction to the left pulmonary artery or descending aorta. In two of three animals, occlusion was immediate. Figure 2 demonstrates the angiographic and ultrasound appearance of the PDAs before and after occlusion. On postmortem exam, all three devices were in excellent position with an obvious layer of fibrin covering the surface of the braided nitinol (Figure 3).

**Conclusion**: Initial animal testing of the new microcatheter-based PDA device justifies further investigation of this device as a possible new device for occlusion of tubular PDAs in low birth weight infants.
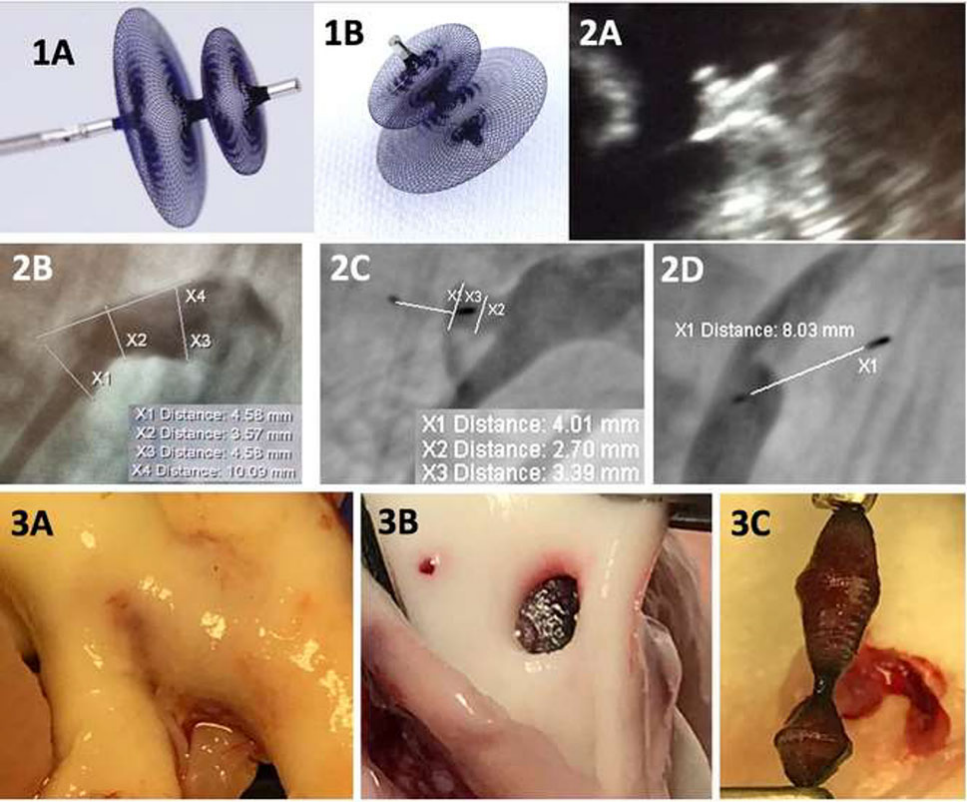


## 31

### Use of the Penumbra Embolization System for Vascular Occlusion in Patients with Congenital Heart Disease: A Single-Center Experience

Emily C. Kish, Renelle S. George, John S. Lozier, Martin L Bocks

Rainbow Babies & Children’s Hospital, Cleveland, USA

**Abstract**

**Background:** Occlusion of vascular anomalies is often necessary in patients with congenital heart disease (CHD). These anomalies can cause a variety of symptoms, including hypoxemia from right-to-left shunting, pulmonary overcirculation, angina from coronary insufficiency, and hemoptysis. These vessels are often tortuous, have acute take-offs and can be difficult to occlude using larger occlusion devices. The Penumbra Embolization System (PES) offers several advantages over other devices, including the ability to occlude tortuous or difficult to access vessels, resheath and repack coils, low-profile delivery catheters, and reduced need for numerous coils for particularly voluminous or long collaterals. We report our experience using PES for vascular occlusion in patients with CHD.

**Methods:** We retrospectively reviewed cases in which Penumbra coils were used for transcatheter vascular embolization at our institution between July 2018 and March 2020. Demographics, diagnoses, and catheterization data were analyzed.

**Results:** Eleven patients underwent occlusion of 17 vessels in 12 procedures during the study period. The median age was 10.5 years (0.9–58.0 years) with a median weight of 33.5 kg (8.1–98.0 kg). Seven patients had single ventricle physiology. There were six aortopulmonary collaterals, seven venovenous collaterals, two coronary artery fistulas (CAF), and two bronchial artery collaterals. Of the 12 cases during the study period, vascular occlusion was the only intervention performed in eight. PES products were the only devices used in 16/17 embolized vessels. The most common device combination involved anchoring Ruby coils or PODs followed by packing coils in 10 vessels. A Ruby coil or POD alone was sufficient for embolization in four vessels. Two vessels were embolized using packing coils only. In one vessel, a packing coil was used following placement of an Amplatzer™ Vascular Plug 4. The size of the Ruby or POD anchor coil ranged from 2 mm by 1 cm to 6 mm by 50 cm. The packing coils ranged from 5 cm to 60 cm in length. Two coils or fewer were used to achieve desired results in 13 vessels (76%).

All coils were successfully delivered through the Lantern microcatheter. Post-release angiograms demonstrated complete occlusion in 15 vessels, with near complete occlusion in two. There were no procedural complications. Follow-up angiography was not performed in any patients except for the patient with CAF, which was recanalized at six-month follow-up. The patient returned to the cath laboratory for successful occlusion using an additional POD followed by a packing coil.

**Conclusion:** We report the successful embolization of multiple vascular anomalies using various products from the PES. Complete embolization was achieved in the majority of cases, with no associated adverse events. The patient with recanalized CAF had non-densely packed coils. We now densely opacify and repack the Penumbra coils to avoid this outcome. Long-term follow-up may be needed to evaluate vessel patency and assess for recanalization. The PES effectively occludes large and high-flow vessels without the need to pack a large number of coils or use of a large device/delivery system. The microcatheter delivery system enables occlusion of difficult to access or tortuous vessels.

## 32

### My Nightmare Case in Cath Lab; Fit The Slip

Amjad Mahmood^1^, Khurram Akhtar^2^

^1^AFIC@NIHD, Rawalpindi, Pakistan. ^2^AFIC&NIHD, Rawalpindi, Pakistan

**Abstract**

A 12*40 mm balloon-mounted peripheral stent was deployed at RVOT to rescue severely stenosed infundibulum in a lady of 18 years with TOF who had failing RV extreme desaturation and declared unfit for surgical correction. During final deployment, the stent embolized into LPA. The fully expanded stent was successfully dragged and redeployed with the help of balloon inflation within the stent. After successful redeployment of the same stent, RVOT was adequately expanded and the patient saturated well to avoid further hypercyanotic spells.
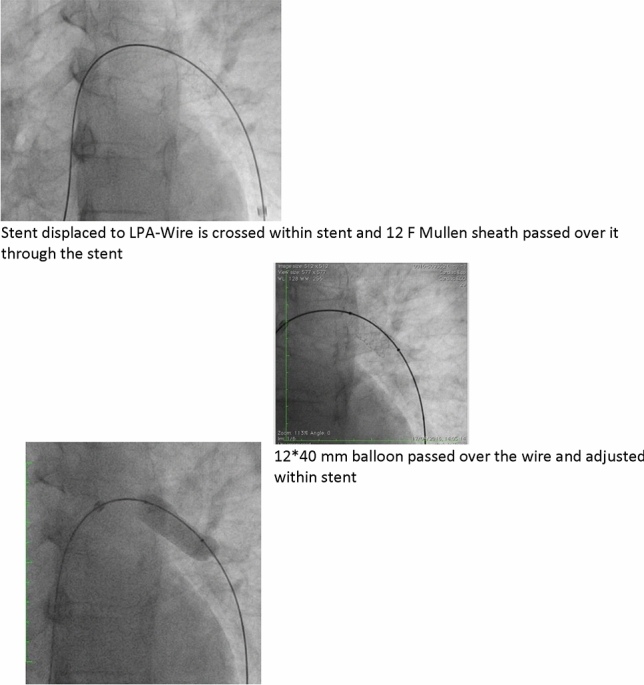

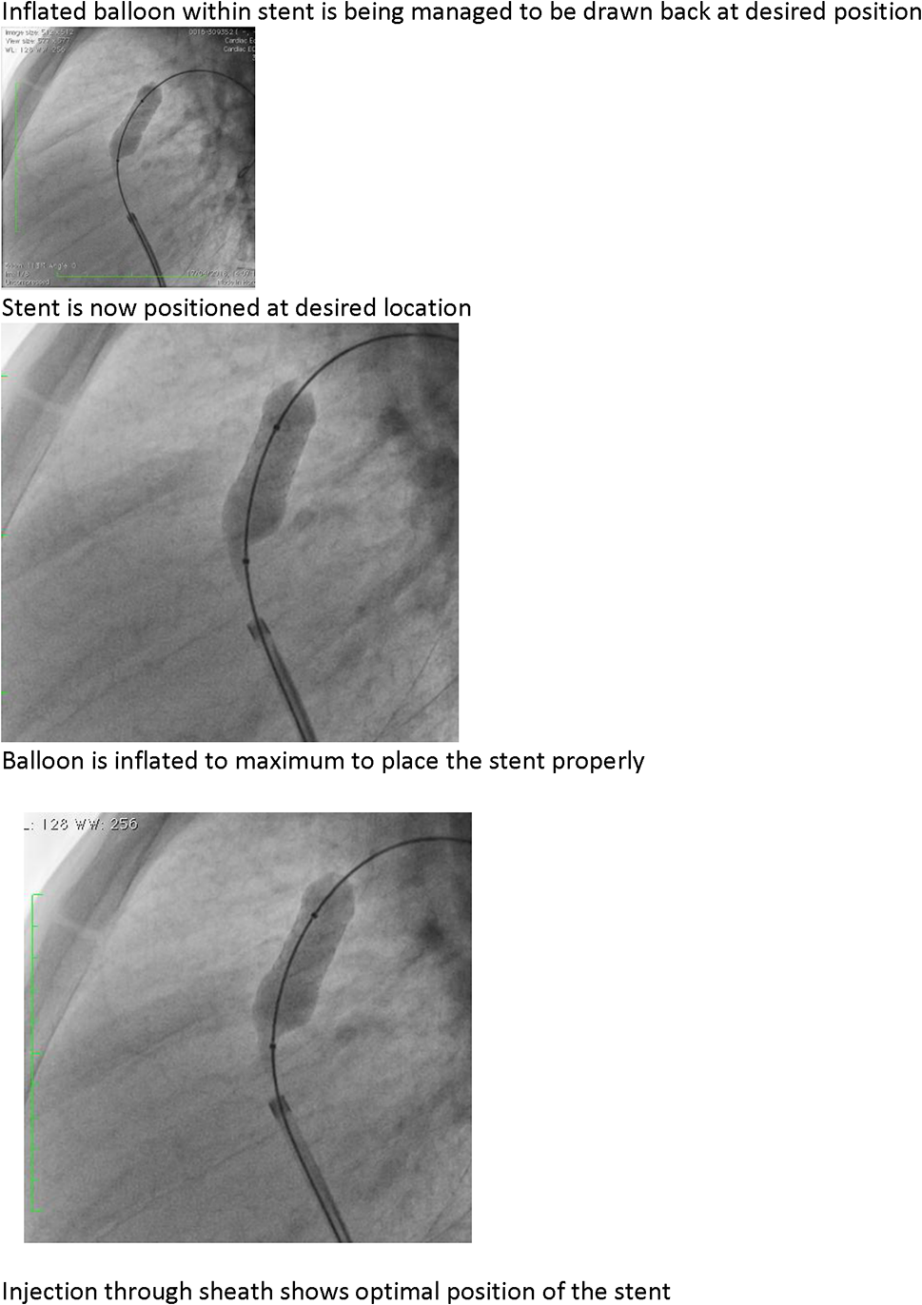

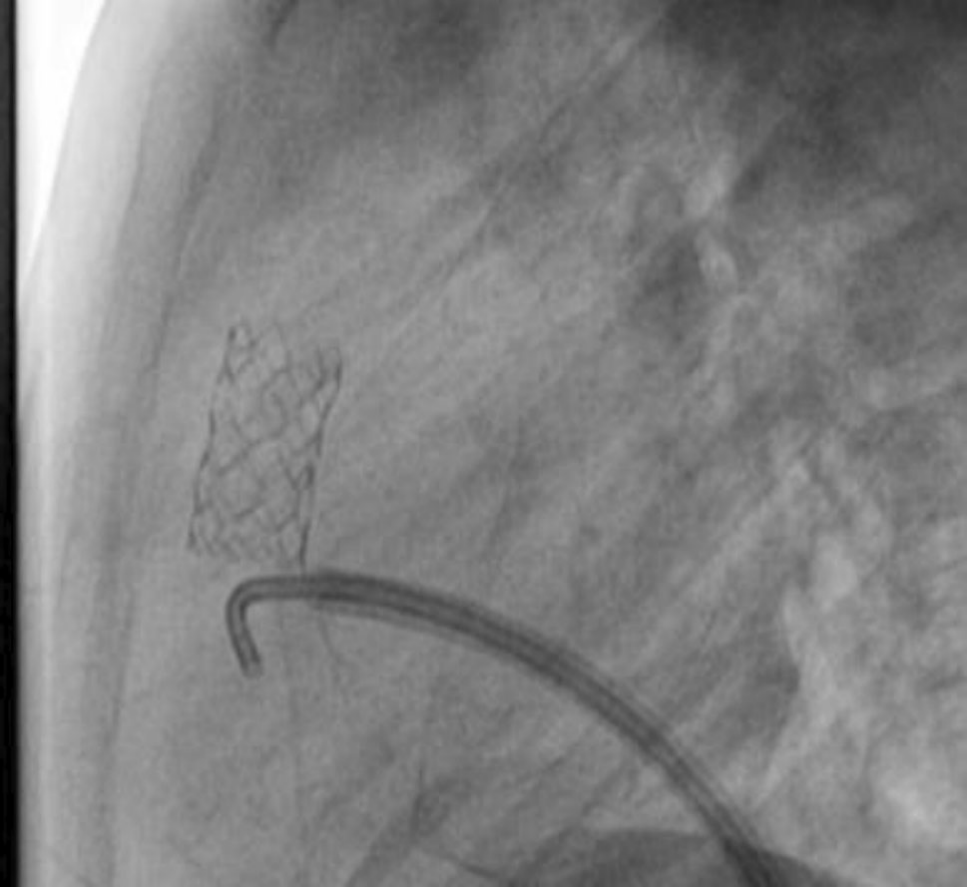


Check angiogram shows final position of stent

## 33

### Five-Year Outcomes from the Harmony Transcatheter Pulmonary Valve Early Feasibility Study

John Cheatham^1^, Matt Gillespie^2^, Lee Benson^3^, Shicheng Weng^4^, Lisa Bergersen^5^

^1^Nationwide Children’s Hospital, Columbus, OH, USA. ^2^Children’s Hospital of Philadelphia, Philadelphia, PA, USA. ^3^The Hospital for Sick Children, Toronto, ON, Canada. ^4^Medtronic, Mounds View, MN, USA. ^5^Boston Children’s Hospital, Boston, MA, USA

**Abstract**

**Background:** Patients with native or patched right ventricular outflow tracts (RVOTs) and severe pulmonary regurgitation (PR) comprise a large portion of the congenital heart disease population, for which there are no currently approved transcatheter treatments available. The Harmony Early Feasibility Study is the first FDA-approved study evaluating transcatheter pulmonary valve implantation in this population.

**Methods:** This non-randomized, prospective study evaluated the Harmony Transcatheter Pulmonary Valve (HTPV) device in the native RVOT. Candidates underwent detailed anatomic eligibility screening and were enrolled across three sites in the U.S. and Canada. The primary objective was to obtain in vivo data to confirm assumptions on loading conditions for future in vitro frame evaluations. Procedural feasibility, safety, and HTPV performance were assessed, and planned clinical and echocardiographic follow-up extends to 5 years.

**Results:** From 66 patients enrolled in the feasibility study, 21 were catheterized, and 20 underwent HTPV implantation. As previously published, 17 patients completed 3-year follow-up, with no late stent fractures or valve migration, and no reports of thrombosis, or endocarditis through 3 years. CT evaluation in sixteen patients showed anatomic incorporation of the valve, and tissue ingrowth that did not affect hemodynamic performance with the mean right ventricular outflow tract gradient remaining stable at 15.7 ± 5.5 mmHg. At 3-year follow-up, one patient (6.3%) had mild PR with the remaining patients having none/trace regurgitation (vs. 95% with severe PR at baseline). One patient (6.3%) had mild paravalvular leak (PVL) at 3 years while the remaining patients had none/trace PVL. At the time of presentation, 5-year results will be available.

**Conclusions:** Longer-term follow-up data for TPV implantation in the native outflow tract were limited, and the 5-year outcomes from the Harmony Early Feasibility Study represent the longest follow-up of any TPV in the native outflow tract.

## 34

### Patent Ductus Arteriosus Stenting for All Ductal-Dependent Cyanotic Infants: Waning Use of Blalock–Taussig Shunts

Howaida El-Said^1^, Kanishka Ratnayaka^1^, Stephen Nageotte^2^, John W Moore^1^, Peter Guyon^1^, Krishna Bhandari^1^, Rachel Weber^3^, Jesse W Lee^4^, Hyeri You^1^, Danica Griffin^1^, Rohit P Rao^5^, John J Nigro^1^

^1^UCSD, San Diego, USA. ^2^St. Louis Children’s Hospital, St Louis, USA. ^3^Rady Children’s Hospital, San Diego, USA. ^4^The Children’s Hospital of San Antonio, san Antonio, USA. ^5^UCSD, san, USA

**Abstract**

**Background:** Ductal-dependent cyanotic newborns require a secure source of pulmonary blood flow. There has been a recent migration to selective ductal (PDA) stenting for some of these children.

**Objective:** Universal (non-selective) ductal stenting for all infants with ductal-dependent pulmonary blood flow (DDPBF) is controversial. We examine outcomes from a single center with this practice change.

**Methods:** We compare outcomes of all DDPBF infants (2013–2019) in the following treatment eras: Era 1 (selective PDA stenting; 2013–2017) or Era 2 (universal PDA stenting; 2018–2019).

**Results:** Eighty-three patients [Blalock–Taussig Shunt (BTS), n = 41; PDA stent, n = 42] met inclusion criteria. In Era 1, most received BTS [62% (41/66)]. In Era 2, all received PDA stents [100% (17/17)]. There were no demographic differences between eras. There were no differences in mortality, treatment failures, complications, or reinterventions between eras. Post-procedure length of stay was shorter in Era 2 (8 vs 22 days, *p* < 0.05). There were less surgical revisions for PDA stent patients (2% vs 20%, *p* < 0.05). Post-procedure recovery surrogate endpoints favored Era 2 and PDA stenting. Additional analysis revealed PDA stent (compared to BTS) patients had shorter overall (18 vs 43 days, *p* < 0.05) and post-procedure (11 vs 29 days, *p* < 0.05) length of stay and more symmetric branch pulmonary arteries (0.9 vs 0.7, *p* < 0.05) at subsequent surgery.

**Conclusions:** Universal PDA stenting for all ductal-dependent cyanotic newborns is safe and effective, and may have lower morbidity and require fewer resources than selective PDA stenting.

## 35

### Neonatal PDA Stenting for Ductal-Dependent Pulmonary Blood Flow Considerations: Anticoagulation Therapy, Unplanned Reintervention Rates, and Pulmonary Artery Growth

Yousef Arar, V. Vivian Dimas, Surendranath R. Veeram Reddy, Thomas M. Zellers, Carrie Herbert

University of Texas Southwestern/Children’s Medical Center, Dallas, USA

**Abstract**

**Background**: Patent ductus arteriosus (PDA) stenting represents a viable alternative to surgical shunts (modified Blalock–Taussig shunt; MBTS) for treatment of neonates with critical congenital heart disease in need of stable postnatal pulmonary circulation without the need for cardiopulmonary bypass or sternotomy. Our institution’s anticoagulation protocols post-PDA stenting consist of either aspirin (ASA) alone or ASA with enoxaparin. Our primary aim was to evaluate our anticoagulation protocols to help clarify which were most helpful in preventing unplanned reinterventions and promote pulmonary artery (PA) growth, especially as it relates to the growth of small jailed PAs.

**Methods**: Retrospective chart review was performed on neonates who underwent PDA stenting for ductal-dependent pulmonary blood flow at our institution between 2014 to 2020. We specifically compared anticoagulation therapy as it relates to reintervention rates and PA growth for jailed versus non-jailed PAs. Bare-metal coronary, non-drug-eluting stents were used for all patients. Anticoagulation therapy was based on interventionalist preference with no institutional consensus on practice.

**Results**: A total of 76 patients underwent successful PDA stenting with demographic and outcome variables outlined in Table 1. There were no procedure-related deaths. Figure 1 summarizes total rate of unplanned reinterventions and PA growth analysis. A total of 34 patients were excluded from the study for the following: lack of pre-surgical PA imaging (n = 12), death (n = 9), current status of PDA stent (n = 6), status post heart transplant (n = 5), or transfer of care (n = 2) for a total of 42 patients. A comparison of anticoagulation therapy for jailed versus non-jailed PAs was analyzed for rates of unplanned reinterventions and PA growth (Table 2). Anticoagulation strategy using antiplatelet therapy (ASA) alone versus antiplatelet plus anticoagulation therapy (ASA plus enoxaparin) did not significantly change the rate of reintervention for jailed versus non-jailed PAs. In addition, there was no statistically significant benefit in PA growth for jailed PAs when using ASA plus enoxaparin versus ASA alone (Table 3). Patients who received ASA plus enoxaparin for documented thrombus post-procedure had the highest rate of PDA stent reintervention. A case example of each group is summarized in Figure 2. No bleeding or acute thrombotic events were noted in this patient cohort after procedural success.

**Conclusions**: Neonatal PDA stenting is a safe and feasible alternative to MBTS for ductal-dependent pulmonary blood flow. Anticoagulation strategies using ASA therapy alone were adequate with no appreciable benefit from adding enoxaparin as it relates to rate of unplanned reintervention or PA growth for jailed/non-jailed PAs. Future studies are needed to assess rates of ASA resistance in the neonatal PDA stenting for ductal-dependent pulmonary blood flow cohort.
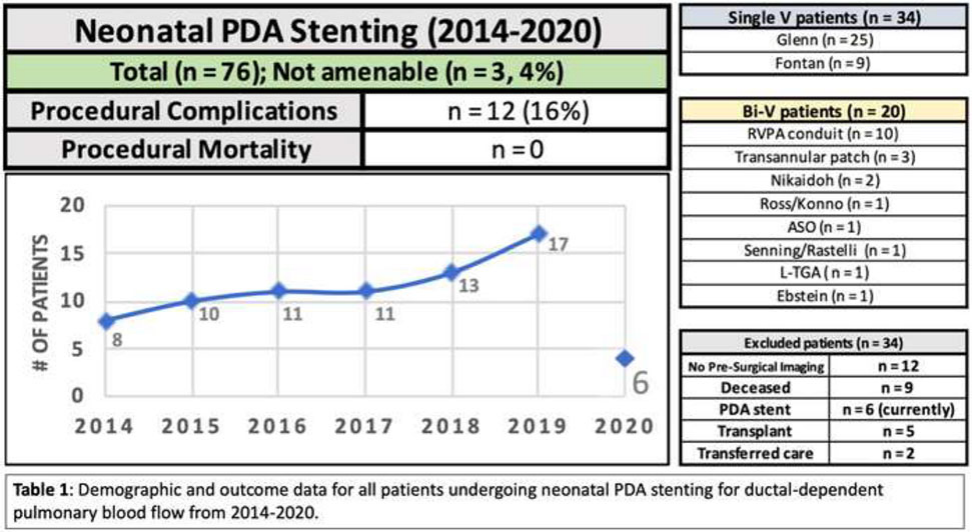

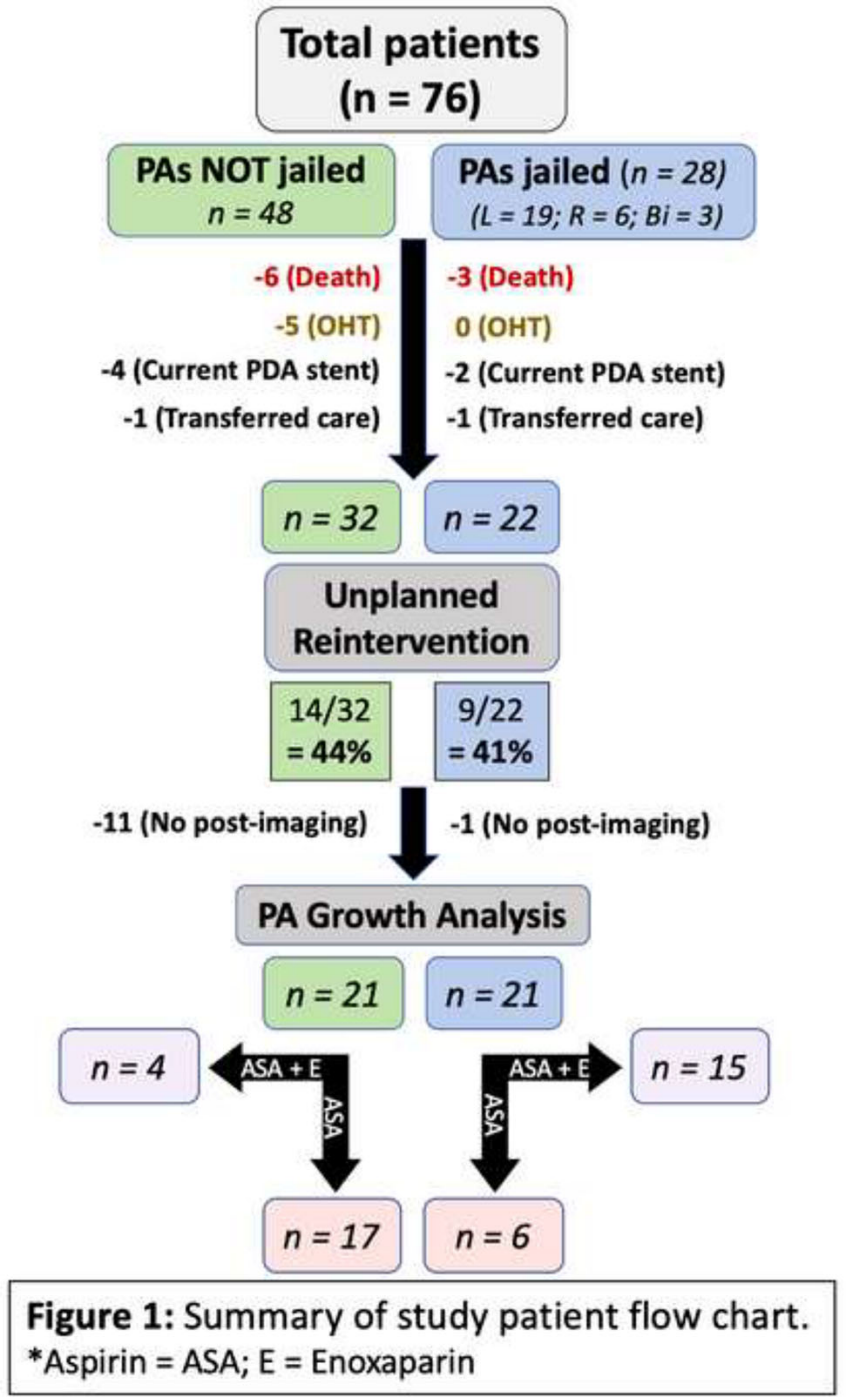

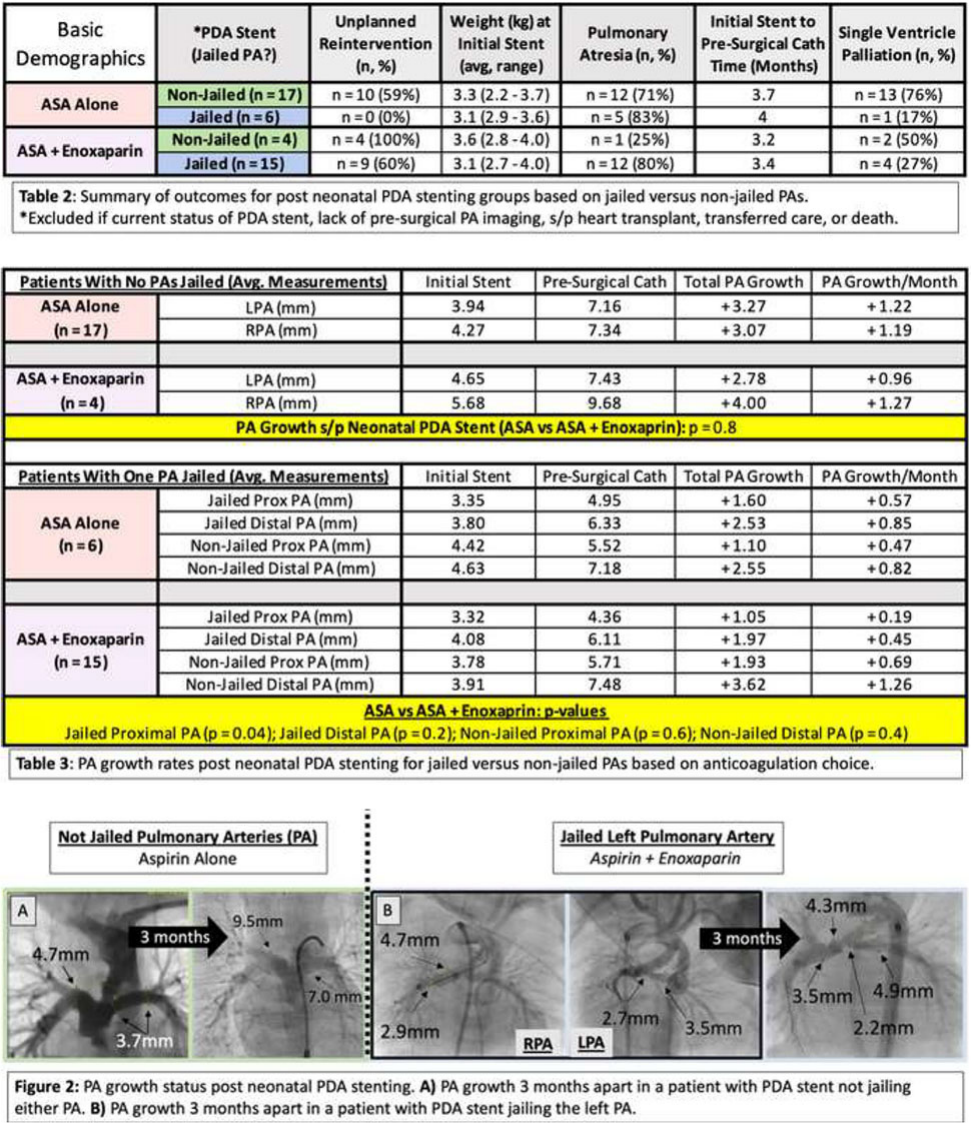


## 36

### Six-Month Outcomes of the Harmony Transcatheter Pulmonary Valve Pivotal Trial

Doff McElhinney^1^, Dan Levi^2^, Matthew Gillespie^3^, Thomas Jones^4^, Robert Gray^5^, Tomoyuki Fujita^6^, Jeremy Asnes^7^, Shicheng Weng^8^, John Cheatham^9^

^1^Stanford University, Stanford, CA, USA. ^2^UCLA, Los Angeles, CA, USA. ^3^Children’s Hospital of Philadelphia, Philadelphia, PA, USA. ^4^Seattle Children’s Hospital, Seattle, WA, USA. ^5^University of Utah, Salt Lake City, UT, USA. ^6^National Cerebral and Cardiovascular Center, Osaka, Japan. ^7^Yale University, New Haven, CT, USA. ^8^Medtronic, Mounds View, MN, USA. ^9^Nationwide Children’s Hospital, Columbus, OH, USA

**Abstract**

**Background:** The self-expanding Harmony transcatheter pulmonary valve (TPV) is designed to relieve chronic pulmonary regurgitation (PR) in the native or large patched right ventricular outflow tract (RVOT). Safety and efficacy of the Harmony TPV were reported previously as part of the Harmony Early Feasibility Study. The Harmony TPV Pivotal Trial continues this investigation and includes two valve sizes—TPV22 and the larger TPV25—as well as a small subset of modified TPV 25 Harmony implants, which included a redesigned frame to improve performance.

**Methods:** The Harmony Pivotal Trial is a prospective, single-arm study evaluating Harmony device performance and procedural safety in patients indicated for surgical pulmonary valve or conduit placement. Patients were enrolled at 12 participating centers in the U.S., Japan, and Canada. Candidates included patients with severe PR on echocardiography or PR fraction ≥ 30% by magnetic resonance imaging. The primary safety endpoint was freedom from procedure- or device-related mortality at 30 days, and the primary efficacy endpoint was acceptable hemodynamic performance (mean RVOT gradient ≤ 40 mmHg on echocardiography and PR fraction < 20% on magnetic resonance imaging or PR < moderate by echo) and no interventions on the Harmony TPV post implant through 6 months.

**Results:** Forty patients underwent TPVR with either the TPV22 (*n* = 21) or TPV25 (*n* = 19), followed by 10 additional patients receiving a modified TPV25, for a total of 50 study patients. The mean age was 28.7 ± 11.3 years. Thirty-one patients (62%) were male, 46 (92%) had an original diagnosis of tetralogy of Fallot, and the median number of prior open-heart surgeries was 1 (Q1, Q3: 1, 2). Primary safety and performance outcomes were previously reported for the TPV22 and TPV25 cohort, along with 30-day performance of the modified TPV25. The current presentation will include complete data for 6-month endpoints in all patients, including those who received the modified TPV 25.

**Conclusions:** The growing body of data on the Harmony TPV show its early promise as a less-invasive alternative to surgical pulmonary valve replacement in selected patients with severe PR in a native or patched RVOT. The full 6-month results represent the longest follow-up to date using the modified TPV25 valve design.

## 37

### Novel Minimal Radiation Multimodality Approach for Percutaneous Pulmonary Valve Implantation

Jenny Zablah^1,2^, Salvador Rodriguez^1^, Ryan Leahy^1^, Gareth Morgan^1,2^

^1^Children’s Hospital Colorado, Aurora, USA. ^2^University of Colorado Hospital, Aurora, USA

**Abstract**

**Objective:** To evaluate the impact of multimodality imaging technology application during percutaneous pulmonary valve implantation (PPVI) for radiation reduction without increasing the rate of adverse events.

**Introduction:** Among all the percutaneous procedures, PPVI could potentially have one of the highest radiation exposures for patients. Different protocol modifications have been implemented to address this problem (i.e., improvements in guidance system, delivery system, valve design, post-implantation evaluation) and the effectiveness of individual modifications have been proven, however, the effect of a combined approach has not been reported.

**Methods:** We performed a retrospective chart review of 76 patients who underwent PPVI between January 2018 and December 2019. Patients were classified in traditional protocol, using routine angiography and/or 3D rotational angiography (3DRA); and multimodality protocol, according to the application of new technology (such as VesselNavigator for guidance, Long DrySeal Sheath for valve delivery, and Intracardiac Echocardiography for valve evaluation after implantation). Radiation metrics, procedural time, and clinical outcomes were compared between groups.

**Results:** When the traditional protocol group was compared with the multimodality protocol group, a significant reduction was described for total fluoroscopy time (31.6 min vs. 26.2 min), dose of contrast per kilogram (1.8 mL/Kg vs. 0.9 mL/Kg), DAP/kg (26.6 µGy·m^2^/kg vs. 19.9 µGy·m^2^/kg), and Air Kerma (194 mGy vs. 99.9 mGy). Interestingly, a reduction for procedure time was noted (140 min vs. 116.5 min), but no statistical difference was described. No differences were found for clinical outcomes or the presence of complications between groups.

**Conclusions**: The combination of novel technology in PPVI caused a significant reduction in radiation metrics without increasing the complication rate in our population.

## 38

### Coronary Compression Testing by Balloon Interrogation During Pulmonary Valve Implantation; Room for Doubt?

Gareth Morgan^1,2^, Salvador Rodriguez^1^, Ryan Leahy^1^, Jess Randall^1^, Jenny Zablah^1,2^

^1^Children’s Hospital Colorado, Aurora, USA. ^2^University of Colorado Hospital, Aurora, USA

**Abstract**

**Objective**: To evaluate the ability of balloon coronary compression testing during percutaneous pulmonary valve implantation (PPVI) to predict the final spatial relationship between the implanted valve and the coronary arteries, and to explore how variations in RVOT substrates, equipment, and technique can influence this.

**Background:** Despite the widespread use of the “balloon coronary test” as the preferable method to rule out the risk of coronary compression (CC), this event has been described after PPVI where coronary balloon test suggested no risk or low risk, calling into question the accuracy of this assessment. No methods to evaluate the false-positive rate have been established, however, some possible factors that affect the assessment have been proposed.

**Methods:** We performed a retrospective chart review of 84 patients who underwent PPVI between January 2018 and December 2019 and selected 35 patients whose archived imaging was suitable to perform analysis of the “balloon coronary test.” We focused on the spatial disparity between the RVOT position with the testing balloon inflated and the eventual implanted valve position to classify the test as inaccurate or accurate. Baseline and procedural characteristics were evaluated to determine their association as predictors of test accuracy.

**Results:** 36.1% of cases were classified as having an inaccurate coronary balloon test. Among the baseline characteristics, the RVOT substrate was found as a significant predictor for an inaccurate test. Related to this characteristic, the balloon used and the smaller sizes of the implanted valves were found associated with the test inaccuracy. The rest of the baseline an procedural characteristics did not show association with the event of interest.

**Conclusions:** Based on our findings balloon coronary testing is not an accurate method of predicting the RVOT position after valve implantation. This may translate to a high rate of false positives for coronary compression in the wider PPVI population.

## 39

### Percutaneous Common Carotid Artery Access for Cardiac Interventions in Infants Does not Acutely Change Cerebral Perfusion

Subhrajit Lahiri, Athar Qureshi, Henri Justino, Emad Mossad

Baylor College of Medicine, Houston, USA

**Abstract**

**Background:** Pediatric cardiac interventions via percutaneous common carotid artery access have been shown to be safe and effective. However, the impact of placement of a sheath in the carotid artery for interventions on changes in cerebral perfusion is unknown. In this study we use cerebral near-infrared spectroscopy (cNIRS), which has been established as a marker of cerebral perfusion, to analyze the effects of carotid artery access for cardiac interventions on cerebral perfusion.

**Methods:** This is a retrospective chart review done at a tertiary care center for all pediatric patients that underwent percutaneous cardiac catheterization via carotid artery access from January 2009 to January 2020. All patients who had ipsilateral cNIRS monitored on the side of carotid artery access were included. Patients with no ipsilateral cNIRS data or partial cNIRS data were excluded. Primary outcome was the change in cNIRS with carotid artery access. Mean cNIRS for 15 min before obtaining access was compared to mean cNIRS during the procedure and 15 min after removal of the carotid artery sheath. We hypothesized that there is significant drop in cNIRS on the side of carotid artery access.

**Results:** There were 48 catheterizations in the study period where carotid artery was accessed. Out of those, 22 catheterizations with complete data were included in the study. Thirteen (50%) were males. Median age was 23 (IQR 7,79) days. The indications for carotid artery access were PDA stent implantation (*n* = 13,48%), aortic valvuloplasty (*n* = 5,22%), balloon angioplasty of coarctation of aorta (*n* = 2,10%), and renal artery angioplasty (*n* = 1,4%). In 16 patients (72%), the left common carotid artery was accessed. The median weight of the patients was 3.3 kg (IQR 2.8, 2.9). The most common sheath size used was 4F in 16 patients (72%). The average cNIRS prior to the procedure was 67 ± 15 (mean ± SD), during the procedure was 68 ± 20 (mean ± SD), and after removal of sheath was 68 ± 21 (mean ± SD). Paired t test of cerebral cNIRS before and during the procedure showed no significant change with carotid artery access (*p* = 0.08). Paired t test of means of cNIRS during and after procedure showed no significant change with removal of carotid artery sheath. No patient in the series had a documented neurologic deficit following the procedure.

**Conclusion:** Percutaneous common carotid artery access is not associated with a decrease in cNIRS on the side of the access suggesting there is no significant change in cerebral perfusion with carotid artery access.

## 40

### Ten-Year Outcomes in the Melody Transcatheter Pulmonary Valve U.S. Investigational Device Exemption Trial

Darren Berman^1,2^, Thomas Jones^3^, Doff McElhinney^4^, Julie Vincent^5^, William Hellenbrand^6^, John Cheatham^2^, Evan Zahn^7^, Danyal Khan^8^, John Rhodes^9^, Shicheng Weng^10^, Lisa Bergersen^11^

^1^The Ohio State University College of Medicine, Columbus, USA. ^2^Nationwide Children’s Hospital, Columbus, USA. ^3^Seattle Children’s Hospital, Seattle, USA. ^4^Stanford University, Stanford, USA. ^5^Columbia University Medical Center, New York, USA. ^6^Yale School of Medicine, New Haven, USA. ^7^Cedars-Sinai Heart Institute, Los Angeles, USA. ^8^Miami Children’s Hospital, Miami, USA. ^9^Medical University of South Carolina, Charleston, USA. ^10^Medtronic, Mounds View, USA. ^11^Children’s Hospital, Boston, USA

**Abstract**

**Background:** The Melody valve was the first transcatheter heart valve made commercially available. It received CE mark in 2006 and initiated enrollment in a U.S. Investigational Device Exemption (IDE) clinical trial in 2007. Early IDE trial results showed safety and efficacy in treating dysfunctional right ventricular outflow tract (RVOT) conduits, and follow-up to 7 years demonstrated durable clinical and hemodynamic outcomes. Final ten-year data from the Melody IDE trial were first presented in spring 2020.

**Methods:** One hundred and seventy-one patients with RVOT conduit obstruction and/or regurgitation were enrolled in the trial. Patients were followed out to ten years. Valve dysfunction was assessed as freedom from reoperation, catheter reintervention, or hemodynamic dysfunction (defined as moderate or severe pulmonary regurgitation and/or mean RVOT gradient > 40 mmHg). Long-term safety outcomes included device-related serious adverse events, stent fracture, catheter reintervention, surgical conduit replacement, and death.

**Results:** Among the 171 enrolled patients, a total of 150 patients underwent transcatheter valve implantation (TPVI) with the Melody valve. Median age at TPVI was 19 years (range 7–53). Fifty-one percent of enrolled patients had tetralogy of Fallot. As previously published, mean RVOT Doppler gradient was 17 mmHg at discharge. Five-year freedom from reintervention and freedom from explant were 76 ± 4% and 92 ± 3%, respectively. Thirty-two patients underwent RVOT reintervention at a median 4.5 years of follow-up. The majority of reinterventions were for RVOT obstruction (*n* = 27), while two patients underwent reintervention for endocarditis, and one patient for RV dysfunction. The current encore presentation will review 10-year outcomes as previously presented at SCAI 2020.

**Conclusions:** Since it was first introduced, the Melody TPV has been an adaptive tool for less-invasive conduit reintervention and a life-changing innovation for congenital heart disease (CHD) patients who face multiple reoperations through adolescence and beyond. Ten-year outcomes from the Melody IDE trial demonstrate long-term efficacy and durability of TPVI in dysfunctional RVOT conduits. These clinical trial results provide a strong foundation for future technologies in transcatheter therapies and evidence-based medicine for the CHD patient population.

## 41

### Orthogonal Angles in Fluoroscopy: Mathematical Formulas

John Lozier, Emily Kish, Renelle George, Martin Bocks

UH Rainbow Babies & Children’s Hospital, Cleveland, USA

**Abstract**

Optimal angiographic imaging angles are essential in transcatheter interventions. However, achieving ideal views of complex or unique cardiovascular structures can be challenging. We sought to develop a tool to assist in obtaining optimal imaging angles using the mathematics of vectors. For a given vector v_1_, there exists a plane defined as the set of all vectors that are perpendicular to v_1_. This concept may be useful in cardiac angiography: if the “down-the-barrel” viewing angle of a vessel or structure is known, then many orthogonal camera angles could be derived to provide optimal “profile” views. Further, if two profile viewing angles of a structure are known, then the down-the-barrel angle can be derived.

**Methods:** Two mathematical formulas were developed to solve for orthogonal angles to a known camera position in a standard fluoroscopy system. In formula 1, a user entered the cranial/caudal and LAO/RAO angles of the down-the-barrel view, and one user-defined angle for a profile view (cranial/caudal or LAO/RAO); the formula then solved the second profile angle, with the result that the profile view was orthogonal to the down-the-barrel view. Formula 2 derived the down-the-barrel camera angles when two profile views were known. The formulas were converted to a mobile computing app.

To test the formulas, an X-ray phantom was designed and 3D printed.

**Results:** The X-ray phantom’s disc and cylinder were perpendicular by fluoroscopy. Moving either camera by single degree increments resulted in significant changes in the fluoroscopic appearance of the phantom.

One hundred trials were performed to assess formula 1. Mathematically, formula 1 generated high-quality solutions, with a mean angle of 90.01 degrees (range 89.52 to 90.42 degrees) between the down-the-barrel view and the calculated profile angles. Fluoroscopically, 99 of the calculated profile angles appeared perpendicular, while one appeared to be one degree off perpendicular (mean fluoroscopic angle 0.01 ± 0.1 degrees off perpendicular).

Ten trials were performed to assess formula 2, which also generated high-quality mathematical solutions. The mean angle between the known profile angles and the calculated down-the-barrel angles was 89.93 degrees (range 89.45 to 90.53 degrees). Fluoroscopically, nine of the calculated down-the-barrel angles appeared perpendicular, while one appeared to be one degree off perpendicular (mean fluoroscopic angle 0.1 ± 0.3 degrees off perpendicular).

**Discussion:** We have successfully developed an easy-to-use tool to assist in obtaining optimal fluoroscopic images using the mathematics of vectors. Formula 1 can calculate a nearly unlimited number of orthogonal profile viewing angles when a down-the-barrel angle is known. Formula 2 derives the down-the-barrel angle when two profile views are known. This simple tool is likely to be useful in a number of interventional cases, including pulmonary artery rehabilitation, pulmonary vein stenosis, assessment of atrial septal occluders, and other complex interventions. The formulas could be made widely available via mobile computing app or integrated into cath lab fluoroscopy software. Ultimately this tool may optimize cardiac angiographic imaging and reduce unnecessary radiation and contrast exposure.

## 42

### Expandable Polyurethane Stent Valve, implanted by catheter, in pediatric and adults patients: Results from Physical, Hydrodynamic, Animal, and Ultrastructural Studies

Miguel Maluf

São Paulo Federal University, São Paulo, Brazil. Synthetic Heart Institute, São Paulo, Brazil

**Abstract**

**Background:** Patients with pediatric prostheses suffer from mismatch and early calcification, which causes a high number of reoperations.

**Methods:** Expandable Polyurethane Stent Valve—EPSV is composed by a flexible polyurethane (PU) cusps is grown on the top of an expandable cobalt-chrome alloy stent, including the formation of three leaflets. Physical, Hydrodynamic, and Animal studies were performed following ISO 5840-3, 2015.

**Results: Physical tests.** Result of study of surface scanning of pre and post crimp stent showed no structural modification of the PU. Hydrodynamic test showed a pressure gradient oscillation between 5 and 20 mm, in basal or stress conditions, respectively. Experimental studies. Sheep were subjected to 3D echo-Doppler study, in 6th follow-up months, which showed satisfactory hemodynamic performance, with low transvalvular gradient (*M* = 6.60 mmHg).

Ultrastructural Study: Six stents were explanted after 20 days to 21 months of follow-up to Ultrastructural analysis. All of which revealed no presence of calcium growth and prostheses structure was intact.

**Conclusions:** Expandable stent valve and PU no calcification are good expectations for pediatric use.

## 43

### Critical Aortic Stenosis: Different Approaches for a Very Ill Newborn

Gehan Attia Alsawah, Hala Almarsafawy, Mona Hafez

Professor of pediatric cardiology Manoura University, Mansoura, Egypt

**Abstract**

**Background:** Critical aortic valve stenosis (CAS) represents an emergency, and immediate treatment is mandatory. CAS is a ductus-dependent CHD because the open ductus arteriosus supplies systemic circulation. Balloon aortic valvuloplasty (BAV) is now the first therapeutic option. To dilate the critically stenotic aortic valve, there are different approaches for best outcome.

**Objective:** Here, we present our mid-term results in Pediatric Cardiology Unit, Children Hospital, Mansoura University in of different approaches of percutaneous balloon aortic valve valvuloplasty (BAV) in cases of critical aortic stenosis (CAS) in the period from 2005 to 2018 to assess the safety and efficacy of transcatheter intervention for critical aortic stenosis.

**Method:** Between April 2005 and June 2018, all consecutive patients with CAS treated with balloon valvuloplasty in our hospital were analyzed retrospectively. Patients were followed up from 18 months to 13 years (mean 100.8 months [8.4 years]) by clinical examination and echocardiography. RESULT: Sixty-seven consecutive patients were analyzed. Their gestational age was 37.53 ± 1.37 weeks. Eleven patients (16.4%) were preterm newborn. Postnatal age 8.5  ±  7.6 days, range 4–35 days. Weight 2.75 ± 0.43 kg. 58.5% was male and 41.5% was female. Fifty-seven of patient (85.07%) received PGE1 infusion before the procedure to maintain the ductal patency in a dose of 0.05 to 0.1 µg/kg/min. Balloon valvuloplasty was accomplished in 65 (97.01%) of 67 interventions. The procedural approaches, success, early outcome, complication rates, mid-term results, and aortic regurgitation were retrospectively studied. Pre-dilatation by Brio coronary balloon was used in 19 patients (28.35%) followed by TAYSHAk^®^ mini balloon. TAYSHAk^®^ mini balloon was used from the start in 48 patients (71.6%) with balloon annulus ratio 0.85. In nineteen patients, antegrade approach was used through the patent foramen ovale, in 21 patients, trans-femoral retrograde approach was used, whereas, in 25 patients, trans-carotid approach was used. Peak-to-peak pressure gradient across the valve fell from (85.4 ± 14.0) to (24.7 ± 14.0) (P < 0.001). Left ventricular pressure fell from (101.4 ± 16.0) mmHg to (45.5 ± 10.5) mmHg (*P* < 0.001). Two patients had very difficult aortic valve cannulation with the wire then referred for surgical valvotomy. Two patients lost follow-up. Echocardiography on follow-up revealed a mean trans-aortic systolic gradient of < 30 mmHg, none to grade I aortic. On follow-up 6 children (9.2%) required a second balloon dilatation with good results.

**Conclusion:** Balloon aortic valvuloplasty is relatively safe and effective in neonatal CAS. There is a different approach for this procedure. Antegrade procedure is safe and requires shorter procedure duration. It may be associated with a lower morbidity and mortality than surgical treatment

## 44

### Pulmonary Artery Intervention Following Transcatheter Pulmonary Valve (TPV) Implantation can be Performed Safely Without Compromising TPV Function and Integrity

Sharib Gaffar^1,2^, Sanjay P. Sinha^2,1,3^, Michael R. Recto^2^

^1^University of California Irvine, Irvine, USA. ^2^CHOC Children’s Hospital of Orange County, Orange, USA. ^3^University of California Los Angeles, Los Angeles, USA

**Abstract**

**Background:** Transcatheter pulmonary valve (TPV) implantation has become accepted practice for patients of all ages following FDA approval of the Melody valve (Medtronic, Minneapolis MN) in 2010. Limited information is available regarding TPV leaflet integrity following transcatheter intervention through previously implanted TPV. Little is known about the effect of interventions that require repeated crossing of the TPV on valve leaflet integrity. This case series describes 6 patients who underwent pulmonary artery stent implantation and pulmonary balloon angioplasty through previously implanted Melody and Sapien (Edwards Lifesciences, Irvine CA) TPV.

**Methods:** Six patients (3 females and 3 males), with mean age 15 years (range 10–20), mean weight 54 kg (range 39–66), 2 with tetralogy of Fallot (TOF), 3 with variant TOF (absent pulmonary valve, pulmonary atresia, pulmonary atresia/ventricular septal defect with major aortopulmonary collateral vessels), and one patient with truncus arteriosus type 1 underwent TPV implantation. Five patients underwent Melody valve implantation and one patient had a Sapien TPV implanted. All patients previously underwent surgical complete repair with development of severe pulmonary valve insufficiency and right ventricular enlargement necessitating TPV implantation. Following TPV implantation, 6 patients had evidence of either distal main pulmonary artery or branch pulmonary artery stenosis. Two of the patients, one requiring distal main pulmonary artery stenting and another requiring left pulmonary artery stent redilation, underwent a second procedure 10.2 and 7.4 months after TPV implantation, considered early in our experience. The other 4 patients underwent pulmonary artery intervention during the same catheterization procedure after first implanting the TPV. Both angioplasty balloons and stents were advanced over an Amplatz Super Stiff wire (Boston Scientific, Marlborough MA) or through a long transseptal sheath to minimize the number of times required to cross the TPV. All six patients underwent pulmonary artery angioplasty with Atlas Gold (Bard, New Providence, NJ), Vida (Bard), Powerflex (Cardinal Health, Dublin OH), or Opta Pro (Cardinal) balloons. Four of the six patients underwent stent implantation into distal main pulmonary artery stenosis (one patient) or right or left pulmonary artery (3 patients) with Palmaz Genesis (Cardinal) or Max LD (Medtronic) stents. All six patients underwent at least 2 interventions (median 2.5, range 2–4) through the TPV. All six patients underwent successful distal main pulmonary artery or branch pulmonary artery balloon angioplasty and/or stent implantation without complications. Post-intervention angiography showed competence of the TPV with normal leaflet function and no evidence of pulmonary valve insufficiency, perivalvular leak, or valve migration. All patients had post-catheterization transthoracic echocardiography demonstrating TPV leaflet integrity without evidence of insufficiency. Postcatheter course was uneventful for all patients.

**Conclusion:** In our series, multiple interventions through previously implanted TPV including balloon angioplasty and stenting of the distal main pulmonary artery and branch pulmonary arteries was safe, effective, and caused no damage to the previously implanted TPV.

## 45

### Percutaneous Transluminal Angioplasty for Central Venous Stenosis in Hemodialysis Patients—A Case Series

Roshan Valentine, Muthusubramanian Rajasekaran, Reddi Prasad Yadavalli, Rohan Augustine

Manipal Hospital, Bangalore, India

**Abstract**

Thoracic central venous system comprises deep venous system continuing from the head, neck, upper limb to the heart through the thoracic inlet corresponding to the level of T1 vertebra. These include the superior vena cava (SVC), Brachiocephalic vein (BCV), subclavian vein (SCV), intrathoracic segment of Internal Jugular Vein (IJV), and suprahepatic Inferior Vena Cava (IVC). Stenoses of these vessels are not infrequent with most common cause being multiple punctures while cannulating the IJV or SCV. The other causes include turbulent flow secondary to AV shunts, thrombin sheath formation within the indwelling catheters. Clinically they present with ipsilateral arm or face swelling, increased venous pressure during hemodialysis(HD) and failed access. Rather than the conventional treatment options, endovascular management remains as a standalone promising treatment option. In this pictorial exhibit, we present 4 patients with central venous stenosis who were referred to Interventional Radiology department for endovascular management.

**Case Series:**

**Table:** 

Age/sex	Presenting symptom	Level of stenosis	Endovascular management
58/female	SVC syndrome	SVC + right BCV	Successful
74/male	Left arm swelling	SVC + left BCV	Successful
65/male	Increased HD venous pressure	SCV + left BCV	Successful
44/female	Left arm swelling with increased HD venous pressure	Left BCV (stent stenosis)	Successful

**Conclusion:** Thoracic central venous obstruction is fairly common in patients with central venous catheters and high-flow pressure within these catheters is considered its Achilles Heel. PTA is an essential component in the dialysis access armamentarium and has become the de-facto choice in case of central venous stenosis

## 46

### Transcatheter Closure of Perimembranous VSD and PDA Using Amplatzer AVP II Devices in a 10-Month-Old/6 kg Weight Infant

Adrian Sanchez Flores^1^, Bryan Barrera Ruvalcaba^2^

^1^Hospital General Tijuana, Tijuana, Mexico. ^2^IMSS Clínica 1, Tijuana, Mexico

**Abstract**

A 10-month-old female patient with a medical history of Down syndrome, at 1 month of age reported with tachypnea and diaphoresis during feedings. Assessed by paediatric cardiologist: reported physical examination an III/VI holosystolic murmur and a single and loud S2 sound, diagnosed with: 6 mm PM VSD, 5 mm ASD, small PDA and pulmonary hypertension (45 mmHg). In class III Ross classification, stared treatment with double diuretic. Assessed monthly for clinical and hemodynamic repercussion: with failure to thrive, throughout de following months with poor weight gain and pulmonary hypertension. Its decided at 10 months old to be taken to the cath lab, due to her failure to thrive and very low weight and to avoid open heart surgery to try to benefit her with a PDA closure due to hemodynamic repercussion: dilated left cavities and pulmonary hypertension, and clinical repercussion: weight under 1% percentile for her current age. In the procedure reported with moderate pulmonary hypertension with 55 mmHg (mean: 40 mmHg) of systolic pulmonary pressure: 20 mmHg under systemic pressure. The PDA (type C) was successfully closed through retrograde approach with an Amplatzer AVP II 6 mm device. A left ventricular angiography on four chambers view was made to investigate the VSD defect, which measured 8 mm. It was decided during the procedure for maximum benefit and to avoid open-heart surgery to close the VSD. The PM VSD was closed through a retrograde approach using a 5Fr Judkins right guiding catheter and a 0.035-inch guide wire to cross the VSD. Using the same guide catheter a 8 mm AVP II Amplatzer device was loaded and deployed successfully, only with angiography without eco guidance. Proper device position was confirmed with LV angiography, only noticing a minimal residual shunt and pulmonary pressure dropping 10 mmHg (mean: 40 mmHg). At her two-month post procedure check up: reported in Class I Ross classification, and 1.1 kg weight gain. Her transthoracic echocardiogram with the device in proper position with a 1.5 mm residual shunt generating a 55 mmHg gradient, LVDD: 28 mm (Z score: 1.1), and normal pulmonary pressure: 25 mmHg. An electrocardiogram reported in normal sinus rhythm. Using different devices such as the Amplatzer AVP II for transcatheter closure of perimembranous VSD defects is feasible and safe in small infants, with low weight, due to the fact that this devices can be deployed through smaller delivery catheters making it possible to close larger PM VSD defects, without causing rhythm disturbances or aortic/tricuspid regurgitation due to the softer material and design characteristics of this devices adapting well.
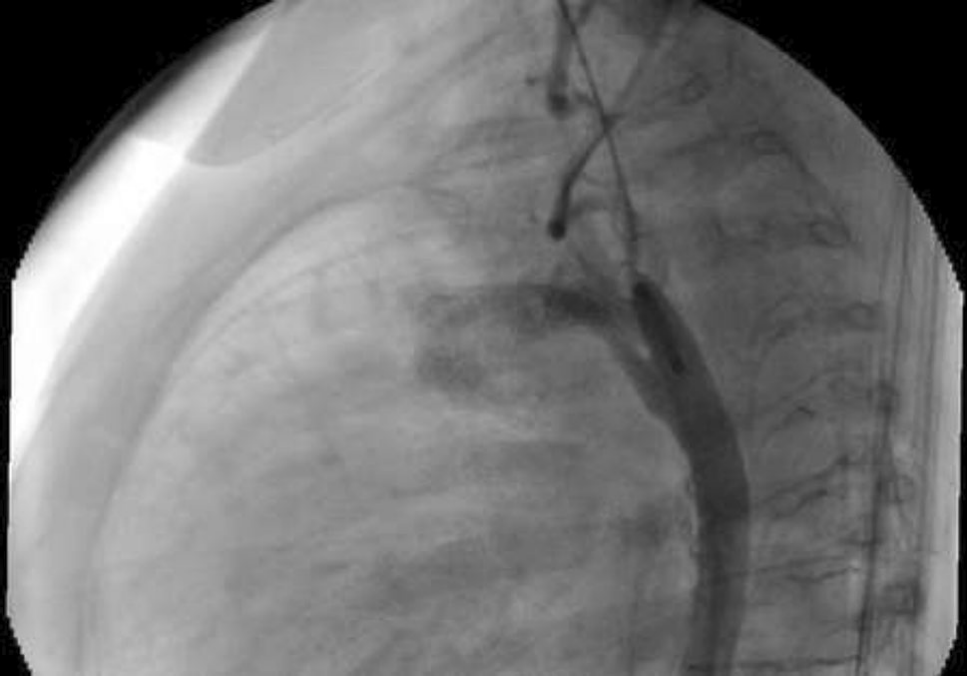

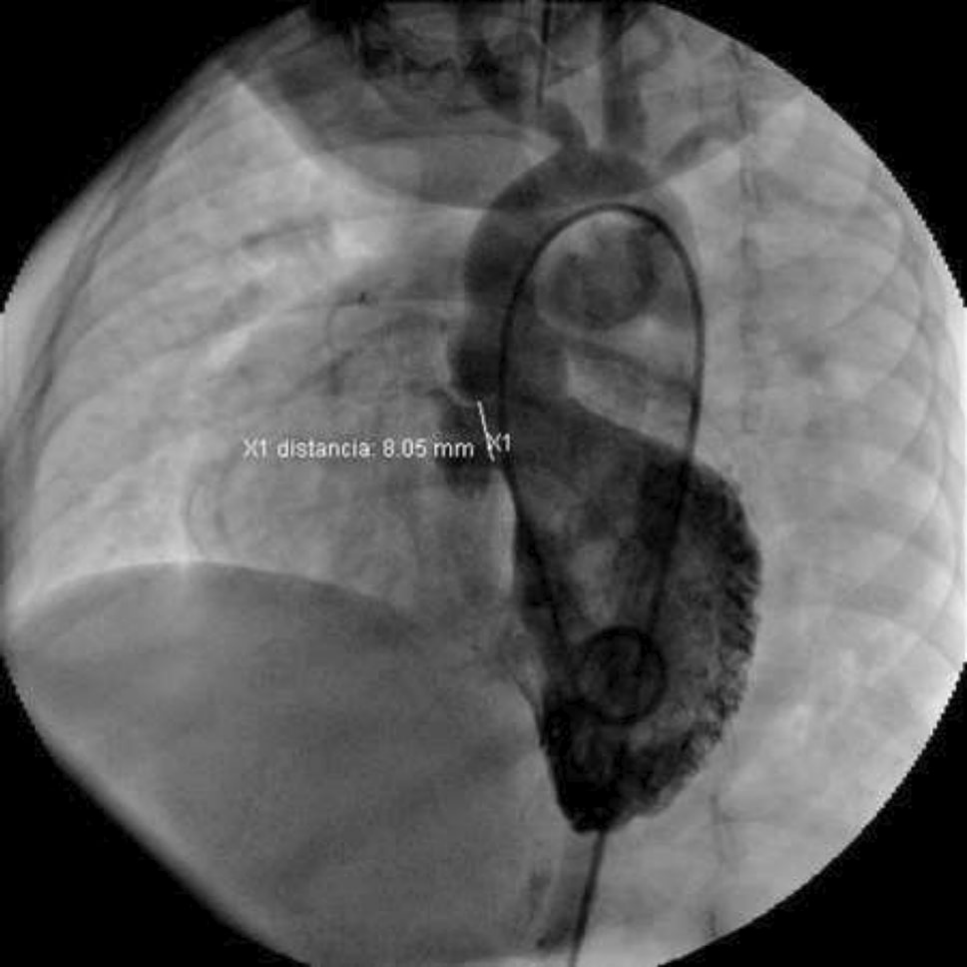

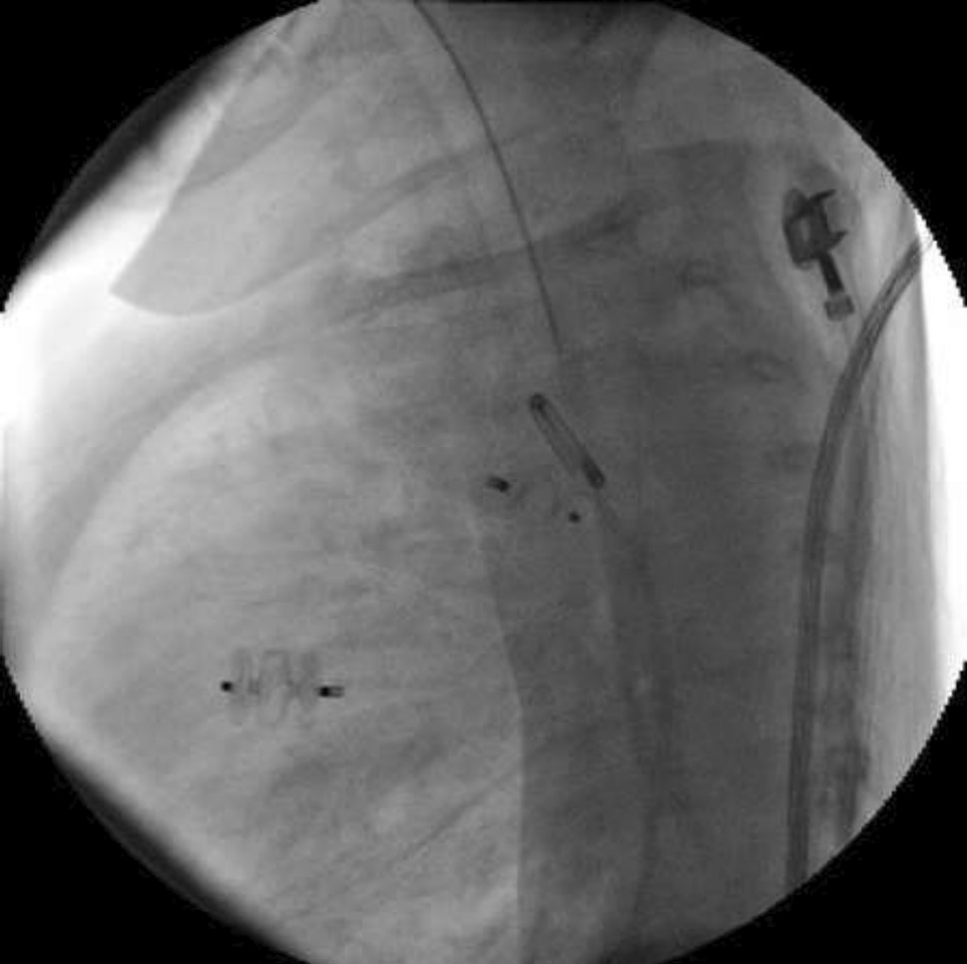


## 47

### Multicenter Pivotal Study of Alterra Adaptive Prestent and SAPIEN 3 Transcatheter Heart Valve in Congenital Pulmonic Valve Dysfunction

Evan Zahn^1^, Vivian Dimas^2^, Vasilis Babaliaros^3^, Dennis Kim^3^, D. Scott Lim^4^, Gareth Morgan^5^, Thomas Jones^6^, Aimee Armstrong^7^, Jamil Aboulhosn^8^, Vaikom Mahadevan^9^, Matthew Gillespie^10^, David Balzer^11^, Girish Shirali^12^, Anitha Parthiban^12^, Jonathan Leipsic^13^, Philipp Blanke^13^, Yanjun Chen^14^, Shabana Shahanavaz^11^

^1^The Smidt Heart Institute and the Department of Pediatrics, Cedars-Sinai Medical Center, Los Angeles, USA. ^2^Division of Cardiology, Department of Pediatrics, University of Texas Southwestern Medical Center, Children’s Health System of Texas, Dallas, USA. ^3^Division of Cardiology, Structural Heart and Valve Center, Emory University, Atlanta, USA. ^4^University of Virginia, Charlottesville, USA. ^5^Children’s Hospital, University of Colorado, Aurora, USA. ^6^Seattle Children’s Hospital, Seattle, USA. ^7^Nationwide Children’s Hospital, Columbus, USA. ^8^Ahmanson/UCLA Adult Congenital Heart Center, Los Angeles, USA. ^9^University of California, San Francisco, San Francisco, USA. ^10^Children’s Hospital of Philadelphia, Philadelphia, USA. ^11^Washington University in St. Louis School of Medicine, St. Louis, USA. ^12^Children’s Mercy Hospital, Kansas City, USA. ^13^St. Paul’s Hospital, Vancouver, Canada. ^14^Edwards Lifesciences, Irvine, USA

**Abstract**

Multicenter Pivotal Study of Alterra Adaptive Prestent and SAPIEN 3 Transcatheter Heart Valve in Congenital Pulmonic Valve Dysfunction.

**Background**: Interpatient variability in the size and morphology of right ventricular outflow tract (RVOT) can limit transcatheter pulmonary valve implantation (TPVI). A stent implanted into the RVOT can reduce RVOT size and provide a circular, stable landing zone for TPVI. This study evaluated the Alterra Adaptive Prestent with the SAPIEN 3 transcatheter heart valve (THV) in patients with dysfunctional RVOT/pulmonary valve (PV) in the US.

**Methods**: This single-arm, prospective, multicenter study was designed to evaluate up to 60 patients with dysfunctional RVOT/PV, moderate or severe pulmonary regurgitation (PR), weight ≥ 20 kg, and RVOT/PV proximal and distal landing zone diameter ≥ 27 mm and ≤ 38 mm, and minimum of 35 mm from contractile tissue to lowest pulmonary artery takeoff. The primary endpoint was THV dysfunction at 6 months, defined as RVOT/PV intervention, ≥ moderate PR, and mean RVOT/PV gradient ≥ 35 mmHg. Secondary endpoint outcomes included improvement in total PR at 30 days and acute device success, defined as a single Alterra Adaptive Prestent implanted in the desired location, a single THV implanted in the desired location within the Alterra Adaptive Prestent, RV-PA peak-to-peak gradient < 35 mmHg post implantation, < moderate PR by discharge, and free of explant at 24 h post implantation. Additional effectiveness outcomes included improvement in tricuspid regurgitation (TR) for patients with ≥ mild TR at baseline. Freedom from adjudicated adverse events was determined with Kaplan–Meier estimates. Descriptive statistics were used for other endpoints.

**Results**: Enrolled patients (N = 60) were a mean age of 29.5 years; 50% were ≤ 21 years old. The cohort was 56.7% male with a mean weight of 73.1 kg, and 92% New York Heart Association class I/II. Fifty-nine patients had the Alterra and SAPIEN 3 THV devices concomitantly implanted; 1 patient had a staged procedure. All devices were implanted in the intended location. Acute device success was 93.3%: a single Alterra Adaptive Prestent implanted in the desired location (100%), a single THV implanted in the desired location within the Alterra Adaptive Prestent (96.7%), RV-PA peak-to-peak gradient < 35 mmHg post implantation (98.3%), < moderate PR by discharge (98.3%), free of explant at 24 h post implantation (100%). At 30 days, < moderate total PR was 98.3%; 1.7% (*n* = 1) had moderate PR (Fig. 1). At 30 days, TR was ≤ mild in 83.4% of patients. No death, pulmonary embolism, RVOT reintervention, endocarditis, clinically relevant arrythmia, or coronary artery compression were reported at 30 days. Alterra frame fracture was 1.7% at 30 days and 10% at 6 months. Frame fracture was not associated with a change in valve function and no patient has required an intervention.

The 6-month primary endpoint data will be available at the time of the presentation.

**Conclusion**: Early results demonstrate the safety and effectiveness of the Alterra Adaptive Prestent and SAPIEN 3 THV in patients with dysfunctional RVOT/PV.

**Figure:**
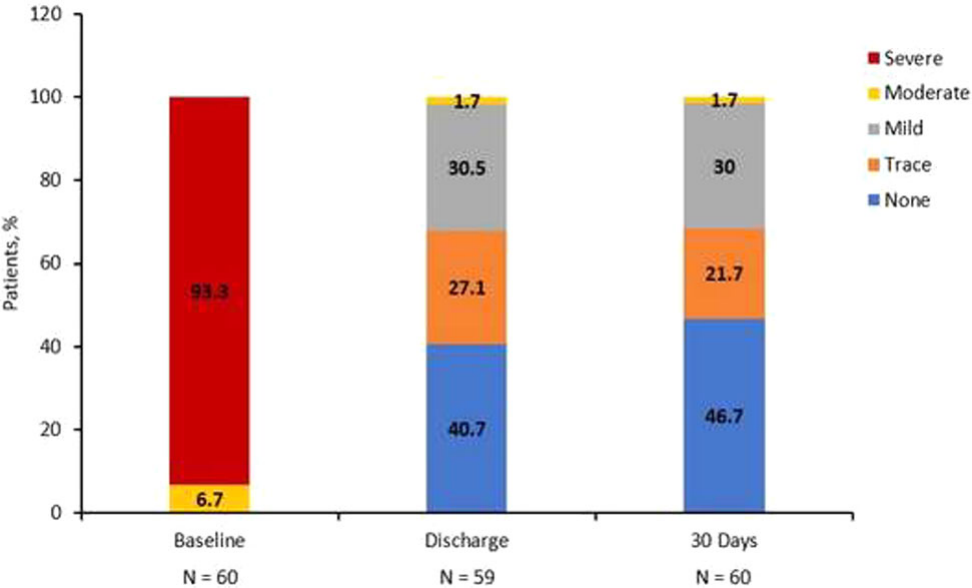
**Fig. 1** Total pulmonic regurgitation to 30 days

## 48

### Perventricular VSD Closure in a Jehovah Witness Boy

Silvia Cecilia Britton Robles^1^, Jesús Manuel Yañez Sánchez^1^, Gloria Cristina Aguilar Arredondo^1,2^, Otoniel Salinas Lozano^1^, Abraham Patricio Garza Castro^1^, Rosemberg Albores Figueroa^1,2^, Juan Alberto Quintanilla Guitérrez^1^, Mario Castro Medina^3,1^

^1^ITESM, Monterrey, Mexico. ^2^UMAE, Monterrey, Mexico. ^3^Children’s Hospital of Pittsburgh, Pittsburgh, USA

**Abstract**

A 7-month-old boy, Jehovah Witness, diagnosed with a 11-mm ventricular septal defect complicated with pulmonary hypertension was admitted to the ER with decompensated congestive heart failure, oxygen saturation of 92% at room air, bilateral crackles, and evident shortness of breath. The patient was stabilized with supplementary oxygen, diuretics, and inotropic support. Pulmonary infection was ruled out. Seven days after the admission, the patient was subject to a perventricular VSD closure. The procedure was guided by transesophageal echocardiogram, placing a 12-mm Amplatzer^®^ VSD device to fully close the defect, without cardiopulmonary bypass support. The procedure terminated successfully, extubating the patient in the OR, without the need for blood transfusion with no other immediate complications. The patient was admitted to the PICU with minimal respiratory and inotropic support, with a stable and favorable evolution. No arrythmias nor bleeding presented within the first days after the procedure. Hemodynamic improvement was evidenced with echocardiogram which showed decreased tricuspid regurgitation and decreased pulmonary pressure. The patient was discharged eight days post surgery with minimal diuretics, aspirin, and phosphodiesterase inhibitors dose. This case is relevant, because it shows how religious beliefs can change the management of congenital heart defects. The surgical closure of the defect with a patch would be the first approach to resolve the defect, but the need for blood transfusion limited this possibility. The placement of a VSD device occluder with a hybrid approach was a suitable alternative considering the location of the defect and the borders for the correct implant of a metallic device by a perventricular puncture without the need of blood transfusions nor cardiopulmonary bypass; giving the family a relative low-risk procedure with physical, psychological, and spiritual safeness.

## 49

### Initial Experience Assessing Feasibility and Safety of Conscious Sedation in Patients Undergoing Transcatheter Pulmonic Valve Implantation

Rajeev Anchan^1^, Diane Weibeler^1^, AbdulRahman Dia^1^, Joseph Venturini^1^, Daniel Gruenstein^2^, Sajid Shahul^3^, Rohan Kalathiya^1^, John Blair^1^, Stephanie Besser^1^, Jonathan Paul^1^, Sandeep Nathan^1^, Atman Shah^1^

^1^University of Chicago Medical Center, Section of Cardiology, Chicago, USA. ^2^University of Chicago Medical Center, Department of Pediatrics, Chicago, USA. ^3^University of Chicago Medical Center, Department of Anesthesia and Critical Care, Chicago, USA

**Abstract**

**Background:** Transcatheter pulmonic valve implantation (TPVI) is a minimally invasive procedure that treats mechanical dysfunction of the pulmonic valve (PV) or right ventricular to pulmonary artery conduit. TPVI procedures have traditionally been performed under general anesthesia (GA). However, similar to other structural interventions, conscious sedation (CS) has emerged as an alternative to GA. The purpose of this study was to assess the efficacy and safety of CS-TPVI.

**Materials and Methods:** We performed a retrospective review of 19 consecutive patients undergoing TPVI with CS or GA. The primary efficacy endpoint was procedural success, defined as residual gradient < 20 mmHg and freedom from reoperation within 30 days. Secondary efficacy and safety outcomes included the procedure duration, 90-day all-cause mortality and 30-day hospital readmission rates, procedure characteristics, and periprocedural complications.

**Results:** Nineteen patients underwent TPVI, eight of which received CS. The procedure success rate was not statistically different between GA and CS cohorts; one case in the GA-TPVI group had an elevated transvalvular gradient of 24 mmHg. The CS-TPVI 90-day all-cause mortality and 30-day hospital readmission rates were 0%. Median in-room procedure time was less with CS than GA (132 min (92–170, IQR) versus 196 min (179–234, IQR), *p* = 0.008). There were no differences in procedure or valve delivery durations, contrast use, radiation exposure, length of stay, or periprocedural complications between groups.

**Conclusions:** In a single-center experience, the use of CS for TPVI was safe, less-invasive, and more efficient than GA. Our study demonstrates the importance of a multidisciplinary approach in complex congenital heart disease patients and underscores the need for larger studies investigating CS-TPVI.
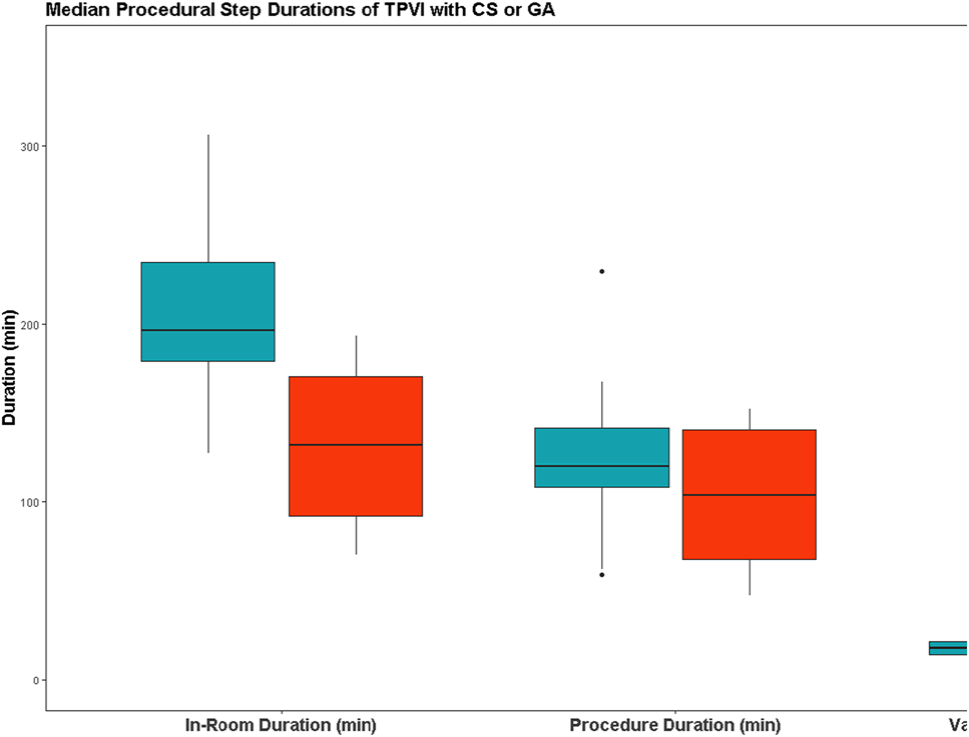


## 50

### Body Mass Index and Age are Associated with Ventricular End-diastolic Pressure in Adults with a Fontan Circulation

Mary Howell MD, William Anderson MS, Jorge Alegria MD, Joseph Paolillo MD, Matthew C. Schwartz MD

Carolinas Medical Center, Charlotte, USA

**Abstract**

**Introduction:** Systemic ventricular end-diastolic pressure (SVEDP) is an important hemodynamic variable in adult patients with a Fontan circulation. Risk factors that are associated with an elevated SVEDP have not been clearly identified in this patient population.

**Methods:** All patients > 18 yo with a Fontan circulation who underwent cardiac catheterization at our center between 1/08 and 3/19 were included. Relevant patient variables were extracted, and statistical analysis was performed to identify patient variables that were associated with SVEDP measured at catheterization.

**Results:** Forty-two patients were included with a mean weight of 72.4 kg ± 19.5, mean age of 26.3 years ± 8.5, and a mean BMI of 25.6 kg/m^2^ ± 6.2 at the time of catheterization. Ten (23.8%) patients had a systemic right ventricle (RV) and 5 (11.9%) had undergone a Norwood operation. The mean SVEDP was 11.1 mmHg ± 3.5 and mean pulmonary artery pressure was 16 mmHg ± 4.0. On univariate analysis, SVEDP was positively associated with BMI (*p* < 0.01), age > 25 yo (*p* = 0.04), taking diuretics at time of catheterization (*p* < 0.01), taking sildenafil at time of catheterization (*p* < 0.01), symptoms of heart failure(*p* < 0.01), systemic ventricular systolic pressure (*p* = 0.03), pulmonary artery mean pressure (*p* < 0.01), and systemic vascular resistance (*p* < 0.01). The SVEDP was negatively associated with aortic saturation (*p* < 0.01). On multivariate analysis, SVEDP was positively associated with age > 25 yo at catheterization (*p* < 0.01), BMI (*p* = 0.04), and symptoms of heart failure (*p* = 0.05).

**Conclusions:** In a cohort of adult patients with Fontan circulation undergoing cardiac catheterization, SVEDP was positively associated with BMI, age > 25 yo, and the presence of heart failure symptoms on multivariate analysis. Maintaining a healthy BMI is important in adult patients who have undergone Fontan operation and may offer hemodynamic benefit and protect against heart failure symptoms.

## 51

### Integrating Digital Media to Enhance The Patient–Parent Cath Lab Experience

Kristie Barras^1^, Hannah Reese^1^, Miguel Behrhorst^1^, Kiran Mallula^2^

^1^Children’s Hospital of New Orleans, New Orleans, USA. ^2^LSU Health, New Orleans, USA

**Abstract**

**Background:** As pediatric cath lab nurses, we explain every process in a step by step sequential manner, to patients and parents prior to proceeding with any procedure. We are compassionate and caring towards every child as if they were our own. However, families still tend to be apprehensive about entrusting their children to stranger providers and children also appear to be anxious prior to being escorted to strange cath lab environments with new provider faces. Two years ago, the Cardiovascular Surgical team implemented the EASE application for patients and families. This EASE (Electronic Access to surgical events) app is installed on a family member’s cell phone and allows any team member to send free text, photos, and video updates during and after the procedure. This improved the real-time communication between team members and families, and hence was very beneficial for enhancing the provider–patient trust relationship and reducing the anxiety associated with these procedures.

**Objective:** In May 2020, two state-of-the-art new cath labs opened doors to meet the growing demand of an ever increasing diverse Pediatric cardiac population. This was felt to be an opportune moment to introduce both new and prior families to the brand-new space. And, from the positive feedback received from the EASE app, we wanted to further enhance the family experience and ease the anxiety by showing them the cath lab environment as well as sharing the experience of undergoing anesthesia through a digital media presentation.

**Materials and Methods:** A virtual tour of a patient who will undergo a catheter procedure will be filmed from the time of check-into discharge and will mimic the actual day of the procedure. All staff will be introduced through a taped meet and greet session. This instructional video will be shot in English medium and posted on our Heart Center’s web page as well as the hospital’s affiliated web-based resources for all families to view. To cater to diverse populations with various socioeconomic backgrounds, a designated tablet will be supplied to all patients and families to view this introduction video upon admission as well. All families will be surveyed with a brief questionnaire about their experience with the digital media presentation.

**Results and Conclusions:** The survey will include families over a three month period and the data to showcase the positive effects of integrating digital media technology into the patient/family cath lab experience will be reported at the upcoming PICS-AICS session.

## 52

### Percutaneous Transcatheter Melody Valve-in-Valve Replacement in the Tricuspid VALVE Position—A Case Series

Kishore Raja^1^, Satinder Sandhu^2^

^1^Jackson Memorial Hospital, Miami, USA. ^2^University of Miami, Miami, USA

**Abstract**

**Objective:** We describe the short-term results for transcatheter tricuspid valve-in-valve implantation (TVIVI) using the Melody valve (Medtronic, Minneapolis, MN) in patients with stenosis of a previously placed bioprosthetic valve in the tricuspid valve position who were deemed high-risk surgical candidates.

**Methods:** A retrospective chart review was undertaken of patients who underwent TVIVI at our institution between 2016.and 2020. Baseline clinical characteristics, procedural details, and outcomes at 1 month, 6 months, and 1 year were reviewed. Statistical analyses (dependent *t* test) to assess tricuspid valve gradients before and after TVIVI and at 1, 6, 12, 24 months of follow-up were performed. A *p* value < 0.05 was considered significant.

**Results:** Four patients, ages 17 to 66 years (37.5 + 10.2; mean + SEM), identified to have severe stenosis of a previously placed bioprosthetic valve in the tricuspid position underwent TVIVI with a 22-mm Melody valve. Three patients were NYHA functional class 4 and 1 patient was class 3 prior to intervention. All patients had multiple associated comorbidities and were considered high risk for cardiac surgery. Cardiac catheterization data obtained prior to and after TVIVI demonstrated that the peak-to-peak gradient across the tricuspid valve decreased from 14.5 + 3.3 to 5.25 + 1.25 mmHg (mean + SEM; *p* value = 0.02). By echocardiogram the mean gradient across the tricuspid valve decreased from 12.0 + 1.41 to 5.0 + 1.08 mmHg (mean + SEM; *p* value = 0.007). The mean transvalvar gradient by echocardiogram was unchanged at a follow-up of 3–25 mo (5 + 4.3; mean + SEM). One patient underwent Melody valve placement in the pulmonary valve position during the same procedure. The only complication was transient complete heart block in one patient, which resolved within 12 h. No other complications were identified at a mean follow-up of 5 months. Hospital length of stay ranged from 1 to 13 days, with a mean of 4.5 days (SEM + 2.8). Cardiovascular symptoms resolved in 3 patients and 2 patients had improvement in NYHA functional class. One patient, with a functional single ventricle (unbalanced AV septal defect, severe AV bioprosthetic valve stenosis) had liver cirrhosis, chronic ascites, and deteriorating renal function. Following TVIVI, he had improvement in renal function and is now listed for heart and liver transplant.

**Conclusion:** Patients undergoing tricuspid valve replacement (TVR) using bioprosthetic valves are at a risk for valvar degeneration and may need repeated interventions for tricuspid valve stenosis or regurgitation. Surgical replacement of the valve may be associated with significant morbidity and mortality in patients with poor functional class and multiple comorbidities. Our experience thus far suggests that TVIVI is a safe and effective alternative to surgical TVR without valvar restenosis or regurgitation at short-term follow-up. TVIVI may obviate the need for surgery or delay in intervention, and should be considered as the treatment of choice in select patients.

## 53

### Optimal Criteria of Transcatheter Closure of Fontan Fenestration—A Single-Center Experience with a Review of Literature

Yuki Kawasaki^1,2^, Takeshi Sasaki^1,2^, Thomas Forbes^1^, Robert Ross^1^, Daisuke Kobayashi^1^

^1^Children’s Hospital of Michigan, Detroit, USA. ^2^Osaka City General Hospital, Osaka, Japan

**Abstract**

**Background:** Fenestration closure is considered to remove the persistent right-to-left shunt after the Fontan operation. However, criteria for effective transcatheter closure of fenestration to avoid both of acute and chronic Fontan failure have not been clarified enough.

The objective of this study was to describe the hemodynamic data with test occlusion of the Fontan fenestration between closure and non-closure patients along with subsequent development of Fontan-associated diseases (FAD) at follow-up.

**Methods:** This was a retrospective study to assess the outcome of Fontan fenestration closure at Children’s Hospital of Michigan over 27 years (1993–2019). The inclusion criteria were patients undergoing cardiac catheterization for an indication of fenestration closure. The data were compared between two groups: closure vs. non-closure. Baseline characteristics and hemodynamic variables with the fenestration occlusion test were analyzed. The primary outcome was the development of the composite events of death/transplant, deteriorated NYHA or FAD.

**Results:** Among the 38 patients who were brought to the catheterization laboratory, 33 received fenestration closure and 5 did not. On a median follow-up of 3.4 years (range 1 month to 12.6 years), the incidence of primary adverse outcomes was 13% (5/38). The incidence of primary outcome was significantly higher in the non-closure group (60% vs. 6%, *p* < 0.01). The non-closure group had a higher incidence of moderate or severe atrioventricular valve regurgitation, NYHA class III symptoms, use of ACEI/ARB, furosemide, and sildenafil. Multivariable logistic regression model showed that the hemodynamic variables associated with non-closure group were mean left atrial pressure (odds ratio 1.74, *p* < 0.05) and change of mean Fontan pressure at the balloon occlusion (2.2, *p* < 0.05).

**Conclusions:** The judgment for the fenestration closure appeared appropriate in our cohort. Fontan fenestration closure may not be advisable with a high baseline left atrial pressure or a significant increase of Fontan pressure on balloon occlusion testing.

## 54

### Creating Two Transcatheter Fenestrations and Simultaneous Colocation of Pacemaker Through One of Them in a Fontan Circulation

Marco Ruiz-Ontiveros, Alejandro Flores-Arizmendi, Antonio Salgado-Sandoval, Jesus Montalvo-Aguilar, Enrique Zuñiga-Guerrero

National Medical Center “20 de Noviembre” ISSSTE, Mexico City, Mexico

**Abstract**

**Introduction:** Transcatheter creation or dilatation of a fenestration after a Fontan procedure is described to improve clinical condition when patients present deterioration with development of protein-losing enteropathy, pleural effusion, ascites, or plastic bronchitis. Another complication present in the short and long term is the multiple type of arrhythmias, the treatment can be pharmacological or in some cases may need an ablation or in rare cases the use of pacemaker.

**Case Report:** Male of 18 years old, with diagnosis of tricuspid atresia, fenestrated Fontan surgery on June 2019, one year later, the patient presented with clinical deterioration, with bilateral pleural efussion and ascites, in the echocardiogram with no obstruction in the Fontan but with no evidence of fenestration. We decided to make a percutaneous fenestration in the extracardiac conduit.

Under general anesthesia and mechanical ventilation, we obtain femoral access. There were no obstructions in any site of the Fontan circuit but the mean pressure was 25 mmHg, in the angiography with no evidence of the surgical fenestration, so with a Brockenbrough needle advanced through a 6 Fr long sheath to the selected perforation site of the extracardiac conduit, a PT choice 0.014 coronary wire was advances through the needle and the right atrium and positioned in the left atrium, next, the needle, and the dilator of the long sheath were carefully exchanged for a 5-mm balloon catheter to pass and dilate the conduit and the atrial wall. Afterwards, the balloon catheter was exchanged for a 7-mm and then a 10-mm balloon catheter to further gradually dilate the initially established communication.

In the subsequent days, the patient presented a complete AV block, with the indication of colocation of a pacemaker, because of the risks, we decided a transcatheter implantation and the only access was the recent perforation, but the cables would obstruct almost the whole fenestration. The second procedure was performed in similar conditions, the previous fenestration was permeable, also with a Brockenbrough needle the second perforation was made above the first one, and the balloons catheters used to dilate were 6, 8, and 10 mm. We left the coronary wire to advance de pacemaker and position it at the atrium and left ventricle. After the procedure the patient’s clinical condition improved considerably, the control EKG demonstrated the normal function of the pacemaker.

**Conclusion:** We present a rare and success case of a double-transcatheter perforation in a Fontan conduit, the first one to improve the clinical condition (pleural effusion an ascites), and the second one to place a bicameral pacemaker because of a complete AV block with hemodynamic repercussion. The patient improved his functional capacity and he egress with no evidence of effusions.

## 55

### Non-surgical, transcatheter vessel anastomosis in congenital heart disease

Peter W. Guyon Jr.^1^, Caitlin M. Heyden^1^, Robert J. Lederman^2^, Howaida G. El-Said^1^, John W. Moore^1^, Kanishka Ratnayaka^1^

^1^Division of Pediatric Cardiology, Rady Children’s Hospital| University of California San Diego, San Diego, USA. ^2^Cardiovascular Branch, Division of Intramural Research, National Heart, Lung, and Blood Institute, National Institutes of Health, Bethesda, USA

**Abstract**

**Background**: Transcatheter electrosurgery uses radiofrequency energy to cut tissue using off-the-shelf guidewires and a standard electrosurgery unit. Recent adult structural heart applications include transcaval access, BASILICA, and LAMPOON. Non-surgical, transcatheter vessel anastomosis is a potential congenital heart disease application.

**Methods:** Radiofrequency energy from a standard electrosurgical generator (System 5000, Conmed, Utica, New York) was used with commercial 0.014″ guidewires (Astato, Asahi Intecc, Santa Ana, California) to cross vessel walls for large vessel end-to-side anastomosis, rescue re-anastomosis between adjacent vessels, and temporary anastomosis between non-adjacent vessels for vascular access.

**Results:** Transcatheter electrosurgery was successful in enabling anastomosis of donor vessels measuring 16.7 mm–19.0 mm in diameter to recipient vessels measuring 10.2 mm–19.6 mm in diameter. Vessels were 0–20.2 mm apart.

In an adult congenital heart disease patient, superior vena cava exit and right pulmonary artery entry followed by subsequent endografts created a permanent superior cavopulmonary anastomosis with anastomotic diameter of 20.7 mm. In a second (pediatric) patient, transcatheter rescue re-anastomosis was performed after inadvertent right pulmonary artery occlusion following transcatheter pulmonary valve insertion. Distal endograft wire perforation from the main pulmonary artery followed by balloon angioplasty created a 7.0 mm anastomosis with 6 mmHg residual pressure gradient into the right pulmonary artery while preserving implanted valve function. In a third case, transcatheter electrosurgery established temporary (“transcaval”) vascular access between the inferior vena cava and abdominal aorta (spaced 20 mm apart) in a patient with insufficient arterial vascular access. This facilitated temporary 20 French arterial access for endograft placement to dilate a stenotic surgical interposition graft and exclude a compressive seroma.

There were no procedural transcatheter electrosurgery complications. All three patients were extubated immediately after their procedures. The superior cavopulmonary anastomosis patient was hospitalized for 112 h (4.6 days) while the other two patients were hospitalized for 36 and 37 h, respectively. At time of most recent follow-up (576 ± 458 days), there were no complications.

**Conclusions:** Transcatheter electrosurgery can be used safely for large vessel end-to-side anastomosis, rescue re-anastomosis, and temporary anastomosis for vascular access. We demonstrated success in directly adjacent vessels as well as more remote vessels requiring traversal of extra-vascular space. Wire traversal was performed through native, naïve vessels, and synthetic material (*Melody*, Medtronic, Minneapolis, Minnesota).

## 56

### Non-Elective Pediatric Cardiac Catheterization during COVID-19 Pandemic: A New York Center Experience

Kristin Oshiro, Mariel Turner, Alejandro Torres, Matthew Crystal, Julie Vincent, Oliver Barry

NewYork Presbyterian Morgan Stanley Children’s Hospital, New York, USA

**Abstract**

**Background:** COVID-19 has led to major changes in hospital systems across the world. In an effort to reduce viral transmission, conserve resources, and in accordance with institutional and state mandates, all elective procedures and surgeries were postponed during the initial outbreak. Guidelines for case selection are limited and management for pediatric catheterization laboratories during this crisis is unprecedented.

**Objectives:** To report the protocols and case selection of a high-volume pediatric cardiac catheterization laboratory in the epicenter of the novel coronavirus (COVID-19) pandemic.

**Methods:** All pediatric cardiac catheterization procedures from March 16 through May 10, 2020 were reviewed. Changes to case selection and periprocedural workflow were described. Data were collected on COVID-19 testing status, primary procedure type, and all procedures were classified by urgency.

**Results:** There were 52 catheterization procedures performed on 50 patients. Endomyocardial biopsies were the most common procedure (*n* = 27, 52%). Interventional and diagnostic procedures represented 27% (*n* = 14) and 21% (*n* = 11) of cases, respectively. Two emergent procedures (3.8%) were performed on patients with positive COVID-19 testing. Most cases were performed on patients with negative COVID-19 testing (*n* = 33, 94%).

**Conclusions:** Adjusting to the COVID-19 pandemic in a high-volume pediatric cardiac catheterization laboratory can be safely and effectively managed by prioritizing emergent and urgent cases and modifying workflow operations. The experience of this center may assist other pediatric cardiac catheterization labs in adapting to similar practice changes as the pandemic continues to evolve.

## 57

### Development and Testing of the Renata Minima Stent and Delivery System: A New Neonatal Stent Capable of Achieving Adult Dimensions

Evan Zahn, Eason Abbott, Neil Tailor, Shyam Sathanandan, Dustin Armer

Cedars-Sinai Medical Center, Los Angeles, USA

**Abstract**

**Background:** There are no endovascular stents designed or approved for use in infants and neonates, nor is there a stent capable of being implanted at neonatal diameters and achieving adult size while maintaining structural integrity. The Renata Minima stent was designed specifically to address these issues.

**Methods:** The Minima stent is a balloon expandable, cobalt-chromium stent that comes pre-mounted on a proprietary 4F covered delivery system designed to track over a standard 0.014″ or 0.018″ guidewire. Bench-top expandable diameter ranges from 4 to 22 mm. This study was performed in 6 infant pigs to examine 1) deliverability and implant performance, 2) early histopathologic pathologic response, 3) *in vivo* re-dilation potential and stent behavior, and 4) chronic histopathologic pathologic response in both re-expanded and non-re-expanded stents.

This study utilized final stent designs, however, some manufacturing processes were modified between experiments.

**Results:** Twenty-four stents (22 Minima and 2 control) were implanted into the aorta (*n* = 13), branch pulmonary arteries (*n* = 6), and central veins (*n* = 5) of 6 infant piglets with an average weight of 4.6 kg. One piglet had an LPA stent which migrated to the MPA and underwent no further instrumentation and is not included in subsequent analyses. The remaining piglets were divided into 3 categories: 1) Early feasibility, single re-expansion, and sacrifice at 1 month (*n* = 2 piglets, 10 stents); 2) Serial re-dilation performed at 2–3 mos and 5 mos after implant (2 piglets, 9 stents); and 3) Single dilation 5 months after implant (1 piglet, 4 stents). All stents were successfully deployed into the target vessel, excluding 1 branch pulmonary artery stent, which utilized an early delivery system prototype. There were 2 early instances of proximal LPA stent migration following successful deployment during removal of the delivery balloon. Median implant diameter was 7.5 mm (2.6–8.8 mm). Stents were re-expanded at 34, 64, 82, and 154 days, corresponding to average animal weights of 14, 35, 44, and 107 kg. All Minima stents regardless of location could be re-dilated to keep pace with somatic growth (Figure). There was a single Minima stent that appeared incompletely opposed to the abdominal aortic vessel wall 64 days after implant. Histopathology at 34 and 146 days (3 animals, 8 vessels) demonstrated widely patent stented vessel lumens with uniform stent apposition to vessel wall, early (1 mos) mild inflammatory response surrounding stent struts, typical progressive vascular healing response, and a progressive smooth neointimal growth pattern covering stent struts over time (2–3 mos).

**Conclusions:** This early work suggests that the Minima stent and 4F delivery system may be well suited for treatment of a variety of vascular stenoses in young infants and neonates. This is a unique device in that it can be enlarged in vivo, from neonatal to adult diameters while maintaining structural integrity. Further laboratory and clinical testing will be needed to determine the safety and efficacy of this novel device in humans.
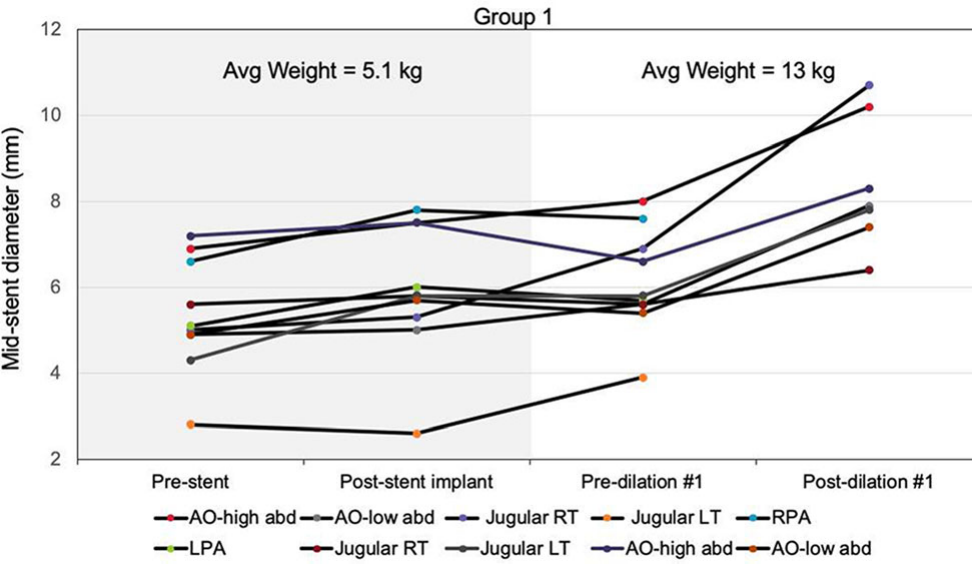

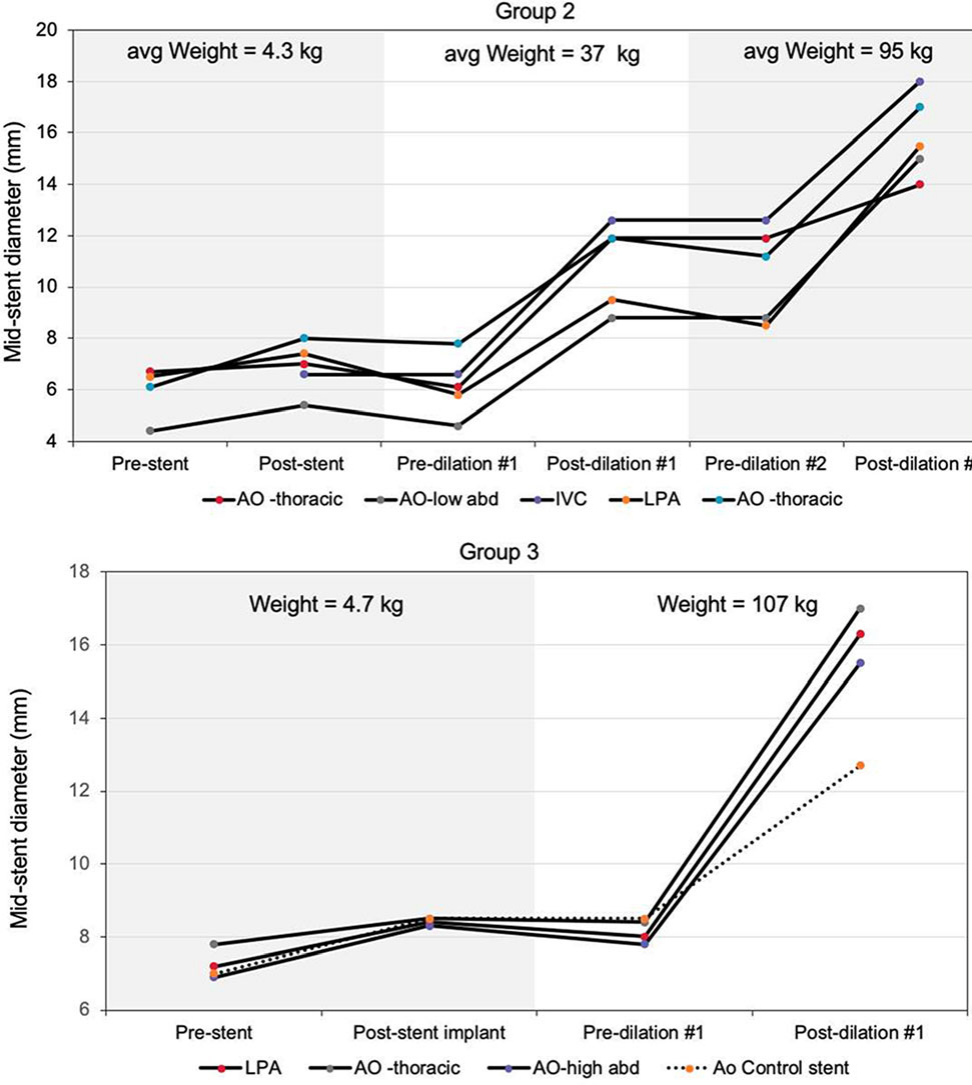


Do you think we need to add a blurb like this about the varying manufacturing processes—which led to the inability to grow past 18 mm and accounted for higher recoil in some stents?

I like it

This stent was one that was left in the vessel for 5 months without re-expansion

Fixed this…I think

## 58

### Serum Sirolimus Levels After Implantation of Third-Generation Drug-Eluting Cobaltchromium Coronary Stent in Ductus Arteriosus in Neonates with Duct-Dependent pulmonary Circulation

Rajesh Kumar Ramaswamy^1^, Sivakumar Kothandam^2^

^1^Madras Medical Mission, Chennai, India. ^2^Madras Medical Mission, Chennai, India

**Abstract**

**Objectives:** Ductal stenting(DS) palliates duct-dependent lesions using coronary stents. Sirolimus-eluting stents have replaced bare-metal stents in coronary interventions. Concerns exist about sirolimus levels in neonates. Therapeutic immunosuppressive sirolimus level is 5–15 ng/ml.

**Methods:** After neonatal DS, drug levels were assessed at 24 hours, 7 days, and monthly thereafter till they were undetectable. Clinical course, ductal patency till their final corrective surgery was analyzed. The exact quantity of sirolimus in each stent was known.

**Results:** Twelve neonates with median age of 5.5 days received sirolimus-eluting stents, one stent in nine and two in the rest. The lesions were pulmonary atresia intact ventricular septum(PAIVS) in four, univentricular lesions with pulmonary atresia in four, biventricular lesions with pulmonary atresia in three, and right ventricular rhabdomyoma in one neonate. If single stents up to 22 mm length, 24-hour drug levels were less than 5 ng/ml. Even though 24-hour levels were above 5 ng/ml in patients with single longer stent or two stents, it reduced to very low levels by seventh day. Two hospital deaths included rhabdomyoma with complete heart block and post-valvotomy cardiac failure for PAIVS. Stent patency after valvotomy for PAIVS exceeded three years. Patency was retained for 8–27 months till their elective corrective surgery in others.

**Conclusions:** Sirolimus levels were acceptable at 24 hours in all neonates receiving single stent under 22 mm length. In patients needing two stents, drug levels were in immunosuppressive range at 24 hours but reduced rapidly within 7 days. The palliation provided by sirolimus-eluting DS was sufficiently long to provide clinical benefit.

## 59

### Identical cardiac lesions with divergent physiology in a monozygous/diamniotic pregnancy

Robert Petersen^1^, Beth Price^2^, Chetena Reddy^1^

^1^St. Louis University, St. Louis, USA. ^2^SSM Health Cardinal Glennon Children’s Hospital, St. Louis, USA

**Abstract**

**Introduction:** This case study reviews a monozygous/diamniotic pregnancy resulting in twins with identical cardiac lesions with divergent physiology.

**Case Presentation:** Fetal care Institute patient with pregnancy complications including monozygous/diamniotic pregnancy, NIPT positive for Trisomy 21 in both twins, gestational diabetes, and growth restriction of Twin A. The fetal ECHO on both twins showed suspected Tetralogy of Fallot and with arch hypoplasia in Twin A. They were born at 30 weeks of gestation due to preterm labor. Postnatal Trisomy 21 was confirmed on both twins. Postnatal echocardiogram for Twin A showed a moderate perimembranous ventricular septal defect (VSD) (3.7 mm) with bidirectional flow, small-to-moderate PDA with bidirectional shunting, mostly left to right. Patent foramen ovale with left-to-right flow. No left heart enlargement. Mild tricuspid regurgitation, estimated RVSP 52 mmHg + RAp. Normal biventricular systolic function. The findings on twin B after delivery found moderate perimembranous VSD (3.5 mm) with bidirectional flow, mostly left to right, partially covered by tricuspid valve tissue. Moderate PDA with bidirectional shunting, mostly left to right. Patent foramen ovale with mostly left-to-right flow. No left heart enlargement. Mild tricuspid regurgitation, with estimated RVSP 54 mmHg + RAp. Normal biventricular systolic function. Both spent 3 months in the NICU due to feeding difficulties resulting in a G-tube placement and respiratory failure. Both required Lasix while in the NICU for clinical signs of pulmonary overcirculation. Both failed weaning from respiratory support and were sent home on 1/8L nasal cannula. At their first outpatient clinic visit, both had low velocity flow across their VSD and a soft murmur on exam concerning for elevated right heart pressures. Prior to undergoing repair, both underwent a cardiac catheterization. The catheterization findings on Twin A showed a moderate perimembranous VSD, moderate secundum ASD, mildly elevated RVEDP (11 mmHg), elevated PA pressure (40–42 mmHg), normal PVR (1.92 iWU), upper normal LVEDP (10 mmHg), Qp:Qs = 4.25:1. The findings on Twin B showed a large perimembranous VSD with baseline Qp:Qs 2.27:1, elevated pulmonary vascular resistance at baseline 5.7 Woods units × m^2^, pulmonary vasodilator testing with iNO 40 ppm and FiO_2_ 60% demonstrated improvement in PVR to high normal level 2.7 iWU and decreased cardiac output 2.4 L/min/m2. Based on the information found during the catheterization twin A underwent surgical closure of VSD, closure of ASD, and ligation of the PDA. Based on the information found during the cardiac catheterization, Twin B was started on Tadalafil prior to repair. Twin B underwent closure of an ASD and VSD and ligation of PDA. The post-operative course for both twins was uncomplicated.

**Conclusion:** Both twins had identical cardiac lesions, NICU hospital issues including respiratory failure, echocardiogram findings with low velocity shunting across the ventricular septal defect with a soft murmur. However, their hemodynamics were drastically different with Twin A with a Qp:Qs of 4.25:1 and significant pulmonary overcirculation and Twin B with an elevated PVR at baseline that was reactive to pulmonary vasodilators and started medication prior to surgery. This represents a unique clinical case presentation.

## 60

### Pericardiocentesis in Children: A 20-Year Single-Center Experience

Christopher Herron, Daisuke Kobayashi

Children’s Hospital of Michigan, Detroit, USA

**Abstract**

**Background:** Pericardiocentesis is the invasive percutaneous procedure for acute and chronic excessive accumulation of pericardial fluid. The hypothesis of this study was that pericardiocentesis be effective and safe in children with low risk of complications and certain clinical factors be associated the development of significant adverse events (SAE).

**Objectives:** To describe the single-centered experience of pericardiocentesis over the 20-year period (2001 to 2020) and evaluate the effectiveness and safety of pericardiocentesis and factors associated with development of SAE.

**Methods:** This was a single-centered retrospective study to describe all the children aged ≤ 20 years who underwent pericardiocentesis at the bedside and in the cardiac catheterization laboratory. Data on demographics, etiologies of pericardial effusion, repeat intervention at follow-up were collected. Technical procedural success was defined as satisfactory removal of effusion at pericardiocentesis. Logistic regression model was used to evaluate the factors associated with development of SAE and repeat intervention.

**Results:** A total of 127 patients underwent 153 pericardiocentesis procedures. The mean age was 6.5 ± 6.7 years with weight of 27.8 ± 28.6 kg. Most common etiology was post-pericardiotomy syndrome (*n* = 56, 44%), followed by Infectious (12%), Malignant (10%), and Iatrogenic (9%). Clinical and echocardiographic cardiac tamponade were present in 53% (80/153). Pericardiocentesis was performed primarily in the catheterization laboratory (*n* = 86, 59%) and in the high acuity (emergent 9%/urgent 91%). Concurrent pericardial drain placement was performed in 67 patients (53%) at the initial pericardiocentesis. Acute procedural success was 93%. Repeat intervention was performed in 33 patients (26%). The incidence of SAE was 5% (7/149): hemopericardium requiring emergent surgery (*n* = 2), hemopericardium with hypotension (*n* = 2), seizure with anesthesia induction (*n* = 1), right ventricle puncture with needle (*n* = 2). No identifiable risk factors were associated with SAE.

**Conclusions:** Pericardiocentesis was life-saving in children with its high effectiveness and safety even in emergent and urgent situations. Although initial pericardiocentesis was effective, one of four patients required reintervention for recurrent pericardial effusion.

## 61

### Percutaneous Balloon Pericardiotomy in the Pediatric Cardiac Catheterization Laboratory

Christopher Herron, Thomas Forbes, Daisuke Kobayashi

Children’s Hospital of Michigan, Detroit, USA

**Abstract**

**Background:** Percutaneous balloon pericardiotomy (PBP) is a percutaneous procedure that creates a window in the parietal pericardium by balloon angioplasty. The use of PBP has not been reported well in children.

**Objectives:** The objective of this study was to describe the single-center experience of PBP in children.

**Methods:** This was a retrospective study to describe all the children aged < 20 years undergoing PBP during an 18-year period (2001 to 2019). Patient characteristics, technical and ultimate procedural success, and repeat interventions were collected.

**Results:** A total of 13 PBP’s were performed in 11 children at the median age of 12 years (range 1.8 to 19). The etiologies of pericardial effusion were post-pericardiotomy syndrome (*n* = 4), restrictive cardiomyopathy (*n* = 1), autoimmune diseases (*n* = 3), malignancy (*n* = 2), and idiopathic (*n* = 1). Two patients received two PBP. The technical success of PBP was 100% with no acute adverse events (balloon rupture or local bleeding). Five (45%) required reintervention and ultimately 3 required a surgical pericardial window 6 to 35 days after the PBP. As a result, ultimate procedural success rate was 73% (8/11).

**Conclusion:** PBP was performed safely with high technical success in children. PBP may be considered for recurrent and persistent pericardial effusion, before considering a surgical pericardial window.

## 62

### Correlation of Intravascular Ultrasound with Histology in Pediatric Pulmonary Vein Stenosis

Ryan Callahan, Zachary Gauthier, Shuhei Toba, Stephen Sanders, Sara Vargas

Boston Children’s Hospital, Boston, USA

**Abstract**

**Background**: Pediatric intraluminal pulmonary vein stenosis (PVS) occurs as a result of hyperplasia of myofibroblast-like cells in a myxocollagenous matrix. While angiography can only characterize the vein lumen, intravascular ultrasound (IVUS) can visualize the wall architecture and may help determine the cause of pulmonary venous obstruction. Preliminary in vivo IVUS images demonstrates luminal narrowing and wall thickening in patients with known PVS. It is unclear how the IVUS-delineated constituents of wall thickening correlate with the histology. We analyzed postmortem IVUS findings and correlated them with corresponding histologic sections in patients with PVS.

**Methods**: Formalin-fixed heart/lung specimens from 6 patients with PVS were identified in the Cardiac Registry. Control vessels included uninvolved PV from a PVS patient and uninvolved PV from an infant without PVS. The organs were submerged in water and target veins were imaged with IVUS using the Visions PV .014P RX digital catheter (Volcano Core Mobile System [Phillips Corporation, Amsterdam, Netherlands]). PVS specimens from the imaged areas were inked to maintain orientation and processed for light microscopic examination. Paraffin-embedded tissue sections were stained with H&E, Miller’s elastic (displayed in Figure 1), Masson trichrome, and for smooth muscle actin. IVUS images were correlated with histologic findings.

**Results:** The median age at death was 10 months (range: 4–21) and median postmortem weight was 4.7 kg (range: 3.91–9.58). Three had primary PVS, 2 congenital heart disease (1 TAPVC), and 1 prematurity/chronic lung disease. PVS veins previously underwent a median of 5 surgical/transcatheter interventions (range: 4–12) with recurrent stenosis extending into segmental branches on last angiography. In PVS veins, IVUS demonstrated wall thickening with up to two layers of variable echogenicity, often with indistinct borders. Histologically, the veins showed fibroblastic proliferation with areas rich in myxoid matrix as well as areas with abundant collagen and elastic fibers. Discrete vein layers were obscured by scarring and elastic degeneration. A lower reflective periluminal layer by IVUS corresponded with hyperplasia of myofibroblast-like cells in abundant myxoid matrix (Figure 1a/b/c. marked *, C = IVUS catheter). The hyper-reflective layer by IVUS extended to the outer edge of the vessel and corresponded to a less myxoid layer with more collagen, smooth muscle, and elastic fibers (Figure 1a/b/c. marked #). The outer less reflective edge of the IVUS image correlated with a gradual transition into adventitia. Normal veins had a thin wall, correlating with histologically normal cellular and extracellular components, without intimal proliferation (Figure 1d, M = striated muscle).

**Conclusion:** Our in vitro study identifies the histologic correlates to IVUS-defined areas of intraluminal PVS. IVUS may provide further understanding of the anatomy and mechanisms of pediatric pulmonary vein obstruction.
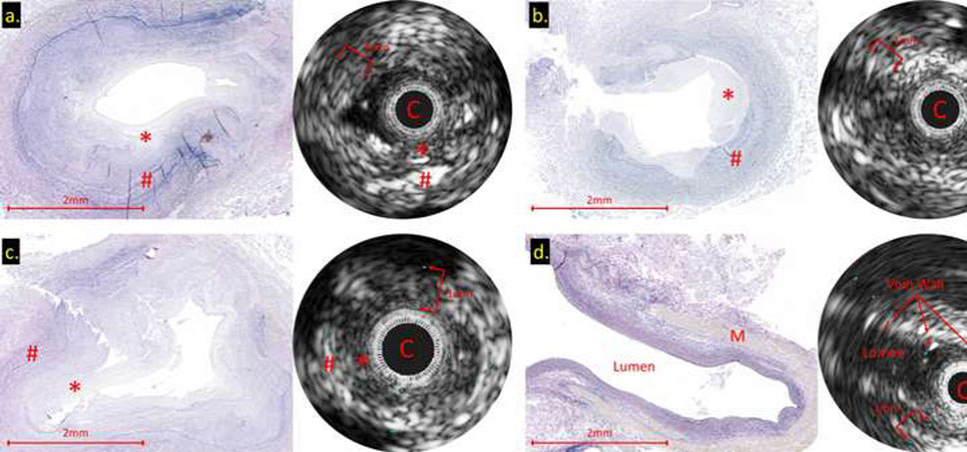


## 63

### Purpose-Built Transcatheter Cavopulmonary Anastomosis Device Requirements

Hannah El-Sabrout^1^, Justin Ryan^2^, Sanjeet Hegde^3^, Howaida El-Said^3^, John Nigro^3^, John Moore^3^, Kanishka Ratnayaka^3^

^1^University of California, Los Angeles, Los Angeles, USA. ^2^Rady Children’s Hospital San Diego, San Diego, USA. ^3^Rady Children’s Hospital San Diego/University of California, San Diego, San Diego, USA

**Abstract**

**Objectives:** This retrospective study reviews multimodality imaging to design a purpose-built transcatheter superior cavopulmonary anastomosis device. Patients with a functional single ventricle undergo multiple, palliative open-heart surgeries. This includes a superior cavopulmonary anastomosis or bidirectional Glenn shunt. A less-invasive transcatheter approach may reduce morbidity.

**Methods:** We analyzed pre-Glenn X-ray contrast angiography (XA), cardiac computed tomography (CT), and cardiac magnetic resonance (CMR) studies.

**Results:** Over an eleven-year period (1/2007–6/2017), 139 Glenn surgeries were performed at our institution. The typical age range at surgery was 59–371 days (median = 163; IQR = 138–203). Eight-nine XA, ten CT, and ten CMR studies obtained from these patients were analyzed. Cephalad SVC measurements (millimeters) were 7.3 ± 1.7 (XA), 7.7 ± 1.6 (CT), and 6.9 ± 1.8 (CMR). RPA measurements were 7.3 ± 1.9 (XA), 7.4 ± 1.6 (CT), and 6.6 ± 1.9 (CMR). Potential device lengths were 10.9 ± 6–17.4 ± 6.4 (XA), 10.1 ± 2.1–17.7 ± 2.4 (CT), and 17.3 ± 4–23.7 ± 5.5 (CMR). SVC-RPA angle (degrees) was 132.9 ± 13.2 (CT) and 140 ± 10.2 (MRI). Image quality of all CT (100%), almost all XA (SVC 100%, RPA 99%), and most MRI (SVC 80%, RPA 90%) were deemed sufficient. Parametric modeling virtual fit device with 10 mm diameter and 20–25 mm length was ideal.

**Conclusions:** Ideal transcatheter cavopulmonary shunt device for the typical patient would be 10 mm in diameter and 20–25 mm in length.
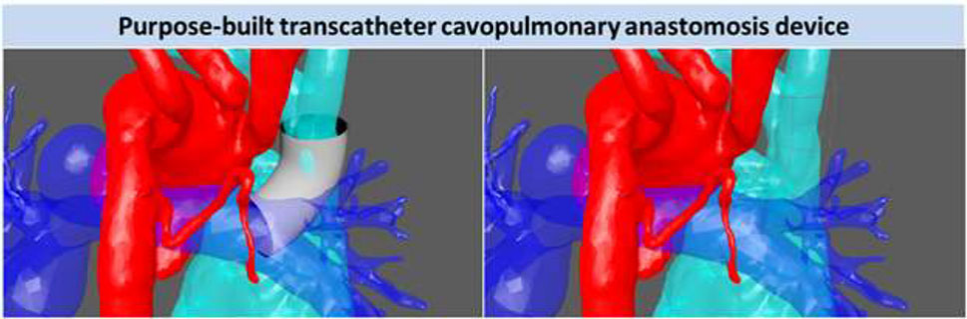


## 64

### Success of Coronary Sinus Os localization using Stepwise Approach

Najib AlRawahi^1^, Ghaliah Al Muhaini^1^, Mohammed AlRawahi^1,2^

^1^National Heart Center, Muscat, Oman. ^2^Sultan Qaboos University Hospital, Muscat, Oman

**Abstract**

Since 2008 implantable Cardiac Resychronization devices have been implanted exclusively in our center. We describe our experience in successful implantation of the left ventricular lead from 2015 to 2019. Our approach is using EGMs from a quad electrophysiology catheter which is successful in 96% of the cases. For the remaining cases, our approach is using different shapes of conventional diagnostic coronary catheters in localization of the Os then advancing a guide wire into the coronary sinus. Our stepwise approach was successful in 99% of the cases. For the remaining 1% complication of CS dissection was done which warrants a second procedure during which the implantation is done. We would like to share our approach to the world.

## 65

### Impact of Transcatheter Patent Ductus Arteriosus Closure On Aortic Stiffness In Premature Infants

Siddharth Mahajan, Shilpi Epstein, Dipak Kholwadwala, Aparna Kulkarni, Robert Koppel, Sharmeen Samuel, Deepank Sahni, Denise Hayes, Lindsey McPhillips

Cohen Children’s Hospital, Zucker School of Medicine, New York, USA

**Abstract**

**Background:** Aortic stiffness (AS) is a strong predictor of cardiovascular morbidity and mortality. The premature pediatric population is at high risk for cardiovascular diseases in early adulthood and has been shown to have increased AS compared to term infants. A patent ductus arteriosus (PDA) is a modifiable risk factor, common in premature infants, known to negatively impact AS.

**Aim:** The aim of this study was to investigate if transcatheter closure of a PDA in the premature infant population had an effect on the aortic stiffness index.

**Methods:** This is a single-center, retrospective study of all consecutive premature infants who underwent successful transcatheter PDA closure between 05/2019 and 02/2020. Aortic stiffness adjusted to body surface area (ASb) was calculated using the formula [(systolic blood pressure/diastolic blood pressure)*((Aortic systolic diameter-aortic diastolic diameter)/aortic diastolic diameter)]/body surface area. Measurements of the aorta were done using 2D echocardiography with a simultaneous blood pressure cuff recording. Regional ASb was recorded at the ascending aorta at the level of the right pulmonary artery (AscAo), transverse aorta between the innominate and left common carotid artery (TransAo), and descending aorta immediately distal to the isthmus (DistAo) from the long-axis suprasternal view. The proximal ascending aorta was also measured in the parasternal long-axis view above the sinotubular junction (PLXAscAo). Each regional ASb was recorded within one week prior to PDA closure, then post closure at one week and one month. Left ventricular dimensions and systolic function were measured on each study using M-mode and 5/6 area-length (AL). Statistical data are described using standard statistics and comparisons were made using unpaired two sample t test.

**Results:** Seventeen premature infants underwent successful transcatheter PDA closure during our study period. The mean gestational age at birth was 25.6 + 1.8 weeks and the mean corrected age at time of PDA closure was 32.1 + 4 weeks. Mean weight at the time of closure was 1674.3 + 925 g. The mean minimum diameter of the PDA was 2.5 + 0.6 mm. After closure, we found the indexed left ventricular end-diastolic diameter significantly decreased from 12.9 + 2.7 mm at baseline to 11.4 + 1.7 mm at 1 week, and 10.1 + 1.2 mm at 1 month (*p* = 0.04, *p* < 0.001, respectively). The left ventricular ejection fraction and shortening fraction remained within normal limits pre and post closure with no significant difference. ASb pre closure was highest in the TransAo (mean of 6617 + 3071). Post closure there was no significant statistical difference in the ASb at AscAo or PLXAscAo. There was a statistically significant decrease in ASb at TransAo (mean of 3550 + 1434, *p* = 0.0010) and DistAo (mean of 2892.8 + 1632.5, *p* = 0.0092) one month post closure. The average ASb post closure also demonstrated significant improvement at 1 month (mean of 3553.2 + 994.5, *p* = 0.0005) compared to the cumulative baseline (5256 + 1478).

**Conclusions:** Our study shows transcatheter PDA closure in a premature infant improves overall aortic stiffness, most markedly in regions of aorta that had increased flow due to the PDA. Further study may help identify optimal timing of PDA closure to impact aortic stiffness, and thus cardiovascular diseases in premature infants.

## 66

### Troponin I Elevation in Pediatric Cardiac Catheterization: Detecting Subclinical Myocardial Injury

Sarah Fahnhorst, DO, Mario Briceno-Medina, MD, Benjamin Hendrickson, MD, Shyam Sathanadam, MD, Hitesh Agrawal, MD

LeBonheur Children’s Hospital, University of Tennessee Health Science Center, Memphis, USA

**Abstract**

**Objective:** Cardiac troponin I release is associated with myocardial injury and has prognostic value in pediatric patients undergoing congenital heart surgery. There is little known data about troponin leak after cardiac catheterization in the pediatric population with the current high-sensitivity troponin I assay. We hypothesized that there would be differential troponin elevation with diagnostic, interventional, and endomyocardial biopsy procedures. The objective of this study is to compare troponin I release in pediatric patients undergoing diagnostic, interventional cardiac catheterization and endomyocardial biopsies and to establish norms following these procedures.

**Methods:** Prospective, single-center study of patients undergoing pediatric cardiac catheterization procedure between July 2019 and May 2020. Patients who met inclusion criteria were consented and enrolled in this study. Patients were divided into one of three groups: diagnostic, interventional cardiac catheterization, or endomyocardial biopsies. Troponin I samples were obtained prior to and immediately after the procedure. Electrocardiography was preformed post procedure to evaluate for ischemic changes. Demographic and clinical data were extracted from electronic records. The pre-procedural, post-procedural, and the difference between pre and post-procedure troponin I levels were analyzed between groups using non-parametric tests.

**Results:** Fifty-one patients, underwent a total of 62 procedures. There was a significant difference in post-procedure troponin I level between groups (*p* < 0.001). Diagnostic cardiac catheterization (*n* = 7) demonstrated minimal troponin release with no values exceeding upper threshold post procedure (median 0.012, IQR 0.012–0.012). Endomyocardial biopsies (*n* = 31) were associated with the highest elevation in troponin I level (median 0.2, IQR 0.114–0.259) compared to interventional cardiac catheterization procedures (*n* = 24, median 0.064, IQR 0.026–0.154). No procedure-related complications occurred during this study.

**Conclusion:** Utilizing a high-sensitivity troponin I assay, significant troponin elevation is detected in all pediatric patients undergoing endomyocardial biopsies and most patients undergoing interventional procedures. There was no significant troponin elevation following diagnostic heart cath procedures. Understanding the norms of troponin I release with each type of procedure can be useful to evaluate the myocardial health particularly if there is a concern for complications during the procedure.

## 67

### Comparison of Novel PLANE Technique Versus Standard Echocardiography Guidance for Pediatric Pericardiocentesis

Stephen Clark^1^, Santiago Borasino^1^, Jeffrey Alten^2^, Mark Law^1^

^1^University of Alabama at Birmingham, Birmingham, USA. ^2^University of Cincinnati College of Medicine, Cincinnati, USA

**Abstract**

**Background:** Percutaneous pericardiocentesis (PC) can be associated with rare, serious complications. Current standard practice PC involves echocardiography-guided (ECHO) or echocardiography-directed approach whereby the proceduralist is assisted by an echocardiographer or echocardiogram image, utilizing 2-dimensional phased-array ultrasound transducer directing needle entry, often not allowing continuous needle visualization through soft tissue/pericardial space. While this technique is described as safe and effective, we introduce a novel technique for PC using a L12-5 linear ultrasound transducer placed in a linear direction on the chest to allow for continuous visualization throughout the needle course into the pericardial space. In small complex critically ill patients, this allows for safe needle passage, avoiding abdominal viscera, lungs, and heart. With introduction of a new practice, we sought to compare the outcomes of standard ultrasound-directed PC versus a novel technique at a single center.

**Methods:** Retrospective chart review from March 2013 to March 2020 of pediatric patients undergoing PC at a single tertiary care facility following introduction of novel PLANE (Pericardiocentesis using Long-Axis iN-plane real-time Echocardiography) technique. PLANE technique procedures were compared to contemporary cohort of patients undergoing PC utilizing standard ECHO technique. Baseline patient and procedural characteristics were compared, in addition to outcomes, and were analyzed using SPSS.

**Results:** Seventy-four consecutive PC procedures were performed in 63 patients. Two procedures were excluded secondary to emergent nature without availability of ultrasound or echocardiographic guidance. Thus, 72 procedures were analyzed, of which 48 were performed using the PLANE technique and 24 using ECHO technique. There was no procedural mortality among either group. One minor complication was noted, asymptomatic small pneumopericardium that resolved with aspirating from pericardial drain. No significant differences in baseline characteristics (Table), except PLANE technique patients were significantly younger and smaller. PLANE technique tended to be utilized more in hemodynamically compromised patients and earlier in the post-operative period. Four procedures were performed in patients on extracorporeal support (ECMO), all of which utilized the PLANE technique. Interestingly, PLANE was by utilized by more non-interventional cardiology trained operators compared to ECHO (27% vs. 8% of procedures).

**Conclusions:** This large cohort of pediatric PC patients demonstrates that standard ECHO technique is safe and effective; the PLANE technique appears equally safe and may offer potential advantages in smaller, more critically ill patients. Additionally, this novel technique may be more easily mastered as suggested by its use in non-interventional cardiology trained practitioners.
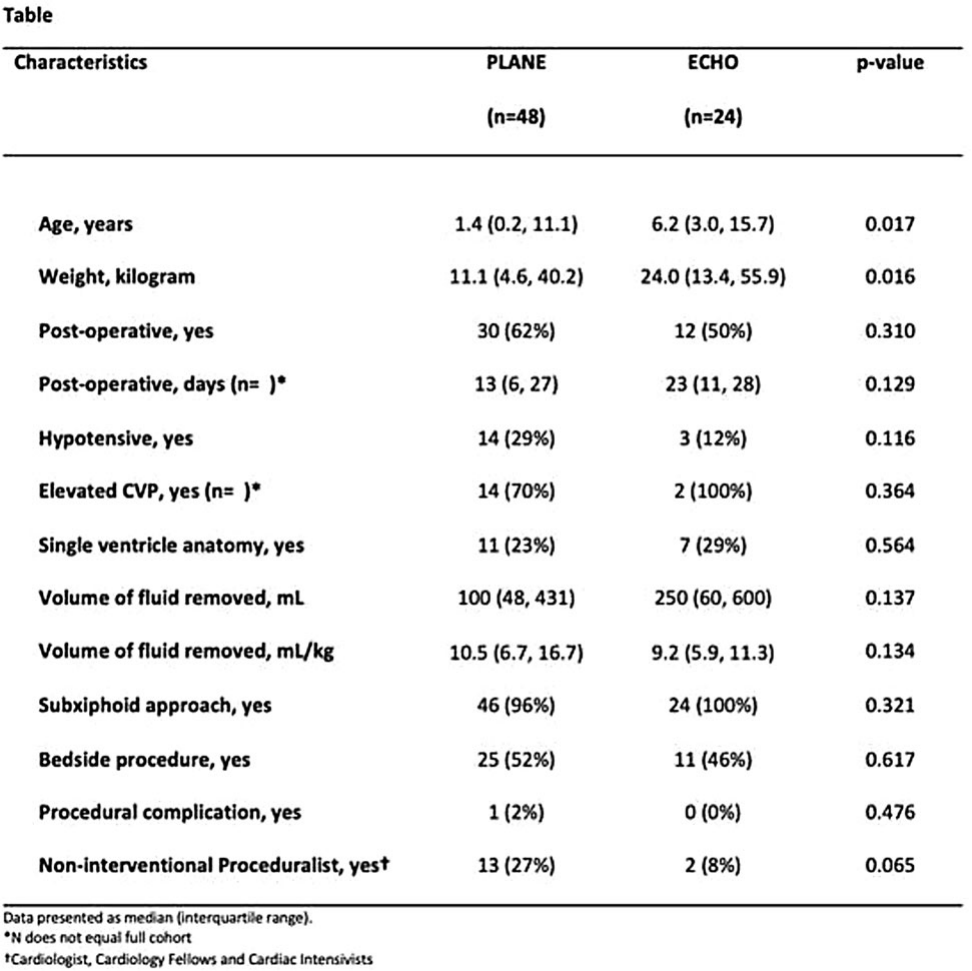


## 68

### Novel Ductal Guide Catheter with Specially Designed Geometry for Stenting of the Ductus Arteriosus in Newborns with Duct-Dependent Pulmonary Blood Flow: A Case Series Study

Diana Herrera-Valenzuela^1^, Camila Castro-Páez^1^, Juan Carlos Briceño^1^, Alberto García-Torres^2^

^1^Department of Biomedical Engineering, Universidad de los Andes, Bogotá, Colombia. ^2^Congenital Heart Disease Institute, Fundación Cardioinfantil, Bogotá, Colombia

**Abstract**

**Objective:** Evaluate the intraprocedural outcomes of using a novel ductal guide catheter with specially designed geometry for stenting of the ductus arteriosus (DA) as a palliative procedure in eight (8) newborns with duct-dependent pulmonary blood flow.

**Background:** Stenting of the DA in newborns with duct-dependent pulmonary blood flow is still challenging mainly due to the variety, ductal origin, morphology, and tortuosity of the ductus in these patients. Thus, we developed a novel ductal guide catheter with a shape specially designed to ease the palliative procedure in a wide variety of challenging DA.

**Methods:** Commercial guide catheters were modified through thermoforming into the ductal guide catheter shape. These were used for stenting of the DA in newborns with duct-dependent pulmonary blood flow treated at the Congenital Heart Disease Institute at Fundación Cardioinfantil (FCI) between 2017 and 2020. The decision to use the ductal guide catheter was made by the interventional pediatric cardiologist according to the patient’s medical status, the DA characteristics, and the diagnostic catheterization at the beginning of the procedure. The intraprocedural outcomes, clinical data, and the DA morphological characteristics were analyzed for each patient. The use of the ductal guide catheter was approved by the FCI’s ethical review committee and required informed consent.

**Results:** Eight (8) patients were analyzed. Ages ranged from 1 to 55 days (median = 6), and weight ranged from 2.4 to 4 kg (median = 2.95). Arterial blood oxygen saturation previous to the procedure ranged from 60 to 99% (median = 93.75). Two patients had a diagnosis of pulmonary stenosis, two had pulmonary atresia with ventricular septal defect, three had pulmonary atresia with intact ventricular septum, and one had pulmonary atresia with single ventricle morphology. Only one patient had a procedure prior to stenting. Four DA had aortic insertions at the aortic arch and four at the descending aorta. Six DA had a type 2 shape, one a type 3 shape, and one a type 1 shape according to the Qureshi et al. classification scheme. Angiography images of the eight ductus are presented in Figure 1. DA minimum diameter ranged from 1.6 to 5.11 mm (median = 2.41). Transductal gradient previous to procedure ranged from 13 to 90 mmHg (median = 41.5). Pulmonary arteries size ranged from 2.9 to 5.1 mm for the left pulmonary artery (median = 3.80) and 2.83 to 5 mm for the right pulmonary artery (median = 4.22). McGoon indexes ranged from 1.13 to 1.91 (median = 1.41). Femoral artery access was used in all the patients, however, the shape of the ductal guide catheter can be used for alternative vascular accesses that will be used in future interventions. No concomitant procedures were performed. The procedure was successfully completed in all patients and only two patients had minor complications, which were successfully controlled. Procedure time ranged from 59 to 262 min (median = 77). Patients required from 1 to 4 stents (median = 2). A total of 17 stents with either 3.5 or 4 mm diameter were used. All patients were monitored 4 to 207 days (median = 15) after the procedure was performed. Only one patient evidenced non-restrictive flux through the stent at follow-up.

**Fig. 1:**
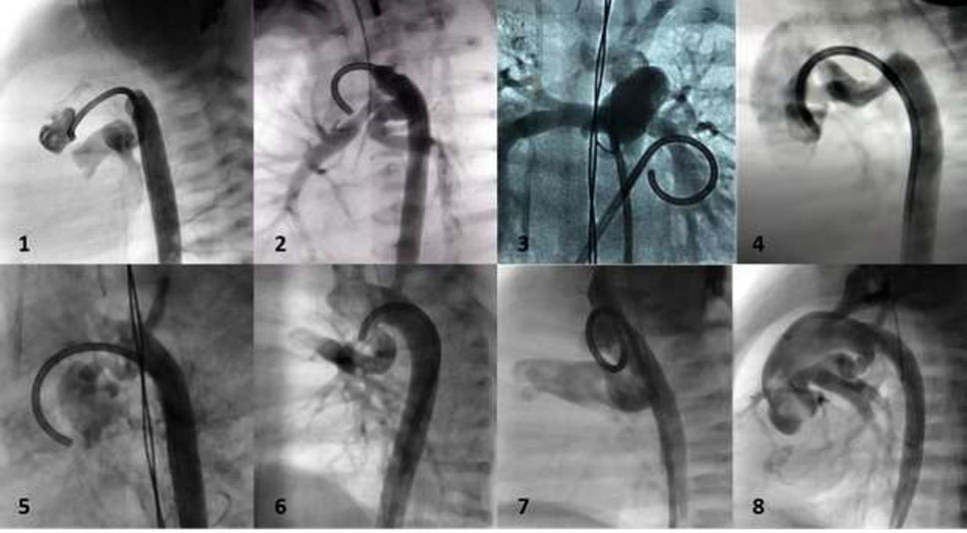
Angiography images of the ductus arteriosi of the eight newborns with duct-dependent pulmonary blood flow

**Conclusions:** Performing stenting of the DA as the first palliative procedure in newborns with duct-dependent pulmonary blood flow continues to be a challenge due to the patient’s critical medical condition and the complexity of their DA. Nevertheless, our novel ductal guide catheter allowed successful stenting of the DA in all the patients evaluated due to its specially designed shape. We are developing in vitro models of various DA anatomies to further test the versatility of our ductal guide catheter.

## 69

### Twenty-Year Experience in Cardiac Catheterization for Coarctation of the Aorta in Patients Less Than 25 Kilograms

Lindsay Eilers, Melissa Webb, Manish Bansal, Amna Qasim, Gary Stapleton

Texas Children’s Hospital/Baylor College of Medicine, Houston, USA

**Abstract**

**Background:** Transcatheter intervention for coarctation of the aorta (coA) has become standard therapy in larger patients and in patients with recurrent coarctation (re-coA). Studies have been published detailing the outcomes of catheter-based interventions for coA but none have focused on patients less than 25 kilograms (kg). The aim of our study was to describe our center’s short and medium-term outcomes in this patient population.

**Methods:** A retrospective review of patients less than 25 kg who underwent interventional cardiac catheterization for coA from 2000 to 2019 at our center was performed.

**Results:** One hundred and thirty-four patients underwent 182 transcatheter interventions for coA between January 2000 and December 2019. The median age (IQR) at cardiac catheterization was 7 months (29) and median weight (IQR) was 7.1 kg (7.9). Sixty-three patients (47%) had undergone single ventricle (SV) palliation and underwent 92 (51%) of the procedures. Of these procedures, 24 (26%) used a prograde approach, 66 (72%) a retrograde approach, and 2 (2%) used both approaches during the same procedure. The retrograde approach was via the femoral artery in all but 3 patients in which the carotid, axillary, or subclavian arteries were used. Of the total 182 interventions, 142 (78%) were for re-coA and 40 (22%) were for native coA. Balloon angioplasty alone was performed in 147 of 182 (81%) and stent placement occurred in 35 of 182 (19%) procedures. During a median follow-up of 40 months, 59 (44%) patients required reintervention: 44 (75%) for re-coA and 15 (25%) to maximize stent diameter. Twenty-five of the 44 patients with re-coA (57%) underwent surgical repair. There were 9 (4.9%) procedural complications including pseudoaneurysm of the coA site (1), second-degree atrioventricular block (1), post-procedure seizure (1), balloon rupture (1), aortic dissection (1), right external iliac artery transection (1), and cardiac arrest requiring CPR (3). There were no deaths related to the procedure. Post-procedural ultrasound of the accessed artery was performed after 21 (12%) procedures, with 11 arterial occlusions documented.

**Conclusion:** We describe a large cohort of patients who underwent transcatheter intervention for coA weighing less than 25 kg. These procedures may be safely and effectively performed in this group of patients as an alternative to surgical intervention. The need for reintervention is common.

## 70

### Ten-Year Follow-up following Catheter Closure of Perimembranous Ventricular Septal Defects Using the Amplatzer Asymmetric Perimembranous Ventricular Septal Defect Occluder in Children. In Memoriam

Basil (Vasileios) D. Thanopoulos^1,2^, George Tsaousis^3^, Nicholaos Eleptherakis^4^, Evangelos Karanasios^4^, Andreas Giannopoulos^5^

^1^Agios Loukas Clinic, Thessaloniki, Greece. ^2^Iatrikon Medical Center, Athens, Greece. ^3^Agia Sophia Children’s Hospital, Athens, Greece. ^4^Agia Sophia Children’s Hospital, Athens, Greece. ^5^AHEPA University General Hospital, Thessaloniki, Greece

**Abstract**

**Background:** During the last years, catheter closure of perimembranous ventricular septal defect (PMVSD) has been increasingly performed using a variety of modified Amplatzer asymmetric PMVSD occluders with satisfactory short-term results and safety. However, long-term results with the use of these devices are missing. We present 10-year follow-up (FU) with 95 patients (pts) with perimembranous ventricular septal defects (PMVSDs) who underwent transcatheter closure at 3 Institutions using the Amplatzer asymmetric PMVSD occluder.

**Methods**: The age of the pts ranged from 0.5 to 12 years. During the study period, 35 other patients were excluded from transcatheter closure because they did not fulfill the patient selection criteria (distance less than 2 mm from the PMVSD to the aortic valve, size of VSD in relation to patient’s age).

**Results**: The device was permanently implanted in 87/95 (92%) patients. Complete occlusion of the communication at six month was observed in 83/87 (95%) patients. Main complications included, Early: a. Device embolization (3 patients-catheter and surgical removal, respectively), b. severe procedural bradycardia (5 pts), and c. Mobitz II and complete heart block heart in 3 and 1 patients, respectively (observed in patients less than one year of age-body weight < 8 kg). (sinus rhythm after device removal). Late (follow-up 2–10 years): Complete heart block (required pacemaker implantation) was developed in one patient of 3 years old with posterior atrioventricular canal-type defect, no other patient developed heart block during the follow-up. Three patients developed mild aortic regurgitation, in one of them the regurgitation was not seen at the 1-year follow-up, no other complications were observed.

**Conclusions**: Transcatheter closure using the original Amplatzer APMVSD occluder is a safe and effective non-surgical alternative that can be used in carefully selected patients with PMVSDs. Despite the large variety of currently available Amplatzer-like PMVSD occluders this revolutionary designed device is still the most appropriate one for catheter closure of PMVSDs.

## 71

### Closure of Coronary Fistula Via Pulmonary Artery Using Double-Guide Wire Technique

Joshua Quinones^1^, Duraisamy Balaguru^1^, Amilcar Avendano^2^, Salman Arain^1^

^1^University of Texas McGovern Medical School, Houston, Texas, USA. ^2^Memorial Hermann Hospital, Houston, Texas, USA

**Abstract**

**Introduction:** Transcatheter closure of coronary fistula is an established procedure. However, closure of coronary fistula approaching from pulmonary artery side is considered difficult. We report transcatheter closure of coronary fistula via pulmonary artery using a double-guide wire technique.
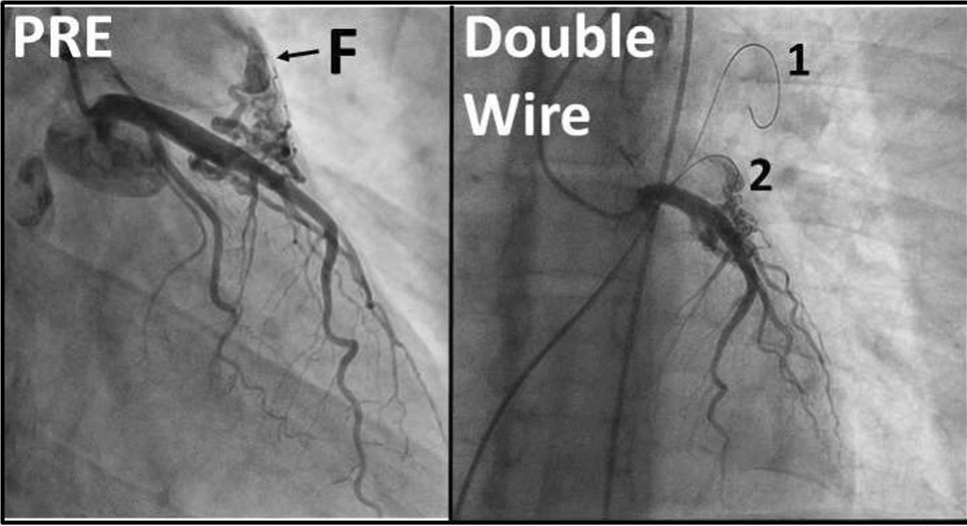


**Procedure:** A 28-year-old man with Klinefelter syndrome presented with recurrent chest pain over 2 years—associated with elevation of Troponin. Coronary angiograms performed 2 years apart showed two coronary fistulae. Fistula 1 had a bulbous portion (7 mm × 15 mm) which was fed by a mesh of vessels from left anterior descending artery (LAD) and drained to main pulmonary artery (MPA). Fistula 2 was fed by an accessory vessel originating very close to right coronary artery (RCA), and supplied conal myocardium before giving off branches that drained to MPA. Decision was made to close the coronary fistulae. Fistula 1 (“F” in Figure) was fed by very tiny mesh of vessels from LAD. Therefore, it was not possible to enter the fistula from left coronary artery. Therefore, decision was made to use a double-guide wire technique to enter from MPA side, 6 French JR4 guide catheter was used. First, a 0.014″ coronary guide wire was passed to distal LPA (“1” in Figure). This wire stabilized the catheter tip. The catheter tip was pulled opposite to the drainage point of the fistula. A second 0.014″ guide wire (“2” in Figure) was advanced into the fistula and stabilized inside the bulbous portion of fistula. The first guide wire was pulled back and catheter tip advanced into the fistula. After confirmation of catheter position, a Microvascular Plug 7Q was delivered in position. Complete occlusion of fistula was confirmed by left coronary angiogram. Fistula 2 was fed by an accessory vessel next to origin of RCA (not shown in Figure). First guide wire was advanced to distal RCA. Second guidewire was used to cannulate the accessory RCA vessel. Because this guide wire entered a different vessel than the feeder branch, a third guide wire was used to cannulate the feeder branch. When third guidewire was stabilized, the first two guide wires were removed and the catheter was advanced into the feeder branch. A Microvascular Plug 5Q was delivered using a microcatheter. Complete occlusion of fistula and the feeder vessels was confirmed by repeat angiogram. Patient was discharged home on Aspirin.

**Conclusion:** Use of double-guide wire technique enabled cannulation of vessels that were in unfavorable positions. Closure of coronary fistula by approaching from main pulmonary artery was accomplished using double-guide wire technique.

## 72

### Comparison of U.S. Hospital Costs Between Transcatheter Pulmonary Valve Replacement and Surgical Pulmonary Valve Replacement

Jennifer Williams, Pang Fang, MBA

Medtronic, Mounds View, USA

**Abstract**

**Background:** Given the development of new therapies to treat more eligible patients with transcatheter pulmonary valve replacement (TPV), there is great interest in understanding how overall inpatient hospital costs compare between TPV and surgical replacement (SPV).

**Methods:** To evaluate in-hospital costs across U.S. hospitals, we conducted a retrospective analysis of patients undergoing TPV or SPV between January 1, 2017–December 31, 2018 using the Premier Hospital Database. Patients were included in the study if they underwent a TPV or SPV procedure based on ICD-10 procedure codes. Patients were matched 1:1 using propensity score method based on patient age, Charlson comorbidity index grouping (4 indices), gender, race, and payor type. In-hospital costs were defined as the total hospitalization cost including operating room, supply, room and board, ICU, lab, etc., plus pharmacy cost, adjusted to 2019 dollars.

**Results:** We matched 490 TPV and SPV patients across 2017 and 2018 claims years. The average total inpatient hospital cost for TPV was 30% and 25% lower than the SPV comparator in 2017 and 2018, respectively (in 2018, TPV = $39,727, SD = $37,209 vs SPV = $53,313, SD = $28,492). The average total inpatient costs for TPV are lower than SPV, despite a higher average supply cost for TPV (in 2018, TPV = $38,763, SD = $21,547 versus SPV = $16,741, SD = $8095). TPV had lower average costs in many cost centers, including room and board (in 2018, TPV = $3833, SD = 8850 versus SPV = $14,294, SD = $10,747) and operating room (in 2018, TPV = $6392, SD = $7340 versus SPV = $14,116, SD = $7945). Average length of stay for TPV was 85% and 58% lower than SPV in 2017 and 2018, respectively (in 2018, TPV = 2.58 days, SD = 8.12 versus SPV = 6.09 days, SD = 5.31).

**Conclusion:** TPV demonstrates lower hospital costs than SPV, with the lower length of stay and lower costs of multiple cost centers offsetting the higher supply cost for TPV. These cost data demonstrate the necessity of hospital valve programs to consider total overall hospital costs of SPV and TPV versus focusing on supply costs.

## 73

### Complex Infradiaphragmatic VenoVenous Collaterals Following Fontan Palliation in the Setting of Inferior Vena Caval Obstruction

Sanchitha Guruchandrasekar, Kiran Mallula, Ernest Siwik

Louisiana State University, New Orleans, USA

**Abstract**

Complex infradiaphragmatic venovenous collaterals following Fontan palliation in the setting of inferior vena caval obstruction.

**Background:** Venovenous collaterals (VVCs) are a known common late cause of cyanosis following Fontan palliation in patients with univentricular physiology. Infradiaphragmatic collaterals utilize the azygous systems, hepatic veins, and portal circulation. Although abdominal VVCs have been described following Kawashima procedure, the prevalence in course is less known in patient with intact inferior vena cava (IVC). We report a unique series of three patients with complex VVCs related to IVC obstruction and their subsequent management.

**Case Presentation:** Three patients, status post Fontan procedure presented with cyanosis 5–15 years after the procedure. Their ages ranged between 10 and 19 years. The weight varied from 22 to 48 kg. Baseline saturations at the time of presentation were in the low to high 80 s.

(1) Patient 1 had a large VVC from the right internal jugular vein (RIJV) to the left atrium fed by tributaries from the azygous vein formed by collaterals from the paravertebral plexus inferiorly. Due to severe RIJV stenosis, bilateral femoral venous occlusion, and difficult access to the collateral, he underwent hybrid procedure for closure of the VVC with a 14-mm Amplatzer vascular plug II (AVP II).

(2) Patient 2 had severe stenosis of the IVC. This resulted in the formation of paravertebral venous collaterals that drained into the azygous vein and eventually into the right upper pulmonary vein. VVC was closed by deployment of two 14-mm AVP II devices in the azygous vein that was accessed through a paravertebral venous collateral followed by placement of seven stents in the IVC.

(3) Patient 3 had complete obstruction of infrarenal IVC. The paravertebral venous collaterals coalesced and drained into the left upper pulmonary vein. Percutaneous closure of VVCs in these patients was complicated by infrarenal IVC obstruction and difficult access to the VVC. After considering the complexity of the procedure to eliminate the collateral, weighing the risks and benefits of closing the VVC, a decision was made not to intervene.

The most recent follow-up of patients 1 and 2 were 11 and 1 months after the procedure. They reported a 10% increase in the baseline saturations and improved exercise tolerance.

**Discussion:** VVCs may cause systemic arterial desaturation and exercise intolerance. In the presence of IVC obstruction or bilateral femoral venous obstruction, infradiaphragmatic VVCs are difficult to uncover. A high index of suspicion is needed in Fontan patients with late cyanosis to discover the presence of these collaterals by angiographic or radiographic evaluation of abdominal venous system. Interventional palliation of these collaterals can be complex.

## 74

### Intravascular Ultrasound (IVUS) Provides the Filling for the Angiogram’s Crust: A Retrospective of the Benefits of IVUS in Pediatric Interventional Cardiology

Caitlin Heyden, Jonathan Brock, Kanishka Ratnayaka, John W. Moore, Howaida El-Said

Rady Children’s Hospital/UCSD, San Diego, USA

**Abstract**

**Background:** Intravascular ultrasound (IVUS) is a catheter-based imaging modality which generates cross-sectional views of vessel walls and lumens. This technique is utilized in adult interventional and vascular surgery frequently to assess extent of coronary artery disease and peripheral arterial disease and guide with management. IVUS has been described as superior to angiography in providing data about lesions of interest. The application of this tool to aid in interventional decision making for patients with congenital heart disease is scarce in the literature.

**Objective:** The purpose of this study was to review our experience using IVUS as an adjunct tool to diagnose lesions and assess results of interventions upon vascular structures in pediatric patients during cardiac catheterization in conjunction with traditional angiography.

**Methods and Results:** A retrospective chart review of all pediatric patients that underwent IVUS during cardiac catheterization to evaluate the cross-sectional lumen of non-coronary vessel(s) at Rady Children’s Hospital from January 2018–December 2019 was performed. Mean patient age 1076 days ± 1182 days with mean weight 12.1 kg ± 9 kg. A total of 26 vessels were interrogated with IVUS. Pathology evaluated included pulmonary venous stenosis (*n* = 8), coarctation (*n* = 5), branch pulmonary artery stenosis (*n* = 6), systemic shunts and conduits (*n* = 3), and other peripheral vasculature (*n* = 4). IVUS added value in 100% of the cases in which it was utilized. Use of IVUS guided intervention in 88% of the procedures and defined the endpoint in 62% of the procedures.

**Conclusions:** We found IVUS to be a safe tool for pediatric catheterization, with no complications during our study. IVUS was invaluable in enhancing our interpretation of identified pathology or in raising concern for occult lesions not seen on typical angiography. IVUS was particularly helpful in aiding with guiding interventional management and defining end point of interventions.

## 75

### Transcatheter Pulmonary Valve Implantation in Growing Children: Need for Reintervention and Effect on Valve Longevity

Asimina Courelli^1^, Arpine Davtyan^2^, Bryan Mosher^2^, Peter Guyon^2^, Kanishka Ratnayaka^2^, Howaida El-Said^2^

^1^University of California, San Diego School of Medicine, San Diego, USA. ^2^Division of Cardiology, Rady Children’s Hospital, San Diego, USA

**Abstract**

**Introduction:** Transcatheter pulmonary valve implantation in pediatric patients < 25 kg has been reported as a safe and effective procedure. However, limited investigation of treatment outcomes for these patients has been conducted. Since valve implantation is performed in growing patients, reintervention for somatic growth is expected. Therefore, we examined reintervention feasibility and resultant valve function.

**Methods**: Patients having undergone transcatheter pulmonary valve implantation (*Melody*, Medtronic, Minneapolis, MN) with weight at implantation of less than 25 kg at Rady Children’s Hospital in San Diego between January 2011 and December 2019 were identified. Patient profiles, short-term outcomes, and mid-term outcomes were reviewed.

**Results:** Thirty-four patients were identified who had undergone *Melody* valve implantation. The mean age was 6.8 ± 1.7 yrs and the mean weight was 20.7 ± 2.9 kg. Most patients were initially diagnosed with Tetralogy of Fallot (*n* = 16), which had been surgically repaired. The right ventricular outflow tract (RVOT) substrate was either native RVOT (trans-annular patch) (n = 17), conduit (*n* = 14), or bioprosthetic valve (*n* = 3). The indication for the Melody valve implantation was either regurgitation only (RO, *n* = 19), stenosis only (SO, *n* = 5), or both regurgitation and stenosis (R + S, *n* = 10). Implanted valve sizes were 22 mm (*n* = 14), 18 mm (*n* = 12), 20 mm (*n* = 6), 16 mm (*n* = 1), and 14 mm (*n* = 1). Immediately after implantation, no pulmonary regurgitation was noted on angiography or echocardiography for all patients and a significant decrease in RV-PA gradient (pre: 28 ± 7 mmHg, post: 15 ± 4 mmHg, *p* < 0.01) was observed. 3/34 patients had a complications: vascular access (*n* = 1), RPA jailing (relieved during same procedure) (*n* = 1), and conduit pseudo-aneurysm that required placement of vascular plug (*n* = 1). 5/34 patients developed transient non-sustained ventricular ectopy, none of which resulted in hemodynamic instability. Three of those five patients were treated with Nadolol and all arrhythmias resolved within 3 months. To date, only 2/34 patients required reintervention. One required reintervention 2 years post implantation for stenosis due to interval growth, which was treated with angioplasty, and the other required reintervention 6 years post implantation for stenosis due to interval growth and Melody frame fracture, which was treated with re-stenting, and Melody valve placement. 2/34 experienced Type I stent fractures, only one of which required intervention. At the time of most recent follow-up, on average 2.5 years (range 6 ms–6.6 ys), none of the patients required surgical intervention or developed Melody valve endocarditis. Echocardiography showed an average maximum RVOT gradient of 17 ± 9 mmHg in RO patients and 11 ± 8 mmHg in R + S patients with mild pulmonary regurgitation in only 3/34 patients. There was no patient mortality.

**Conclusion:** Melody valves implanted in growing pediatric patients have maintained their functionality and the recipients experienced low incidence of endocarditis with a few requiring reintervention for interval growth without significant complications.

## 76

### Percutaneous Left Ventricular Decompression in Venoarterial ECMO

Sharib Gaffar^1,2^, Joanne P Starr^1^, Roy Ramirez^1^, Danny Lam^1^, Michael R Recto^1^

^1^CHOC Children’s Hospital of Orange County, Orange, USA. ^2^University of California Irvine, Irvine, USA

**Abstract**

**Background:** Patients with severe left ventricular dysfunction requiring venoarterial extracorporeal membrane oxygenation (VA ECMO) may develop left atrial hypertension resulting in pulmonary edema, pulmonary hemorrhage, and increased wall stress leading to decreased coronary perfusion and increased mortality. Left ventricular decompression can be performed surgically or percutaneously. The percutaneous technique is performed in the cardiac catheterization laboratory by transseptal puncture with a Brockenbrough needle followed by blade septostomy. Static balloon atrial septostomy is then performed with progressively larger balloons until satisfactory atrial communication is created. We describe a novel approach to left heart decompression by placement of a catheter in the left ventricle, and attaching the catheter to the venous limb of a centrifugal ECMO circuit to actively unload the left atrium and ventricle. The left atrium is further decompressed by the percutaneously positioned catheter stenting open the previously created atrial communication, thereby improving left-to-right atrial shunting.

**Methods:** Three patients aged 8 months, 8 years, and 39 years (average 15.9 years), weighing 7.1, 20.2, and 65.8 kg (average 31 kg), developed severe left ventricular dysfunction and cardiopulmonary collapse requiring emergent placement on VA ECMO. Two patients developed respiratory failure and viral myocarditis—one from parainfluenza and one from influenza. The patient with influenza was converted from venovenous ECMO (VV ECMO) to VA ECMO due to acute biventricular decompensation. The third patient had congenital severe mitral regurgitation and undiagnosed left coronary artery ostial atresia, and arrested during diagnostic coronary angiography. After placement on VA-ECMO, all 3 patients developed left atrial hypertension based on echocardiographic and angiographic evidence of LV dysfunction and LA dilation. Transseptal puncture was performed with a Brockenbrough needle (Medtronic) followed by blade septostomy with Park Blade septostomy catheter (Cook Medical). Both procedures were performed with biplane fluoroscopy and transesophageal echo (TEE) guidance. Serial balloon dilation of the atrial communication with progressively larger balloons was performed. Pressure measurements in the LV, LA, and RA were then measured. TEE demonstrated suboptimal size of the atrial communication after the catheter was repositioned in the RA despite multiple blade and balloon atrioseptostomy procedures. The decision was then made to place a catheter with multiple side holes (pigtail catheter) across the atrial communication, into the LA, and then into the LV. The catheter was connected to the pre-pump venous limb of the centrifugal ECMO circuit. The catheter and venous sheath were then sutured in place. TEE confirmed proper catheter position.

**Conclusion:** Biplane fluoroscopy and TEE-guided transseptal needle puncture followed by blade septostomy and serial static balloon septostomy are effective techniques employed in creating atrial communication to relieve left atrial hypertension in patients requiring VA ECMO. In some patients, solely creating an atrial communication may not be sufficient to relieve left atrial hypertension. We describe a technique of left heart decompression by additional placement of a catheter into the left ventricle and connection of this catheter to the venous centrifugal ECMO circuit. The drainage catheter efficiently unloads the left ventricle and stents the atrial communication open, increasing left-to-right atrial level shunting when needed and minimizing ongoing LV dilation.

## 77

### Assessment of the State of the Cardiovascular System in the Elderly According to Computed Tomography

Volha Sujayeva, Irena Karpova, Anna Kravtchenko, Olga Koshlataya, Olga Spirina

Republican Scientific and Practical Centre Cardiology, Minsk, Belarus

**Abstract**

We studied 96 patients aged 70.6 (66.0; 73.0) years old, 32 (33.3%) were men, 64 (66.7%)—women. Grades 1–3 hypertension were present in 87 (91%), myocardial infarction—8 (8%), angina pectoris—23 (24%), PCI (6) (7%). Chronic heart failure Class I-II NYHA had 78 (81%). All included patients were statin-naïve. Computed tomography angiography of the coronary arteries (CTA CA) and estimation of Coronary Calcium Index (CCI) were performed on a dual-energy, 384-slice CT scanner of the premium class Siemens Somatom Force from General Electric Medical Systems (Germany). Standard biochemical blood test with determination of the lipid spectrum of blood, glucose, uric acid, determination of apolipoprotein A-1 (ApoA-1), and apolipoprotein B (ApoB) in blood serum was carried out by immunoturbidimetric method on a biochemical analyzer “ARCHITECTPLUS: 4000,” USA. Serum coenzyme Q levels were determined using the Human Coenzyme Q10 (CoQ10) Elisa kit reagent using an enzymatic method to determine the optical density at a wavelength of 450 nm (high-performance liquid chromatography).

The results of a biochemical blood test are presented in Table 1.

**Table 1:** Blood lipids in elderly (M, LQ-UQ)

Indicator	Result
Total cholesterol (TC), mmol/l	5.90 (5.13; 6.51)
High-density lipoproteins (HDL), mmol/l	1.38 (1.16; 1.56)
Low-density lipoproteins (LDL), mmol/l	3.98 (3.24; 4.61)
Triglycerides, mmol/l	1.59 (0.99; 1.83)
LP (a), mg/dl	17.1 (4.1; 18.8)
Apo A-1, g/l	1.81 (1.51; 1.89)
Apo B, g/l	1.27 (0.98; 1.27)
Coenzyme Q, mg/l	0.9 (0.6; 1.5)

Data of heart CT presented in Table 2.

**Table 2:** Heart CT in elderly (*N*,  %)

Indicator	Data
Coronary CT calcium scan (*N* = 63)	
Agatston score	*N* (%)
0–10	26 (34.7%)
11–100	16 (21.3%)
101–400	23 (30.7%)
More than 401	14 (18.7%)
Not carried out	19 (25.3%)
CT CA (*N* = 28)	
Stenosis < 70%, number of pts/number of segments	15 (53.5%)/41
Stenosis ≥ 70%, number of pts/number of segments	7 (25%)/7
Calcinosis of CA, number of pts (%)	21 (75%)/
Aortic valve calcinosis, number of pts (%)	19 (67.9%)
Mitral valve calcinosis, number of pts (%)	21 (75%)

We found positive moderate correlation between the levels of LDL and the CCI (*r* = 0.66, *p* < 0.), between the levels of TC and the CCI (*r* = 0.52, *p* < 0.), between the levels of LDL and CA significant stenosis (*r* = 0.29, *p* < 0.05). CCI was higher in smokers (*r* = 0.33, *p* < 0.), patients with diabetes mellitus (*r* = 0.30, *p* < 0.), and in alcohol abusers (*r* = 0.57, *p* < 0.*r* = 0.29, *p* < 0.).

**Conclusion:** Revealed high prevalence of clinical/subclinical manifestations of atherosclerosis in elderly people, which were interrelated with indicators of the blood lipid spectrum, dictates the need for statin therapy for both primary and/or secondary prevention.

## 78

### Validation of CRISP Score in Pediatric and Adult Patients with Congenital Heart Disease as a Predictor of Risk for Procedural-Related Serious Adverse Events at Our Institution—11-Year Retrospective Study

Shanique Sterling^1^, Yuen Y. Lo Yau Leung^1^, Alejandra Bueno^1^, Satinder Sandhu^2^

^1^Jackson Memorial Hospital, Miami, USA. ^2^University of Miami, Miami, USA

**Abstract**

Cardiac Catherization is an invaluable diagnostic and interventional tool in the management of patients with congenital and acquired heart disease. The CRISP Score is an essential pre- procedural risk scoring tool to stratify pediatric patients and identify those at risk for serious adverse events (SAE). We aim to internally validate this scoring tool at our institution in both our pediatric and adult patients with congenital heart disease.

A total of 2031 procedures between January 2008 to December 2018 were reviewed retrospectively and the CRISP Score calculated based on pre- and intraprocedural data. Complications documented within the immediate 24 h post procedure were identified. A SAE was defined as any adverse event causing mortality, permanent morbidity, need for further interventions, or extended length of stay. Microsoft Excel and SPSS were then utilized to facilitate performance of basic measures of descriptive analysis and binary logistic regression.

The cohort consisted of 1004 (49%) females and 1027 (51%) males with 66% being children (age < 18 years) and 34% being adults (> 18 years). Mean age for children was 7 years with a SD of 6.4 years, while for adults it was 30.3 years with a SD of 13.4 years. The age range for the cohort was 0–82.6 years.

There were 29 SAE (1.43% of total procedures). Children accounted for 82.7% of the SAE. The rate of SAE in children was 1.79% (24/1342) and in adults was 0.73% (5/689). The rate of SAE in children increased as age of the child decreased with neonates (< 1 month old) having a rate of SAE of 16.4% while children > 5 years had a rate of < 0.5%.

The rate of SAE in pediatric patients gradually increased as the CRISP Risk Category increased. Risk Category 5 had a rate of SAE of 66.7% (4/6 procedures); risk category 4 of 12.2% (12/98); risk category 3 rate of 2.9% (8/278); and risk category 2 and 1 had no SAE. For every 1 unit increase in CRISP score in pediatric patients there was a statistically significant increased odds of having a SAE by 1.622 (SE 0.064; *p* value < 0.001).

In adults, patients in risk category 3 had a 5.3% rate of SAE (3/57); risk category 2 a rate of 0% (0/123); and risk category 1 a rate of 0.4% (2/509). For every 1 unit increase in crisp score in adult patients there was a statistically significant increased odds of having a SAE by 1.528 (SE 0.166; *p* value of 0.011). The CRISA scores were also calculated for the adult patients with a SAE and it was found to be more predictive in identifying adults likely to have a SAE than the CRISP.

The mortality rate of this entire cohort of patients was 0.2% which accounted for 8.7% of all SAE (4/29). It should be noted that all deaths occurred in the pediatric age group with 3 of the 4 deaths being in neonates.

Our results have internally validated the utility and accuracy of the CRISP Scoring System in predicting the risk of a SAE both in our pediatric and adult population which both showed that there was a statistically significant increase in the odds of having a SAE for every 1 unit increase in their CRISP Score. We conclude that CRISP Score should be used pre- procedurally and intraprocedurally to risk stratify patients with congenital heart disease undergoing cardiac catheterization.

## 79

### Angiographic Anatomy of Major Aortopulmonary Collateral Arteries and Association with Surgical Outcomes

Gregory Adamson, Doff McElhinney, Yulin Zhang, Jeffrey Feinstein, Lynn Peng, Michael Ma, Claudia Algaze, Frank Hanley, Stanton Perry

Stanford University School of Medicine, Palo Alto, USA

**Abstract**

**Background:** Due to challenges documenting the variability of the pulmonary circulation in patients with tetralogy of Fallot (TOF) and major aortopulmonary collaterals (MAPCAs), the literature on this condition has focused on relatively basic anatomic characteristics.

**Objectives:** To detail the pulmonary artery (PA) and MAPCA anatomy in a large group of infants, to assess relationships between anatomy and surgical outcomes, and to consider systems for classifying MAPCAs.

**Methods:** All infants (age < 1 year) undergoing a first cardiac surgery for TOF/MAPCAs from 2001 to 2019 at Stanford University were identified. Preoperative angiograms that delineated supply to all 18 pulmonary segments were reviewed to assess details of each MAPCA and the arborization and size of central PAs.

**Results:** We studied 276 patients with 1068 MAPCAs and the following PA arborization patterns: 152 (55%) incompletely arborizing PAs, 48 (17%) normally arborizing PAs, 45 (16%) absent PAs, and 31 (11%) unilateral MAPCAs. There was extensive anatomic variability, but there was no difference in surgical outcomes according to PA arborization or the predominance of PAs or MAPCAs. Patents with lower total MAPCA and/or PA cross-sectional area were less likely to undergo complete repair.

**Conclusions:** In TOF/MAPCAs, the distribution of MAPCAs is highly variable and essentially unique for each patient. Though each pulmonary segment can be supplied by a MAPCA, central PA, or both, in our analysis the type of supply was not associated with surgical outcome, and all anatomic combinations were conducive to a good repair. Of the variables we studied, total cross-sectional area of central PA and MAPCA material (TNPAI) was an important driver of outcome, with those having a paucity of total material being the most challenging patients to manage.

**Table:** 

	Total (*n* = 276)	Type 1: Absent central PA (*n* = 45)	Type 2: incompletely arborizing PA (*n* = 152)	Type 3: normally arborizing PA (*n* = 48)	Type 4: PDA or anomalous PA (*n* = 31)	p value
Demographic features						
Female sex	141 (51%)	22 (49%)	72 (47%)	27 (56%)	20 (65%)	0.30
Age (Months)	3.7 (1.0,6.0)	4.7 (2.5,6.4)	4.6 (1.7,6.2)	1.6 (0.1,4.1)	1.3 (0.2,4.4)	<0.001
Weight (kg)	5.2 (3.7,6.5)	5.6 (4.3,6.6)	5.3 (4.0,6.5)	4.2 (3.5,6.2)	4.8 (3.6,5.7)	0.13
PA and MAPCA measurements						
Intrapericardial PA diameter (mm)	2.1 (0.8,3.2)	0 (0,0)	2.6 (1.8,3.9)	2.6 (2.1,3.2)	0.6 (0,1.4)	<0.001
Intrapericardial PA index (mm^2^/m^2^)	29 (4,64)	0 (0, 0)	40 (18,89)	44 (29,70)	4 (0,22)	<0.001
Modified Nakata (mm^2^/m^2^)	56 (16,97)	0 (0,0)	60 (27,105)	63 (43,82)	118 (72,162)	<0.001
MAPCA index (mm^2^/m^2^)	71 (33,129)	154 (119,207)	80 (49,122)	0 (0,39)	35 (20,63)	<0.001
TNPAI (mm^2^/m^2^)	148 (99,195)	154 (119, 207)	165 (111, 204)	80 (51, 116)	165 (121, 225)	<0.001
First surgery						
Complete repair	182 (66%)	34 (76%)	109 (72%)	20 (42%)	19 (61%)	<0.001
Aortopulmonary window	35 (13%)	0 (0%)	10 (7%)	25 (52%)	0 (0%)	
Other palliation	59 (21%)	11 (24%)	33 (22%)	3 (6%)	12 (39%)	
Current status						
Single-stage complete repair	182 (66%)	34 (76%)	109 (72%)	20 (42%)	19 (61%)	<0.001
Multi-stage complete repair	67 (24%)	7 (16%)	27 (18%)	25 (52%)	8 (26%)	
Unrepaired	12 (4%)	1 (2%)	6 (4%)	3 (6%)	2 (6%)	
Death without repair	15 (5%)	3 (7%)	10 (7%)	0 (0%)	2 (6%)	
Complete repair (ever)	249 (90%)	41 (91%)	136 (89%)	45 (94%)	27 (87%)	0.76
RV:Ao at complete repair	0.33 (0.28,0.40)	0.31 (0.27,0.36)	0.33 (0.28,0.40)	0.31 (0.28,0.38)	0.34 (0.25,0.40)	0.40

Data presented as n (%) or median (Q1, Q3). *PA* pulmonary artery, *PDA* ductus arteriosus, *Qp:Qs* ratio of pulmonary to systemic blood flow, *MAPCA* major aortopulmonary collateral artery, *TNPAI* total neo-pulmonary artery index, *RV:Ao* ratio of right ventricle to aortic pressure.

## 80

### Collapse of Thoracic Aortic Covered Stent Following Implantation for Aortic Coarctation Complicate with Infected Thrombus

Ricardo Ortega^1^, Donovan Espriu^1^, Marco Solorzano^2^, Oscar Medina^3^, Gutierrerez Marai^1^, Hayde Estrada^4^, Cristian Villar^1^, Roman Garcia^1^

^1^Departamento de Cardiologia del Centro Medico Nacional del Bajio IMSS T1, Guanajuato, Mexico. ^2^Departamento de Cardiologia/Intervencionismo en Cardiopatias Congenitas del Centro Medico Nacional del Bajio IMSS T1, Guanajuato, Mexico. ^3^Departamento de Cardiologia/Ecocardiografia del Centro Medico Nacional del Bajio IMSS T1, Guanajuato, Mexico. ^4^Departamento de Cardiocirugia del Centro Medico Nacional del Bajio IMSS T1, Guanajuato, Mexico

**Abstract**

**Introduction:** Coarctation of the aorta (COA) occurs in about 6% to 8% of patients with congenital heart disease. The use of covered stents in the treatment of COA has been shown to be safe and effective. This procedure can be associated with complications, artery injury, thrombosis, collapsed stent, aortic injury, endarteritis, restenosis, and death in 0–3.7% only.

**Case Report:** This is a 21-year-old male patient with a significant history of diagnosis of systemic arterial hypertension and post-ductal aortic coarctation both diagnosed at 17 years of age a month later under endovascular treatment with Advanta V12 16 × 61 mm aortic covered stent placement without complications. He began his condition 1 month before admission with unquantified fever, myalgia, arthralgia, and chills with management with multiple antibiotic regimens without improvement. Physical examination revealed heart murmurs with mesosystolic murmur in aortic focus intensity III/VI Levine with irradiation to the neck without s3 s4, limbs with decreased pulses and erythematous lesions, rest of normal physical examination. With studies: Hemoglobin: 8.7 g/dl, Leukocytes 15.8 ul, Creatinine 1.2 mg/dl, and positive blood cultures for streptococcus pyogenes. Transesophageal echocardiogram image of a mixed, irregular, mobile echogenicity mass is observed, with multiple ramifications of approximately 10 × 9 mm on its short axis (Fig. 1), concluding intra-aortic stent with image suggestive of thrombus (Fig. 2). Angiotomography with 3D reconstruction aortic stent in ascending aorta with collapse in the proximal end (Fig. 3) and in sagittal slice image in relation to thrombosis of stent and collapse (Fig. 4). Therefore, it was decided to undergo a surgical emergency treatment to remove a thrombosed stent and place a 10-cm polytetrafluoroethylene graft without complications, culture report of a positive surgical specimen for pyogenes streptococcus.
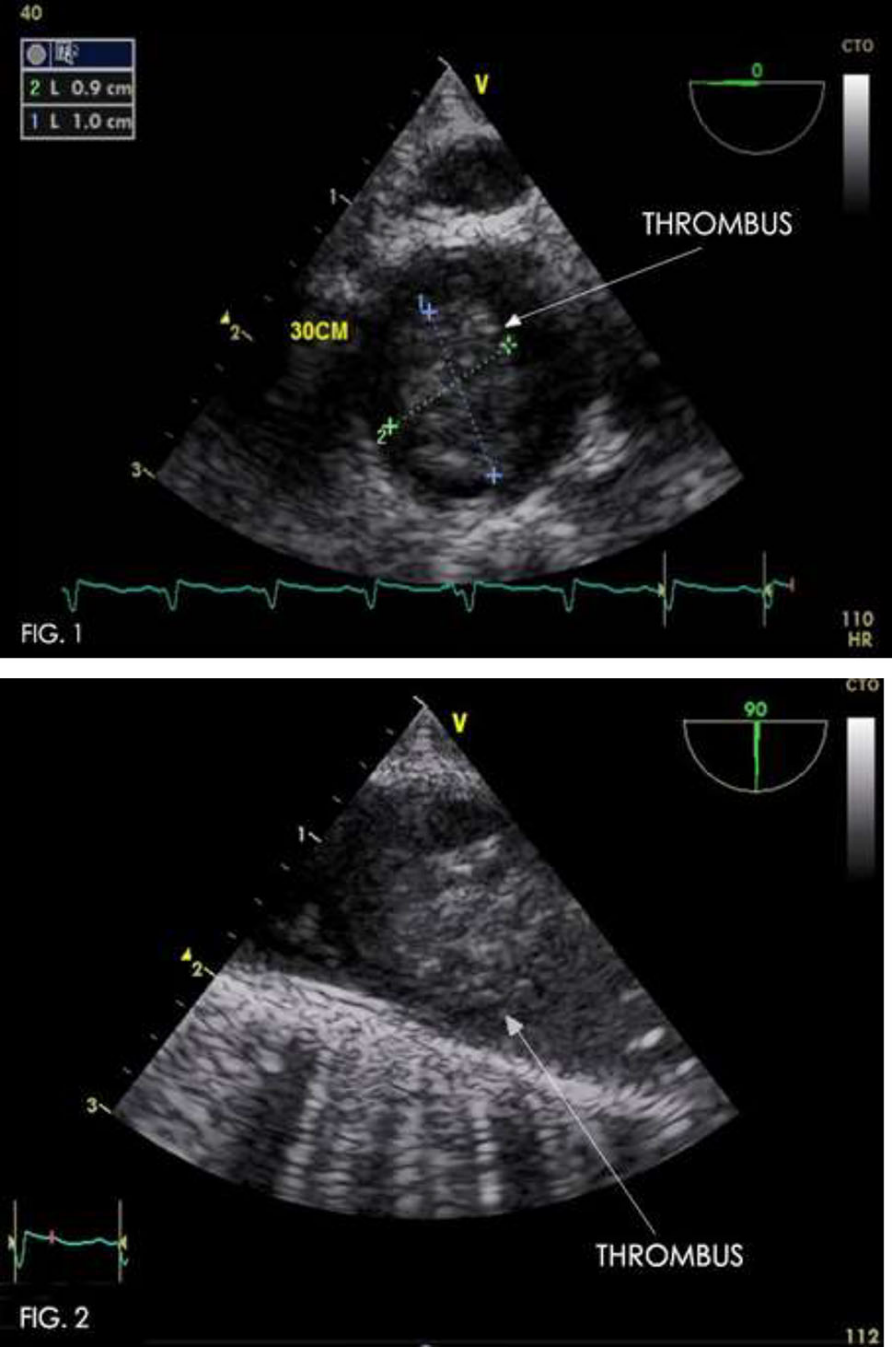

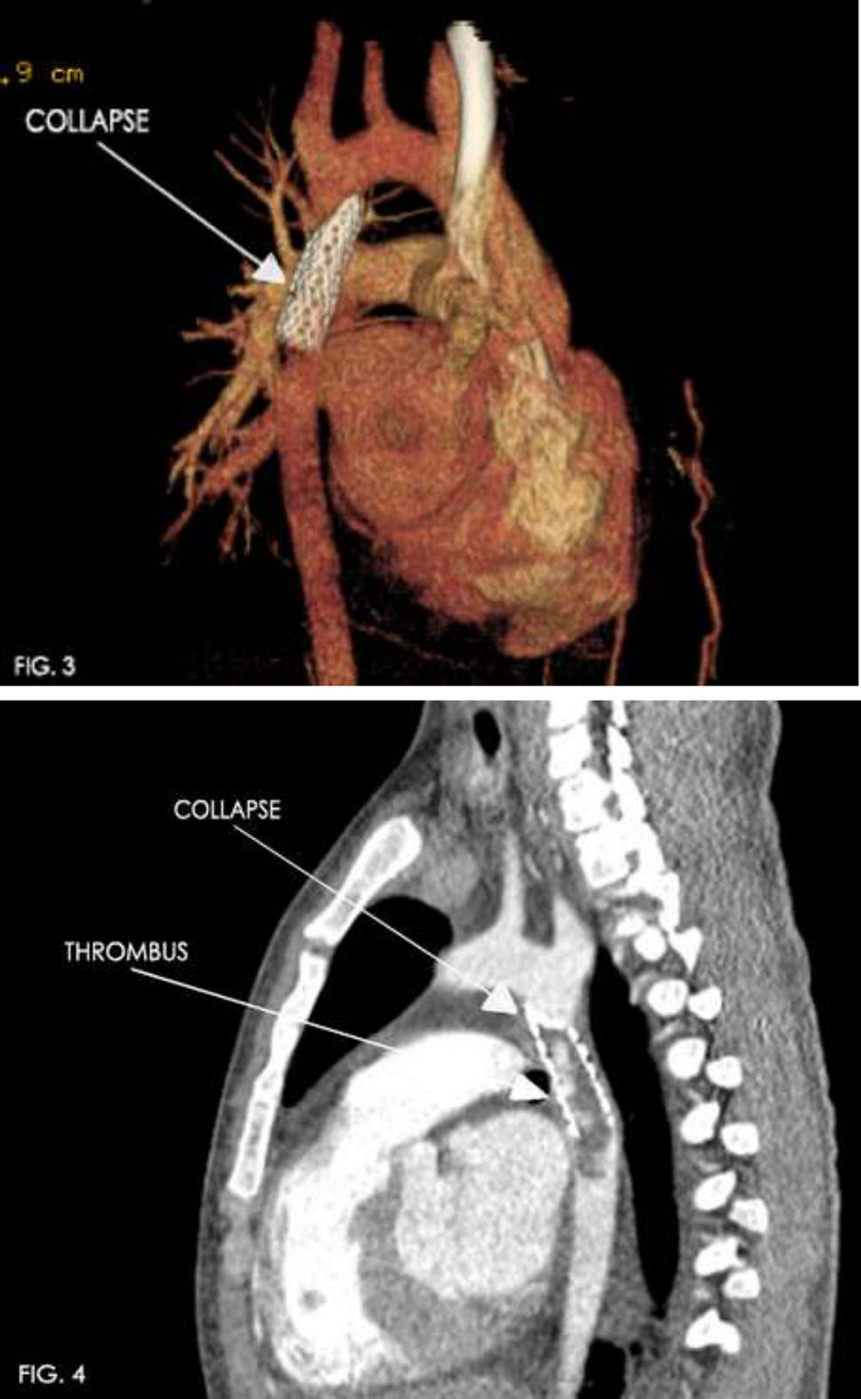


**Justification:** To evaluate a rare complication of infected thrombus of the covered stents used for COA since there are no reported cases in Mexico.

**Discussion:** The use of Advanta covered stent for treatment of aortic coarctation was first reported in 2009, stent is a balloon-expandable laser cut stainless steel open cell design encapsulated. Early reports have shown good short-term results with no acute complications. However, long term following data are not yet available. In this case, the mechanism of collapse of stent proximal was unclear also the complications are generally rare with a low percentage, in our case a collapse of the aortic stent at the proximal level is observed as a complication and what is most striking is the subsequent formation of thrombosis complicated with an infection.

**Conclusion:** The use of stents for aortic coarctation has shown that it is safe, but in the long term it is important to identify its complications, in this case the formation of an infected thrombus secondary to a collapse of the stent is very rare is the only case reported in Mexico.

## 81

### Palliative Ductus Venosus Stenting in a Premature Infant with Obstructed Total Anomalous Pulmonary Venous Connection to the Portal Vein

Renelle George^1,2^, Mohammad Khan^1,2^, John Lozier^1,2^, Martin Bocks^1,2^

^1^UH Rainbow Babies & Children’s Hospital, Cleveland, USA. ^2^Case Western Reserve University School of Medicine, Cleveland, USA

**Abstract**

**Background:** Infradiaphragmatic total anomalous pulmonary venous return (TAPVR) is a subtype of TAPVR in which the four pulmonary veins connect to the umbilicovitelline system, and is usually associated with obstruction. Definitive treatment of TAPVR is surgical repair. We present a case of late-diagnosed obstructed infradiaphragmatic TAPVR which was palliated with stenting of the ductus venosus (DV).

**Case Description:** A 3-week-old female born at 31 weeks of gestation developed new systemic desaturations requiring escalation of respiratory support. Echocardiogram demonstrated infradiaphragmatic TAPVR with a descending vertical vein draining to the portal vein and a small accessory pulmonary vein draining to the left innominate vein. Given that she was clinically stable and weighed only 1775 g, consensus opinion following our case management conference was to defer surgical repair until a minimum of 36 weeks of gestation to allow for improved maturation and weight gain prior to undergoing cardiopulmonary bypass. In the interim, however, she developed *Klebsiella pneumoniae* and worsening hypoxemia which did not respond to 100% FiO2. She became progressively hemodynamically unstable and suffered bradycardic arrest for which she was successfully resuscitated. Echocardiogram demonstrated a newly narrowed DV and new severe pulmonary hypertension (PH). Due to her critical status with guarded neurological prognosis, she was considered too high risk for surgical repair or palliation with ECMO. Therefore, she was emergently brought to the catheterization laboratory for DV stenting to relieve the obstruction. Using ultrasound guidance, transhepatic access was obtained in the portal vein with a 5/4 Merit sheath. Angiogram demonstrated a severely stenotic DV. The DV was crossed using a Maestro microcatheter through a 4 Fr JR 2.5 catheter over a 0.014″ Choice PT wire, which was exchanged for an 0.018″ Steelcore wire. A pre-mounted 4-mm × 16-mm Formula 418 stent was advanced over the wire into the DV, where it was implanted with balloon inflation to 12 atm. Angiogram demonstrated excellent stent placement with vigorous flow into the hepatic venous system and the atria (Figure 1). The balloon, wire, and sheath were removed. Coiling of the transhepatic tract was unsuccessful due to the short distance between liver capsule and the accessed portal vein, so manual compression was performed. Ultrasound demonstrated a small amount of blood in the abdomen, which remained stable on subsequent evaluation.

**Fig. 1:**
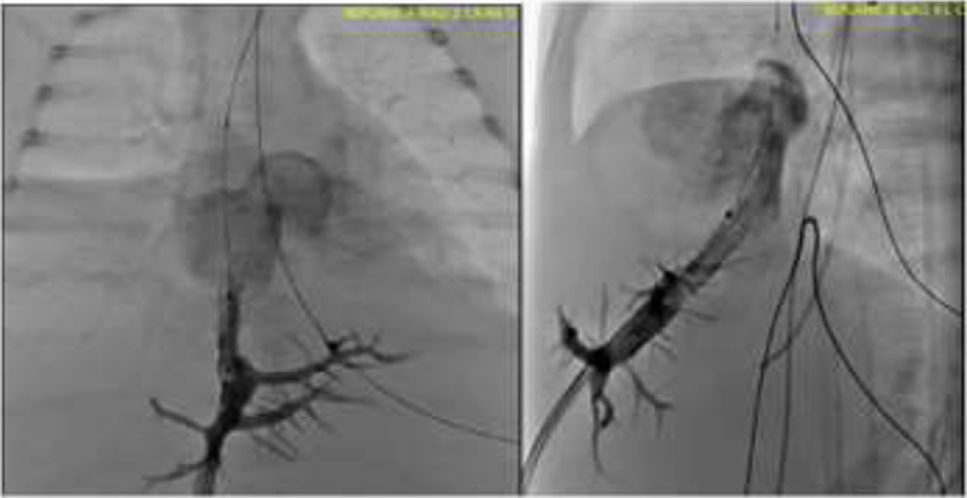
.

The patient tolerated the procedure well with acutely improved oxygenation. Despite adequately decompressing the anomalous venous connection, however, her PH did not abate. Due to her small size and hemodynamic instability, her surgical candidacy did not improve. Her parents elected to withdraw life-sustaining measures.

**Conclusion:** In neonates with obstructed infradiaphragmatic TAPVR, DV stenting may allow time for optimization of clinical status and improvement in surgical candidacy. Although DV intervention for this condition has been described via an indwelling umbilical venous catheter, this is the first report of the procedure via direct transhepatic access. We demonstrate that direct DV stenting is a viable method for short-term palliation in this condition. Early intervention may improve clinical outcomes and allow for relief of obstruction before PH develops.

## 82

### RVOT Stenting in Tricuspid Atresia with Critical Pulmonary Stenosis: Learnings

Neeraj Awasthy^1^, Romilla Chemoriya^2^, Gaurav Kumar^2^

^1^Max Hospital, Saket, Delhi, India. ^2^Max Hospital, Delhi, India

**Abstract**

Tricuspid valve atresia with severe pulmonary stenosis is one of the common cyanotic diseases in neonate. Child can succumb due to profound cyanosis and arterial hypoxemia after closure of PDA. Evolving procedure of Right ventricular outflow tract (RVOT) stenting may be considered as a palliative procedure in such vulnerable group, destined for a later definitive management. The RVOT stenting is described essentially for TOF physiology with a catheter course across tricuspid valve. We describe a case of successful RVOT stenting in 5-day-old symptomatic neonate. We discuss the possible routes and the tips to facilitate RVOT stenting in such a case. This happens to be the first reported case description with successful stenting of neonate with tricuspid atresia with critical pulmonic stenosis.

## 83

### Percutaneous Closure of a Left Ventricular Pseudoaneurysm: Case Report with Review of Cases

Neeraj Awasthy

Max Hospital, Delhi, India

**Abstract**

Percutaneous device closure of a left ventricular (LV) pseudoaneurysm have been rarely reported. We describe the case of a 60-year-old woman with a history closed mitral valvotomy at 18 years of age, Mitral Valve (MV) replacement at 53 year of age in Feb 2016 at 60 year of age. She presented with shortness of breath and chest pain since for 2 weeks. Echocardiography (Echo) revealed LV pseudoaneurysm with neck size of 12 mm. Cardiac MRI showed a large LV pseudoaneurysm (36 × 32 mm) that was filling from a small leak in the anterolateral aspect of the ventricle. Considering high-risk candidate for surgical treatment in view of three previous sternotomies, the pseudoaneurysm was closed percutaneously with use of a 16-mm AMPLATZER muscular VSD occlude successfully. The patient was discharged from the hospital the next day and was asymptomatic on follow-up.

## 84

### A Comparison of Echocardiographic Gradients after Transcatheter Pulmonary Valve Replacement with Melody vs. SAPIEN Valves

Juan Samayoa^1^, Dana Boucek^1^, Elisa McCarthy^1^, Michelle Riley^1^, Lloyd Tani^1^, Arvind Hoskoppal^2^, Robert Gray^1^, Mary Martin^1^

^1^University of Utah, Salt Lake City, USA. ^2^University of Pittsburgh Schools of the Health Sciences, Pittsburgh, USA

**Abstract**

**Introduction:** Transcatheter pulmonary valve replacement (TPVR) has revolutionized the care of patients with congenital heart disease. Two transcatheter valves are FDA approved for TPVR: Medtronic Melody and Edwards SAPIEN. Although small studies suggest higher gradients following SAPIEN TPVR, larger studies comparing echocardiographic outcomes between the two valves are lacking. The aim of this study is to compare the immediate and short-term echocardiographic performance of the Melody and the SAPIEN valves at our institution.

**Methods:** This is a retrospective cohort study of all pediatric patients who underwent TPVR from 2014 to 2019 at our institution. We excluded those in whom implant was not attempted secondary to unfavorable anatomy (coronary compression or lack of landing zone). Echocardiographic parameters (Doppler-derived variables including RV function, peak instantaneous gradient, mean gradient, estimated right ventricular systolic pressure (RVSP), and the presence and severity of pulmonary insufficiency) of patients who underwent TPVR with Melody or SAPIEN valves were compared at three different time points: before implantation, at discharge, and at first follow-up between 1 and 8 months. The Melody and SAPIEN groups were then subdivided and analyzed by right ventricular outflow tract (RVOT) type (native RVOT vs. conduit/bioprosthetic valve).

**Results:** During the study period, 246 patients underwent successful TPVR (Melody *n* = 181, SAPIEN *n* = 65). Groups had similar baseline age, weight, and diagnosis. The most common indications were stenosis (31.5%) or mixed disease (47.5%) in the Melody group and insufficiency in the SAPIEN group (49.2%) (*p* < 0.001). SAPIEN valves were more often placed in native RVOT (60% vs. 17%, *p* < 0.001). The mean final measured valve size on fluoroscopy was 20.9 mm (± 2 mm) in the Melody group and 24.5 mm (± 2.7 mm) in the SAPIEN group. Discharge and follow-up mean and peak Doppler gradients were similar between groups (Table). The patients in the native RVOT group had significantly lower post-implantation gradients in both the Melody and SAPIEN groups (Table). Only 1 patient in the entire cohort (SAPIEN in conduit) had > mild pulmonary regurgitation at follow-up.

**Conclusion:** In our cohort, post-procedure and short-term follow-up Doppler gradients were similar after Melody or SAPIEN TPVR. When groups were subdivided by RVOT type, lower post-procedure gradients were noted after TPVR into a native RVOT. Long-term follow-up is needed to further assess valve function over time.

**Table:** 

	Discharge mean gradient	Discharge peak gradient	Follow-up mean gradient	Follow-up peak gradient
Melody (*n* = 181)	11.7 ± 6.5	22.7 ± 11.4	12.2 ± 7.3	21.6 ± 12.9
SAPIEN (*n* = 65)	10.6 ± 6.1	20.4 ± 11.0	10.1 ± 5.5	18.8 ± 9.4
*P*	0.26	0.19	0.12	0.30
Melody nRVOT (*n* = 30)	7.8 ± 3.7	15.6 ± 6.5	12.0 ± 10	20.9 ± 18.4
Melody Conduit (*n* = 151)	12.5 ± 6.7	24.0 ± 11.6	12.2 ± 6.9	21.8 ± 11.7
*P*	**0.001**	**0.001**	0.29	0.16
SAPIEN nRVOT (*n* = 26)	6.5 ± 3.8	12.5 ± 7.7	6.7 ± 4.2	12.5 ± 6.9
SAPIEN Conduit (*n* = 39)	13.3 ± 5.8	25.5 ± 9.7	12.0 ± 5.3	22.4 ± 8.8
*P*	**0.001**	**0.001**	**0.001**	**0.001**

Mean and peak gradient in mmHg.

## 85

### Endocarditis: Lower Incidence in Native RVOT Transcatheter Pulmonary Valve

Hannah El-Sabrout^1^, Reid Ponder^2^, Arpine Daytyan^1^, Peter Guyon^2^, Ryan Reeves^3^, Kanishka Ratnayaka^1^, John Moore^1^, Jamil Aboulhosn^2^, Daniel Levi^2^, Howaida El-Said^1^

^1^Rady Children’s Hospital San Diego/UCSD, San Diego, USA. ^2^Mattel Children’s Hospital/UCLA, Los Angeles, USA. ^3^Salpizio Medical Center/UCSD, San Diego, USA

**Abstract**

**Background**: The incidence of endocarditis post-transcatheter pulmonary valve (TPV) implantation is variable (2.9–23%). We hypothesized that patients with native right ventricular outflow tracts (RVOT) may have a lower incidence of endocarditis compared to those with non-native RVOT (conduits and bioprosthetic valves).

**Methods**: The institutional databases at Rady Children’s Hospital (UCSD) and Mattel Children’s Hospital (UCLA) were queried for patients receiving TPV over a ten-year period (2010–2020). The incidence of endocarditis for those with native versus non-native RVOT implantation was compared. Kaplan–Meier curves were constructed to compare freedom from endocarditis based on valve type with and without native RVOT.

**Results**: There were 507 TPV, 113 of which had native RVOT and 394 of which had non-native RVOT. Out of these patients, 385 received Melody valves and 122 received Sapien valves. The incidence of endocarditis was 1.8% (*n* = 2) for native versus 9.4% (*n* = 37) for non-native. Of the two native RVOT patients that developed endocarditis, one received a Melody valve, and one received an Edwards Sapien 3 valve. All of the patients who developed endocarditis with non-native RVOT received a Melody valve. The Kaplan–Meier curves below display the incidence of endocarditis between the two RVOT substrates over time, factoring for valve type.
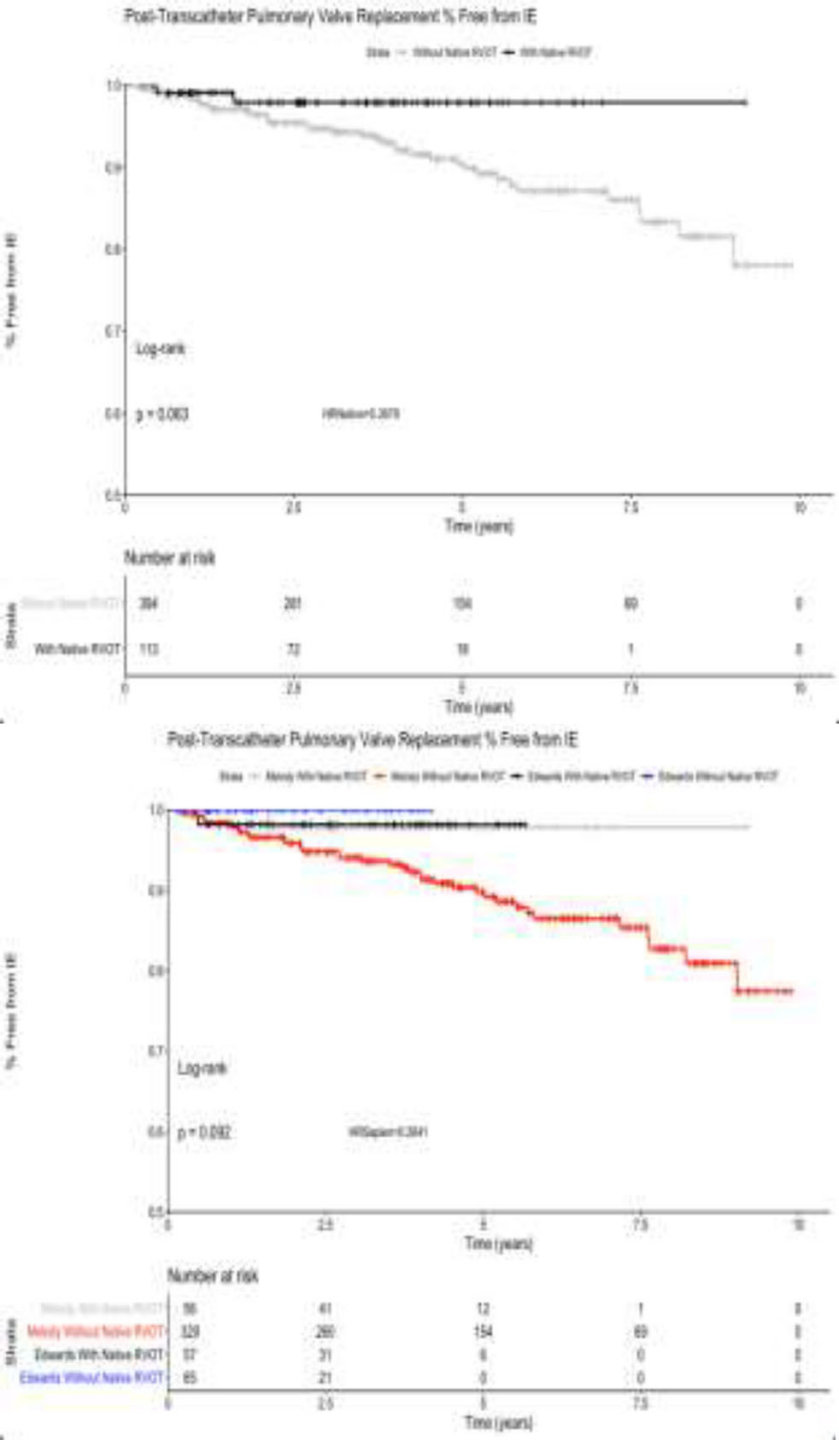


**Conclusion**: The intermediate-term incidence of endocarditis post TPV is low for native RVOT. In this study, there was a trend towards a lower incidence of endocarditis in native versus non-native RVOT. This however did not meet statistical significance (*p* = 0.63).

## 86

### Practice Variation in the Interventional Care of Fontan Patients in the USA

Kiran Mallula^1^, Nathaniel Taggart^2^, Gurumurthy Hiremath^3^, Sarosh Batlivala^4^, Bryan Goldstein^5^, Kurt Schumacher^6^, Jeffrey Zampi^6^

^1^Louisiana State University, New Orleans, USA. ^2^Mayo Clinic, Rochester, USA. ^3^University of Minnesota, Minneapolis, USA. ^4^University of Cincinnati, Cincinnati, USA. ^5^University of Pittsburgh, Pittsburgh, USA. ^6^University of Michigan, Ann Arbor, USA

**Abstract**

**Background:** As with most fields of medicine, there is significant variation in practices in congenital interventional cardiology. Given limited longitudinal data and lack of robust practice guidelines, this is especially notable for the care of patients with single ventricle heart disease after Fontan completion.

**Objective:** To evaluate and describe variations in practice patterns among interventional cardiologists in the United States in the care of patients with Fontan circulation.

**Materials and Methods:** A web-based survey was distributed to congenital interventionists through the PICES and CCISC listservs. Responses were limited to practitioners in the USA. The survey queried providers’ practices regarding diagnostic and interventional catheterizations of Fontan patients and included the role and timing of routine diagnostic catheterizations, ideas of normative hemodynamics, indication for various interventional procedures, and post-intervention anticoagulation management.

**Results:** A total of 100 providers responded. The majority were pediatric interventionists (98%) and 85% worked primarily in a hospital-based academic environment. There was a wide range in center surgical volumes which was fairly evenly distributed between small, medium, and high volumes. While many respondents would perform a diagnostic catheterization for new or worsening symptoms, 72% also perform surveillance diagnostic catheterizations, most typically 5–10 years post Fontan. For hemodynamic variables, most common responses for the upper limits of normal were mean Fontan pressure of 16 mmHg (48%), transpulmonary gradient of 8 mmHg (44%), and systemic ventricular end-diastolic pressure of 10 mmHg (62%). Liver biopsy was routinely performed by only 6% with 57% never performing them. The remainder performed liver biopsy only if clinically indicated, most commonly from a transjugular approach. Pulmonary vasoreactivity testing was fairly common practice (84%), though only routinely performed on all patients by 7%. There was significant variation in the indications to perform interventional procedures for the treatment of branch pulmonary artery stenosis, recurrent coarctation, and aortopulmonary/venovenous collaterals. Similarly, there was a wide variety of indications and contraindications listed for Fontan fenestration closure. In terms of anti-thrombotic therapies, nearly all interventionists use aspirin primarily post-intervention, both short and long-term.

**Conclusion:** While there is wide practice variation in the transcatheter care of Fontan patients especially in indications for interventional procedures, there are some shared views on normative hemodynamics, role for advanced diagnostic maneuvers, and choice of anti-thrombotic therapies among congenital interventional cardiologists. These data suggest a clinical practice guideline may be possible to optimize and standardize outcomes as well as help better evaluate the interventional care provided to patients with Fontan circulation.

## 87

### Comparison Between Patent Ductus Arteriosus Stent and Modified Blalock Taussig Shunt for Neonates and Infant with Ductal-Dependent Pulmonary Circulation in a Small Pediatric Cardiac Surgery Center

Célia Silva^1^, Juliana Taguchi^1^, Gustavo Feitosa^1^, José Aguiar Jr^2^, Jorge Durante^2^, Yan Sasaki^1^, Ana Faccinetto^1^, Renata Mazzini^2^, Camila Silveira^2^

^1^Federal University of São Paulo, São Paulo, Brazil. ^2^General Hospital of Pirajussara, São Paulo, Brazil

**Abstract**

Congenital heart disease (CHD) with ductal-dependent pulmonary circulation are still a challenge because there is necessity of early intervention to maintain the pulmonary flow, either by surgery to have a modified Blalock–Taussig shunt (mBTS) performed or percutaneous by implanting a stent in the ductus arteriosus.

**Objective:** To evaluate the early results of patent ductus arteriosus stent (PDAS) compared to mBTS in patients with ductal-dependent pulmonary circulation in our institution.

**Method:** Retrospective, longitudinal, and unicentric study. Between November 2016 to May 2020, 17 neonates and infants, diagnosed with CHD with a ductal-dependent pulmonary circulation, were admitted in our Hospital. Nine (53%) underwent mBTS surgery (Group 1) and eight (47%) underwent to interventional cardiac catheterization for stent implantation in the ductus arteriosus (Group 2). Gender, age, time of endotracheal intubation and hospital stay, complications, and deaths were assessed.

**Results:** In group 1 (mBTs), the mean age was 44.2 days vs 16.8 days in Group 2. There was male predominance in Group 1 (mBTS)–six (66%), in Group 2 (PDAS), equity of gender were observed. In Group 1 (mBTS), tricuspid atresia with severe pulmonary stenosis was the most common diagnosis with four patients (44.4%), followed by Tetralogy of Fallot (TOF) three (33.3%), and pulmonary atresia with ventricular septal defect and with intact ventricular septum (PA/IVS), with one patient each. In Group 2, the most common diagnosis was PA/IVS with three pts (37.5%), followed by tricuspid atresia with severe pulmonary stenosis—two pts (25%), TOF two pts (25%), and double outlet right ventricle with transposing of great arteries and severe pulmonary stenosis one pt. (12.5%). In comparing Group 1 (mBT) versus Group 2 (PDAS)—average ventilation time was 7.5 days vs 4.8 days, average of hospital stay was 22 vs 13 days. All patients (100%) in group 1 presented complications during hospitalization against 37. 5% in Group 2. The most common complication in both groups was sepsis: in Group 1 5 pts (55.5%) × 2 pts (25%) in Group 2 (PDAS). Extubation laryngitis was seen in one pt. (12.5%) of Group 1. There was no deaths in group 2(PDAS) and there were three (33.3%) deaths in Group 1(mBTS).

**Conclusion:** In our experience, the ductal stent was successful in all cases. There was no crossover to a surgical, no death. Compared with the mBT group, the PDAS group had shorter ventilator days and hospital stay. Our study reinforces that we must continue with strategies to improve ductal stent as an alternative to the surgical shunt

## 88

### Absence of the Presence of Thrombus

Filiz Çelebi, Ocal Karabay

Istanbul Marty Prof. Dr. Ilhan Varank Sancaktepe Research and Training Hospital, Istanbul, Turkey

**Abstract**

A 72-year-old male patient was admitted to the emergency department with a speech disorder and weakness on the right side. In his medical history, he did not have chronic drug use or additional disease except two package/year smoking.

On physical examination, his blood pressure was 142/82 mmHg and his pulse was 72 bmp.

Sinus rhythm and left bundle branch block were seen on the patient’s ECG.

The patient was conscious and cooperative in his neurological examination. He evaluated as right hemiplegic due to loss of strength in his right arm and dysarthric.

Dilated cerebral sulcus secondary to diffuse atrophy and bilateral periventricular chronic ischemic gliotic changes were reported as pathological findings in brain CT.

The patient was admitted to the neurology department with the diagnosis of acute ischemic cerebral vascular event.

In laboratory tests, hemogram, renal, and liver function tests were normal. The patient’s LDL value was measured as 172 mg/dl and HbA1C was measured as 6.4% mmol/mol.

In echocardiographic evaluation, hypertrophy and impairment systolic function of the left ventricle were observed. Ejection fraction was measured as 35% by Simpson’s method.

Carotid vertebral doppler USG was performed for embolic focus etiology. Irregular fibrofatty-calcified plaque was observed extending from the left CCA bulbus level to the left ECA proximal segment. More than 90% stenosis was reported.

In CT angiography, it was stated that this lesion did not cause significant stenosis. Carotid angiography was planned due to conflicting results between imaging methods. In the carotid angiography, thrombus image was detected in the left ICA proximal segment. Carotid endarterectomy was planned for the patient. After the successful endarterectomy operation he was discharged with medical therapy.

PS: CT angiographic and carotid angiographic images of the patient are available if you request we can send.

## 89

### The Phantom Thrombus

Filiz Çelebi, Ocal Karabay

Cardiology Specialist in Istanbul Marty Prof. Dr. Ilhan Varank Sancaktepe Research and Training Hospital, Istanbul, Turkey

**Abstract**

A 72-year-old male patient was admitted to the emergency department with a speech disorder and weakness on the right side. In his medical history, he did not have chronic drug use or additional disease except two package/year smoking.

On physical examination, his blood pressure was 142/82 mmHg and his pulse was 72 bmp.

Sinus rhythm and left bundle branch block were seen on the patient’s ECG.

The patient was conscious and cooperative in his neurological examination. He was evaluated as right hemiplegic due to loss of strength in his right arm and dysarthric.

Dilated cerebral sulcus secondary to diffuse atrophy and bilateral periventricular chronic ischemic gliotic changes were reported as pathological findings in brain CT.

The patient was admitted to the neurology department with the diagnosis of acute ischemic cerebral vascular event.

In laboratory tests, hemogram, renal and liver function tests were normal. The patient’s LDL value was measured as 172 mg/dl and HbA1C was measured as 6.4% mmol/mol.

In echocardiographic evaluation, hypertrophy and impairment systolic function of the left ventricle. Ejection fraction was measured as 35% by Simpson’s method.

Carotid vertebral doppler USG was performed for embolic focus etiology. Irregular fibrofatty-calcified plaque was observed extending from the left CCA bulbus level to the left ECA proximal segment. More than 90% stenosis was reported.

In CT angiography, it was stated that this lesion did not cause significant stenosis. Carotid angiography was planned due to conflicting results between imaging methods. In the carotid angiography, thrombus image was detected in the left ICA proximal segment. Carotid endarterectomy was planned for the patient. After the successful endarterectomy operation he was discharged with medical therapy.

PS: CT angiographic and carotid angiographic images of the patients are available. If you request, we can send.

## 90

### Intramesenteric Lymphatic Imaging in Patients with Congenital Heart Disease

Catherine E. Tomasulo, Yoav Dori, Madhumitha Saravanan, Christopher L. Smith

The Children’s Hospital of Philadelphia, Philadelphia, USA

**Abstract**

**Background:** Intramesenteric (IM) dynamic contrast magnetic resonance lymphangiography (DCMRL) is a novel imaging modality that allows for visualization of mesenteric/intestinal lymphatic drainage. This imaging technique can be used to identify lymphatic flow abnormalities that may not be seen with intranodal (IN) or intrahepatic (IH) DCMRL, especially in patients with protein-losing enteropathy (PLE) and ascites. These findings are essential in planning future interventions, particularly in patients with congenital heart disease (CHD) and high central venous pressure when considering thoracic duct embolization versus selective lymphatic duct embolization and/or thoracic duct decompression.

**Methods:** This is a single-center, retrospective review including all patients with CHD who underwent IM-DCMRL from 5/2019 to 3/2020.

**Results:** Twenty-five patients with CHD underwent IM/IH/IN-DCMRL at median age 12.3 years (range 0.2–37.1 years) and median weight 30 kg (range 3.4–111.4 kg). Twenty patients had single ventricle heart disease: 18 s/p Fontan, one s/p Glenn, and one s/p heart transplantation. The remaining five patients had transposition of the great arteries s/p arterial switch operation, pulmonary atresia s/p biventricular repair, total anomalous venous return s/p repair, coarctation of the aorta s/p repair with dysplastic mitral valve, and pulmonary stenosis s/p pulmonary valvotomy.

17/25 patients had a prior diagnosis of PLE. 16/17 had duodenal perfusion or intralumenal leak identified with both IH and IM-DCMRL. One patient had no abnormal duodenal perfusion with IM, but distal duodenal perfusion with IH-DCMRL. 11/16 patients had leak into the proximal duodenum with both injections and five had perfusion or leak into the distal duodenum with IM-DCMRL and leak into the proximal duodenum with IH-DCMRL. Seven of the remaining eight patients without a clinical PLE diagnosis had no abnormal leak from IM or IH-DCMRL. However, one patient showed a duodenal leak only with IH-DCMRL. The ability of IM-DCMRL and IH-DCMRL to identify duodenal perfusion and intraluminal leaks correlating with clinical PLE was statistically significant (*p* < 0.001). No patient had duodenal leak/perfusion identified with IN-DCMRL.

17/25 patients were also noted to have ascites. DCMRL imaging identified a peritoneal leak in 12/17; one with only IM-DCMRL, three with only IH-DCMRL, and eight with both. Two patients without prior ascites had peritoneal leaks identified; one only with IH-DCMRL and another with IN-DCMRL, IH-DCMRL, and IM-DCMRL. The ability of IM-DCMRL and IH-DCMRL to identify peritoneal leaks in patients with ascites was statistically significant (*p* < 0.05).

There were no procedural complications or mortality. Twelve patients underwent subsequent selective glue embolization of abnormal hepatoduodenal/periduodenal lymphatic networks identified and two underwent glue embolization of lymphatic networks leaking into the peritoneum.

**Conclusion:** IM-DCMRL is an effective imaging modality for evaluating mesenteric lymphatics and can help identify abnormalities in lymphatic flow not seen on other imaging, particularly in patients with PLE or ascites. This information can help with the planning of future procedures for patients with CHD.

## 91

### Stretched to the Limit: Comparing PTFE-Covered Endovascular Stents Through Serial Dilations

Ernesto Mejia, Emily C. Kish, Martin L. Bocks, John S. Lozier

UH Rainbow Babies and Children’s Hospital, Cleveland, USA

**Abstract**

**Introduction:** Covered stents are often needed during congenital cardiac catheterization to treat high-grade stenoses or to repair/exclude areas of injured vasculature following angioplasty or bare-metal stenting. Several small diameter covered stents are commercially available and have been used off-label in children. The ability to post-dilate a stented vessel to eventual adult diameters to keep pace with somatic growth is critical following initial stent placement in children. The ability to post-dilate a covered stent is potentially more limited by its PTFE covering. This study aimed to compare in vitro performance characteristics of two brands of covered stents with serial post-dilation and fracture.

**Methods:** Seven iCast stents (Atrium Medical Corporation, Hudson, NH) and eight Viabhan VBX stents (W. L. Gore & Associates, Inc., Newark, DE) of various sizes were measured before expansion using a Keyence digital microscope (Keyence Corporation of America, Ithaca, IL). The stents were then expanded with their pre-mounted balloons, followed by measurements of stent diameter and length both with the balloon inflated and deflated. Subsequent serial dilation’s were performed using non-compliant balloons (Conquest or Atlas [Bard Peripheral Vascular, Inc, Tempe, AZ]) at 2-mm-diameter intervals ranging from 6 to 22 mm, with additional measurements made after each expansion. Balloon diameter and pressure were recorded at stent fracture, PTFE tear, and ‘napkin-ring’ formation.

**Results:** The VBX stents fractured in a predictable manner. Stent fracture of the 5-mm and 6-mm diameter VBX stents occurred during dilation with the 10-mm balloon (100% and 67% oversized, respectively); the 7-mm VBX stents fractured on the 14-mm balloon (100% oversized); and the largest VBX stents fractured on the 20- or 22-mm balloon (~ 180% oversized). In comparison, all of the iCast stents fractured during dilation with the 14- or 16-mm balloons, and had less predictable fracture patterns (range 75–220% oversizing). The PTFE of the VBX stents tore only with stent fracture or later. All of the iCast stents experienced partial or complete PTFE tear prior to stent fracture, however, these stents were studied after their expiration dates. Both stents had similar recoil between 5.5 and 5.8%. The VBX stents experienced significantly more foreshortening and achieved a ‘napkin-ring’ configuration more readily than the iCast stents.

**Conclusion:** Compared to Atrium iCast stents, Gore Viabhan VBX endovascular stents had more foreshortening and similar recoil during serial post-dilations. VBX stents fractured at predictable post-dilation diameters based on their nominal diameter. The VBX stents readily developed a ‘napkin-ring’ configuration, and their PTFE tore after stent fracture in all cases. The iCast stents fractured at 14 or 16 mm diameter, irrespective of original diameter, and all experienced PTFE tear prior to stent fracture. These in vitro performance characteristics should be considered when selecting covered stents in small children with congenital heart defects and when considering post-dilation at the time of implantation or during a catheterization procedure performed in the future.

## 92

### Clinical Utility of Cardiac MRI for Prediction of Balloon-Expandable Percutaneous Pulmonary Valve Implantation in Patients with a Non-Conduit Right Ventricular Outflow Tract

Brian Boe^1^, Aimee Armstrong^1^, Darren Berman^1^, Sharon Cheatham^1^, John Cheatham^2^, Kan Hor^1^

^1^Nationwide Children’s Hospital, Columbus, USA. ^2^Nationwide Childrens Hospital, Columbus, USA

**Abstract**

**Background:** Balloon-expandable (BE) percutaneous pulmonary valve implantation (PPVI) is an effective treatment for patients with non-conduit right ventricular outflow tract (RVOT) dysfunction. Most patients undergo advanced imaging prior to BE PPVI. We sought to correlate the RVOT measurements from cardiac magnetic resonance imaging (cMRI) with the gold standard of balloon-sizing prior to BE PPVI and how well cMRI predicts successful implantation.

**Methods:** All patients who underwent a cMRI within one year prior to balloon-sizing for BE PPVI were evaluated in a single center. The RVOT was retrospectively measured at the intended landing zone on both the cMRI and cardiac catheterization in a blinded fashion. The cardiac catheterization measurements included systolic and diastolic angiographic measurements, diameter of the waist during the balloon-sizing, and final diameter of the BE PPVI. Measurements were compared using Spearman’s correlation.

**Results:** From 2012 to 2018, 33 patients met study inclusion criteria. Patients were a median (range) age of 19.4 (10.6–62.1) years and weight of 60 (32.1–134) kg. The majority of patients were born with tetralogy of Fallot (*n* = 20) and had undergone a surgical repair with a transannular patch (*n* = 23) at a median (range) time of 18.5 (9.4–51.2) years prior to attempted PPVI. Indication for PPVI included pulmonary regurgitation (*n* = 26) and mixed RVOT disease containing stenosis and regurgitation (*n* = 7). PPVI was successful in 24 patients using the available BE TPVs implanted at the following diameters: 20 mm (*n* = 1); 22 mm (*n* = 8); 23 mm (*n* = 2); 26 mm (*n* = 6); 29 mm (*n* = 7). Nine procedures were unsuccessful secondary to large RVOT size (*n* = 3), coronary/aortic root compression (*n* = 4), and inability to technically implant the valve (*n* = 2). There was moderate correlation between the RVOT diameter by cMRI of 23.4 ± 4.6 mm (mean ± standard deviation) compared to the balloon-sizing waist of 23.5 ± 2.3 mm, with a Spearman’s correlation coefficient of 0.53. Based on cMRI data, the imaging team predicted 24/24 (100%) of successful BE PPVI. The imaging team predicted 4/9 (44%) unsuccessful procedures which included the three patients with a RVOT too large for BE PPVI.

**Conclusions:** There is moderate correlation between the dynamic RVOT by cMRI and balloon-sizing as it pertains to BE PPVI. A dedicated PPVI team can help predict successful implantation using cMRI data. Further study into other variables such as RVOT shape, RVOT length, and dynamic change in diameter throughout the cardiac cycle will lead to improved understanding and prediction of PPVI in the non-conduit RVOT.

## 93

### Melody Endocarditis Does Not Equal Valve Removal: Variations in Practice in a Multicenter Experience

Arpine Davtyan^1^, Peter Guyon^1^, Hannah El-Sabrout^2^, Reid Ponder^2^, John Bradley^1^, Caitlin Heyden^1^, Linda Drake^3^, Kanishka Ratnayaka^1^, John Moore^1^, Asimina Courelli^4^, Bryan Mosher^1^, Holly Bauser-Heaton^3^, Daniel Levy^2^, Jamil Aboulhosn^2^, Henri Justino^5^, Howaida El-Said^1^

^1^UC San Diego/Rady Children’s Hospital, San Diego, USA. ^2^UCLA, Los Angeles, USA. ^3^Emory/Children’s Healthcare Atlanta, Atlanta, USA. ^4^UC San Diego School of Medicine, San Diego, USA. ^5^Texas Children’s Hospital, Houston, USA

**Abstract**

**Background:** There is growing concern regarding the incidence of infective endocarditis (IE) after placement of Melody Valves (MV). The percentage of patients with MV endocarditis requiring surgical removal and/or transcatheter intervention have been reported to be as high as 52% with mortality related to the valve in up to 8.7% of cases. We aimed to better characterize cases of MV endocarditis which could safely and adequately be treated with antibiotic therapy and not require surgical removal.

**Methods:** All cases of endocarditis in patients with MV from four centers (Rady-UCSD, Mattel-UCLA, Children’s Healthcare Atlanta/Emory and Texas Children’s Hospital) were reviewed. Cases that were successfully treated medically without reoccurrence and cases that necessitated surgical removal of the valve were compared in order to identify factors associated with successful medical treatment.

**Results:** From 2010 to 2019, 666 Melody valves were implanted with total cumulative incidence of 63 cases of endocarditis in 61 patients (9%). Endocarditis was successfully treated medically in 33 (54%) of patients without reoccurrence during a mean duration of follow-up of 2.9 years (range 4.5 months to 9.5 years) after treatment. Two patients treated medically(6%) had reoccurrence of IE: one patient required removal of the valve with the second episode; the other patient was successfully treated without recurrence during follow-up of over 2 years. Infection with streptococcal species, no vegetations visualized on echocardiography and lower residual RVOT gradient at time of valve placement were associated with successful medical treatment. The most common reasons for valve removal were valve stenosis (28.6%), concern for inadequate response to antibiotics(persistent elevation of inflammatory markers, fevers, inability to clear blood cultures, etc.; 14.3%), valve stenosis with RV dysfunction (10.7%), and surgeon preference (7.1%). The proportion of cases of IE that were treated with surgical removal of the MV at each institution varied (27% vs. 42% vs. 55% vs. 64%).

**Conclusion:** In a large multicenter study, MV IE managed with antibiotic therapy alone was successful in the majority of cases, with streptococcal IE, absence of vegetations on echocardiography, and lower residual MV gradient at time of valve placement bring predictive of successful antibiotic therapy. There was noticeable institutional variation in the proportion of cases of IE that were treated with surgical removal of the MV.

## 94

### Transcatheter Closure of Patent Ductus Arteriosus in Premature Infants—How Young is Too Young?

Abhishek Chakraborty^1^, Ranjit Philip^1^, Ronak Naik^1^, Mimily Harsono^2^, Huma Abdulmajeed^1^, Rush Waller^1^, Shyam Sathanandam^1^

^1^Division of Pediatric Cardiology, University of Tennessee Health Sciences Center, LeBonheur Children’s Hospital, Memphis, USA. ^2^Division of Neonatology, University of Tennessee Health Sciences Center, LeBonheur Children’s Hospital, Memphis, USA

**Abstract**

**Introduction**: Transcatheter patent ductus arteriosus (PDA) closure (TCPC) has been shown to be feasible and safe for infants as small as 700 g or even smaller. However, the corrected gestational age of the infant may be a better representation of tissue maturity than the weight of the infant. Therefore, this study was aimed to explore the outcomes of TCPC based not just on weight, but also on the age of the infant.

**Methods**: This was a retrospective study including all infants born at gestational age < 28 weeks who underwent TCPC between Jan 2012 and May 2020 and weighed < 3 kg at the time of the procedure. The study period was divided into four time blocks, Jan 2012 to Dec 2014, Jan 2015 to Dec 2017, Jan 2018 to July 2019, and Aug 2019 to May 2020. Success and complication rates were compared for those who underwent procedure at < 28 days to those infants that underwent TCPC > 28 days of age.

**Results**: During the study period, TCPC was attempted on 241 infants that satisfied the inclusion criteria, with 99.1% success rate. There was no difference in success rates based on the age at TCPC (99.3% vs. 99% for procedural age < 28 days vs. > 28 days, respectively; P = 0.82). Overall, 140 infants underwent TCPC at < 28 days of age, 29 of whom were < 14 days, and 54 infants underwent TCPC between 15 and 21 days of age. The median age at TCPC progressively decreased from 60 days of life (14–211 days) between January 2012 and December 2014 to 18 days (8–38 days) between August 2019 and May 2020 (P < 0.00001). Similarly, the weight at TCPC decreased over the same time periods (median 1985 g vs. 900 g; *P* < 0.00001). Procedure-related complication rates were similar for those < 28 days vs. > 28 days of age at the time of TCPC (2.8% vs. 4.9% respectively; *P* = 0.42).

**Conclusions**: It is feasible and safe to perform TCPC after the first week of life for infants born < 28 weeks’ gestation. As experience grows, more infants are referred earlier after birth which translates to performing TCPC on smaller infants. Benefits of early TCPC have to be determined in order for this therapy to be advocated routinely for all premature infants (Table 1).

**Table 1:** .

Time period	Overall (Jan 2012–May 2020) *N* = 241	Jan 2012–Dec 2014 *N* = 44	Jan 2015–Dec 2017 *N* = 82	Jan 2018–Jul 2019 *N* = 72	Aug 2019–May 2020 *N* = 43	*p* value
Procedure age (days)						
Mean ± SD	38 ± 33.6	76 ± 53.7	35 ± 18.6	31 ± 21.4	19 ± 7.6	< 0.00001
Median (range)	28 (8–211)	60 (14–211)	28 (14–140)	27 (9–145)	18 (8–38)	
Procedure weight (g)						
Mean ± SD	1229 ± 528	1872 ± 546	1161 ± 419	1054 ± 420	992 ± 308	< 0.00001
Median (range)	1000 (500–3000)	1985 (745–3000)	1000 (640–2400)	900 (580–2600)	900 (500–1600)	

## 95

### Safety and Efficacy of Veno-Arterial Extracorporeal Membrane Oxygenation Cannulation by Interventional Cardiologist During Cardio-Pulmonary Resuscitation in Children

Abhishek Chakraborty^1^, Cihangir Buyukgoz^2^, Hitesh Sandhu^2^, Samir Shah^2^, Shyam Sathanandam^1^

^1^Division of Pediatric Cardiology, University of Tennessee Health Sciences Center and LeBonheur Children’s Hospital, Memphis, USA. ^2^Division of Pediatric Critical Care Medicine, University of Tennessee Health Sciences Center and LeBonheur Children’s Hospital, Memphis, USA

**Abstract**

**Introduction**: Veno-arterial extracorporeal membrane oxygenation (VA ECMO), a form of temporary mechanical circulatory support can be used as a bridge to recovery, to more durable bridge or to definitive treatment. As ECMO technology evolves, alternative cannulation strategies are being developed. We assessed a single institution experience on ultrasound-guided percutaneous VA-ECMO cannulation performed at the bedside by interventional cardiologist.

**Methods**: Pediatric patients (< 18 years old) who underwent for VA-ECMO cannulation by interventional cardiologist from January 2019 to June 2020 in a tertiary care children’s hospital were reviewed retrospectively. Children cannulated by pediatric surgery or cardiovascular surgery alone excluded.

**Results**: Six patients were included, of which three were female. One of the patients was cannulated twice. Three patients had history of congenital heart disease. Mean age was 13.5 (6–18) years. Mean weight was 68 (17–109) kg. Five patients (71%) were cannulated due to cardiogenic shock and two (29%) patients were cannulated due to cardiorespiratory arrest. Three patients (43%) were cannulated from right femoral vein, right internal jugular vein, and left femoral artery; whereas four patients (57%) were cannulated from right femoral vein and left femoral artery. Median venous cannula size was 21 (15–25) French and median arterial cannula size was 17 (15–17) French. Mean cannulation start time to ECMO flow time was 45 (18–112) min. No cannula repositioning or replacement required. 30-day survival and survival at ECMO decannulation were 86% (*n* = 6). One patient (14%) developed ECMO cannulation-related complication. No patient had loss of limb circulation. Mean ECMO duration was 3.7 (0.3–8) days. Cardiovascular surgeon was present during all ECMO cannulations.

**Conclusion**: Ultrasound-guided percutaneous VA-ECMO cannulation performed at bedside by interventional cardiologist is safe and feasible in pediatric population. However, larger studies and further investigations are necessary.

## 96

### Feasibility and Safety of Transcatheter Closure of Patent Ductus Arteriosus in Infants Weighing < 700 Grams

Abhishek Chakraborty^1^, Ranjit Philip^1^, Ronak Naik^1^, Rush Waller^1^, Mimily Harsono^2^, Shyam Sathanandam^1^

^1^Division of Pediatric Cardiology, University of Tennessee Health Sciences Center and LeBonheur Children’s Hospital, Memphis, USA. ^2^Division of Neonatology, University of Tennessee Health Sciences Center and LeBonheur Children’s Hospital, Memphis, USA

**Abstract**

**Introduction**: Recently, the Amplatzer Piccolo Occluder (APO) was approved for PDA closure for infants weighing > 700 g. The feasibility and safety of the procedure for anyone below this weight cut-off has not been previously explored. The aim of the study is to describe the feasibility and safety transcatheter closure of patent ductus arteriosus (PDA) in premature infants who weigh < 700 g at the time of the procedure.

**Methods**: This is a retrospective, single-center, study to explore the outcomes of transcatheter PDA occlusions performed on infants weighing < 700 g.

**Results**: A total of 18 patients weighing between 540 and 670 g (mean = 612.8 g) were identified to have underwent the procedure between the two centers. The gestational age ranged between 22 and 26 weeks (median 24 weeks), and average birth weight was 537.5 g. The mean age at the time of PDA closure was 20 days (range 8–35 days). The implant success rate was 100%. The devices used included the MVP-5Q (N = 8), the 3-2 APO (N = 5), and the 4-2 APO (N = 5). The procedure time was 26 ± 23 min and fluoroscopy time was 3.7 ± 3.1 min. One patient, weighing 640 g required chest compressions for resuscitation following the procedure, but recovered and since then has grown normally to hospital discharge. There were no other procedural complications. At latest follow-up (median 1-year), there have been four non-survivors, all unrelated to the procedure.

**Conclusions**: It is feasible and likely safe to perform transcatheter closure of patent ductus arteriosus (PDA) in premature infants who weigh < 700 g using currently available technologies. There is a learning curve with these interventions. Extreme care must be taken while performing interventions in such small human beings. Further miniaturization of equipment would facilitate better outcomes.

## 97

### Extremely Rare Anomaly of the Systemic Drainage Associated to Anomaly of the Pulmonar Venous Drainage

Gustavo Santos, Juliana Taguchi, Ana Carolina Faccinetto, Yan Sasaki, Harold Barretto, Aislan Pinheiro, José Cícero Guilhen, João Saba, Celia Silva

UNIFESP, São Paulo, Brazil

**Abstract**

**Introduction**: Normal systemic venous drainage occurs through the venous connection of the superior and inferior vena cava and coronary sinus draining into a morphologically right atrium. The most common anomaly of systemic venous drainage that have been described is the persistence of the left vena cava draining into the coronary sinus. However, anomalous drainage of the right superior vena cava into the left atrium is extremely rare, and few cases has been reported in the literature. Cyanosis, heart failure, and brain abscess are the most common forms of clinical presentation.

**Case Report**: Ten-years-old girl, previously healthy, with a history of short breathless and cyanosis for 2 years ago after an episode of brain abscess. Upon physical examination, with a resting oxygen saturation of 88% in room air, with light digital clubbing, and mild cyanosis. A 2D transthoracic echocardiogram showed a positive microbubble test, with a large amount of saline contrast into the left chambers, despite the interatrial septum appear to be intact. Subsequently, the patient underwent to a CT for further investigation, which showed a possible drainage of the right superior vena cava into the left atrium and a patent foramen ovale (PFO).

She was then referred for a cardiac catheterization for better evaluation and surgical plan. The diagnosis of the superior vena cava (SVC) draining into the left atrium was confirmed, in addition an anomalous drainage of the right upper pulmonary vein into the SVC was seen. The patient was then sent for surgery. At the surgery the distal portion of the right SVC with the right upper pulmonary vein was left in place and the remaining portion was then anastomosed to the right atrium. The PFO was closed. Her postoperative period was uneventful, with the oxygen saturation of 98% in room air. She was discharged on oral anticoagulation and has been followed up on the outpatient clinic.

**Conclusion:** Anomalous drainage of the superior vena cava in the left atrium is extremely rare, as the lesion causes an obliged right-to-left shunt, surgical repair is necessary. It can be isolated or associated to anomalous pulmonary venous drainage and interatrial communication. Cyanosis, heart failure, and brain abscess should draw attention for the possibility of this diagnosis.

**Figure:**
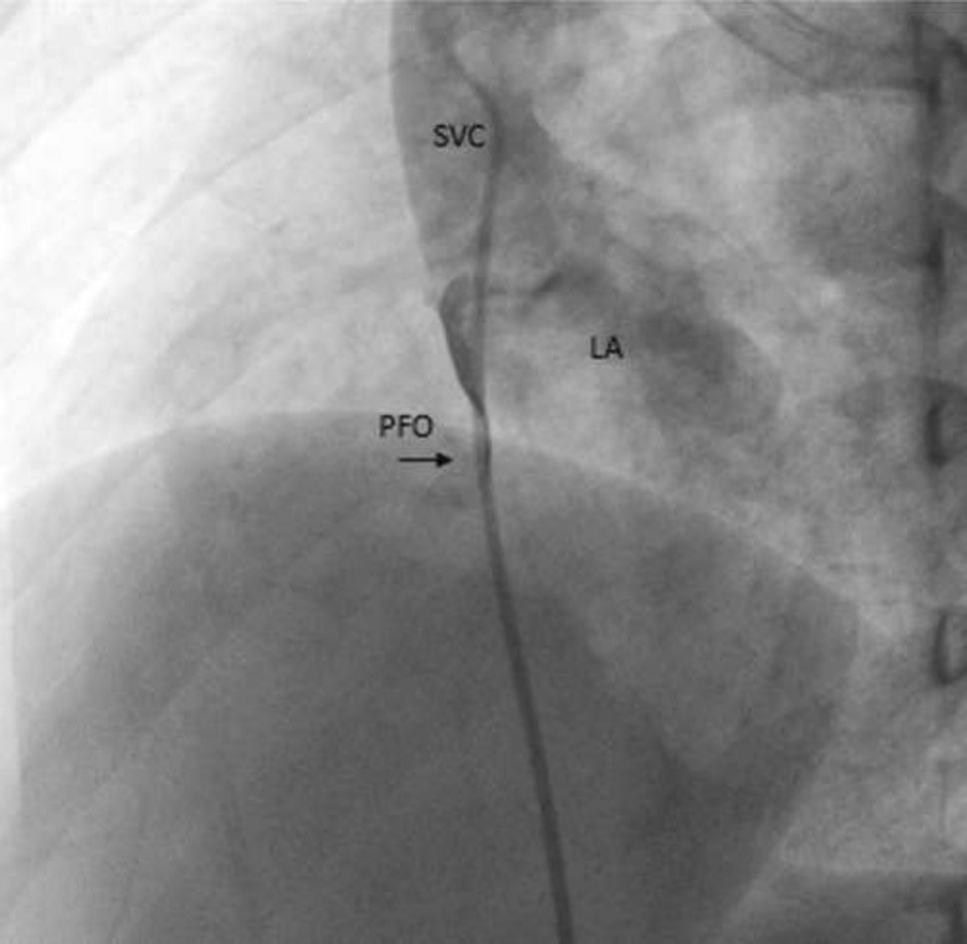
**Figure 1**

**Figure:**
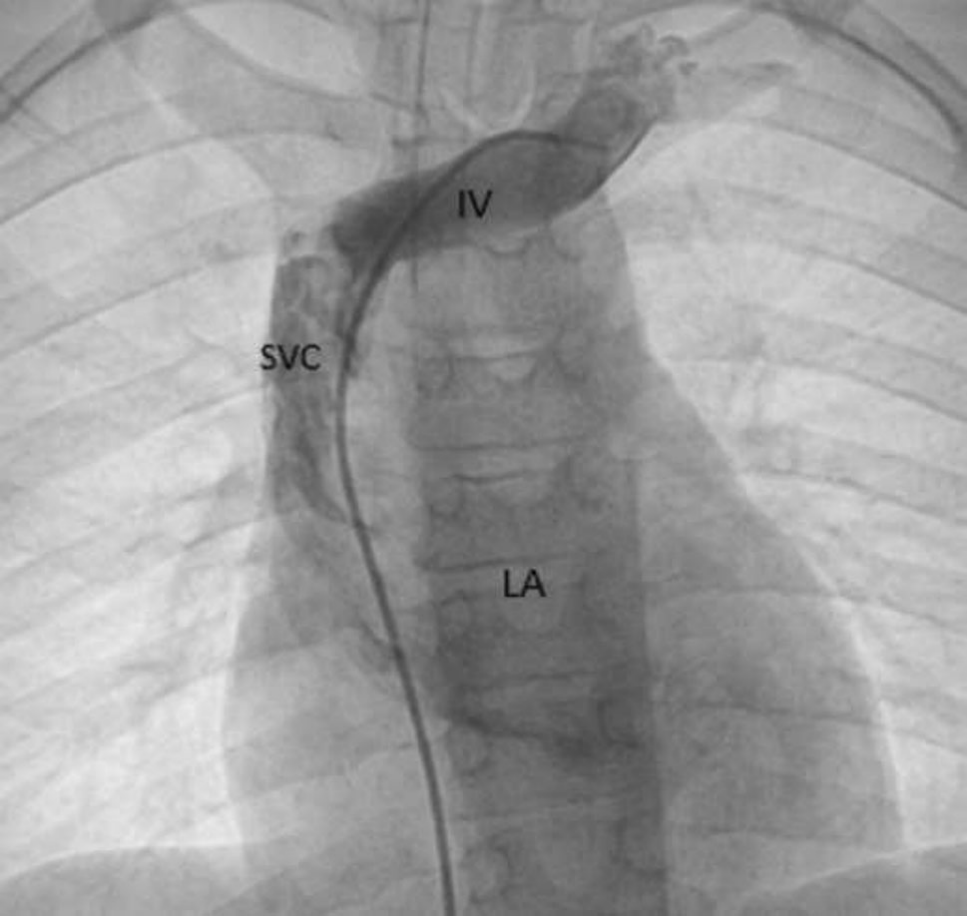
**Figure 2**

**Figure:**
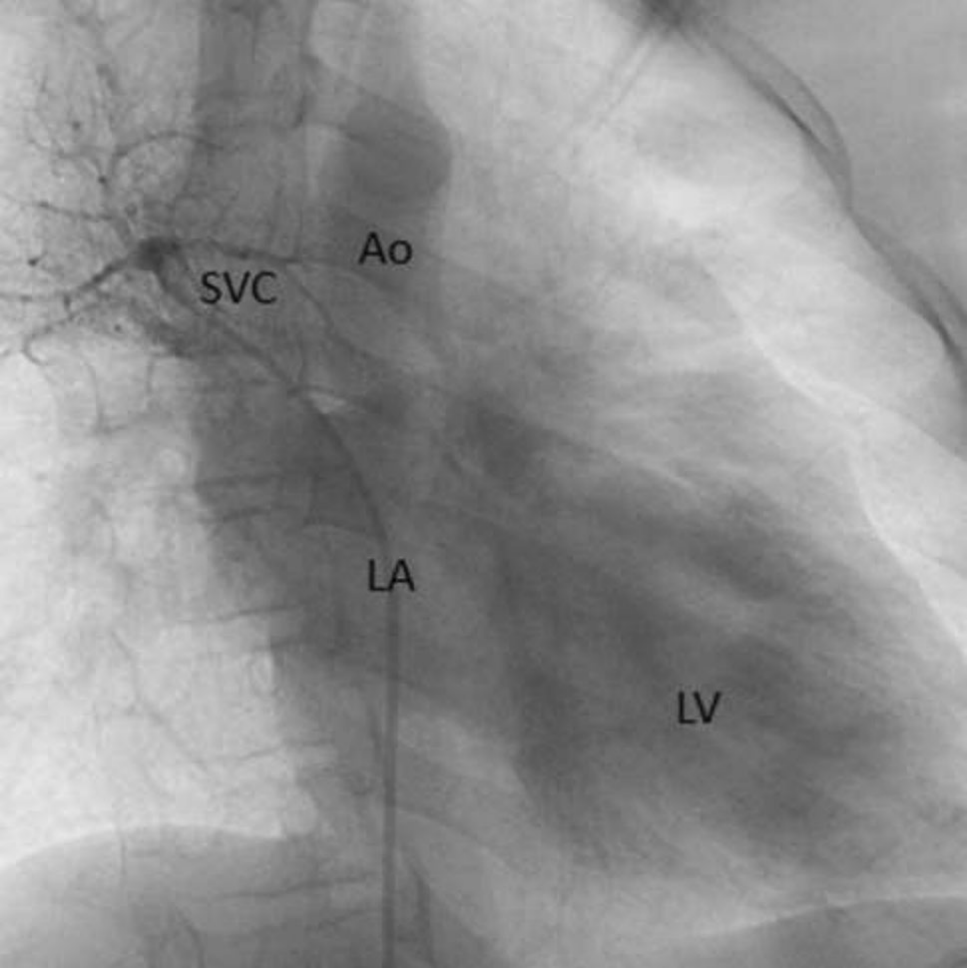
**Figure 3**

**Figure:**
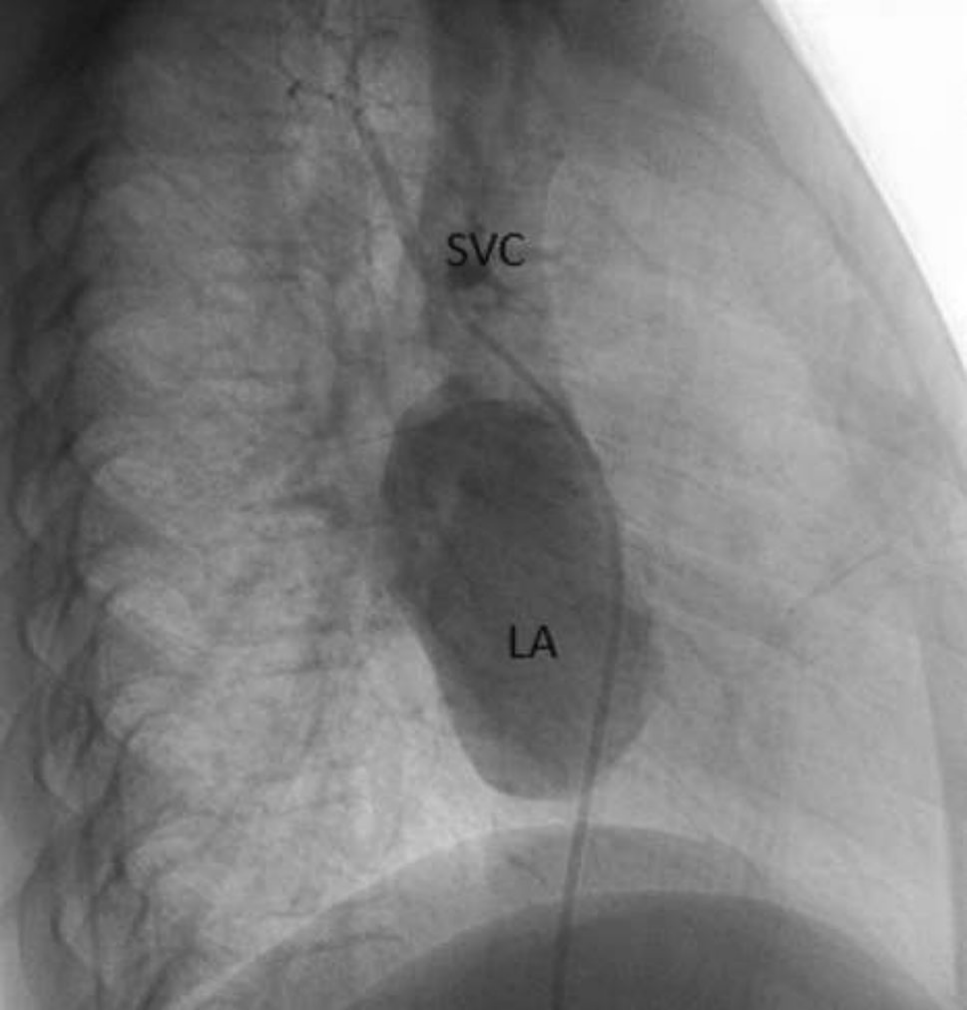
**Figure 4**

## 98

### Stent Implantation in a Ductus Arteriosus in a New-Born with the Presence of the 5th Aortic Arch and Complex Heart Disease with Anomaly of the Basal Vessels

Ana Carolina Faccinetto, Juliana Taguchi, Gustavo Santos, João Saba, Celia Silva

UNIFESP, São Paulo, Brazil

**Abstract**

**Introduction:** The persistence of the fifth arch arteries is extremely rare. The bilateral fifth arch arteries have never been documented, unilateral partial fifth arch artery has been demonstrated only once in a developing human embryo, dorsal collateral channels connecting the fourth and sixth arches are common, to qualify as the fifth arch artery any channel must arise from the ascending aorta proximal to the brachiocephalic artery, take a serpentine course and lie extrapericardially and terminate in the dorsal aorta, or through the sixth arch into pulmonary artery or arteries. The use of echocardiography for diagnosis alone is difficult, with percutaneous angiography (or both) considered as reference methods. However, cardiac computed tomography and magnetic resonance imaging can more accurately outline the anatomy of the arch and its variation. Case report: Male neonate 12 days old, born in another service, term, first child, mother with non-insulin-dependent gestational diabetes, presented with choking and cyanosis on the first day of life, being referred to a neonatal intensive care unit, where, after hearing a heart murmur, he performed echocardiography and was diagnosed with tricuspid atresia and pulmonary valve stenosis. Maintained with prostaglandin and referred for evaluation by pediatrics cardiology and therapeutic procedure. The echocardiogram confirmed tricuspid atresia, pulmonary valve stenosis, and aortic arch to the right. Cardiac catheterization has been programed for stent placement in the ductus arteriosus. During examination, previous diagnoses were confirmed and anomalous origin of the vessels in the base was observed. There was a right subclavian with usual origin and other vessels originating from a common trunk (5th arch) that emitted the right and left carotid arteries, left subclavian, and tortuous artery canal (Figure 1). Through a puncture through the left axillary artery (Figure 2), two coronary stents were positioned, the first Nexgen 3.5 × 19 mm in the distal segment, and the second Nexgen 4.0 × 13 mm in the proximal segment of the canal, in overlapping nails with the first. Procedure performed successfully and without complications (Figure 3). The patient evolved with 89% saturation, discharge from the ICU on the 2nd postoperative day, and referred for outpatient follow-up using anti-aggregation. Conclusion: The presence of the 5th arch artery is rare and when associated with other complex heart diseases, it needs the evaluation of physicians specialized in congenital heart diseases for the proper diagnosis and treatment.

**Fig. 1:**
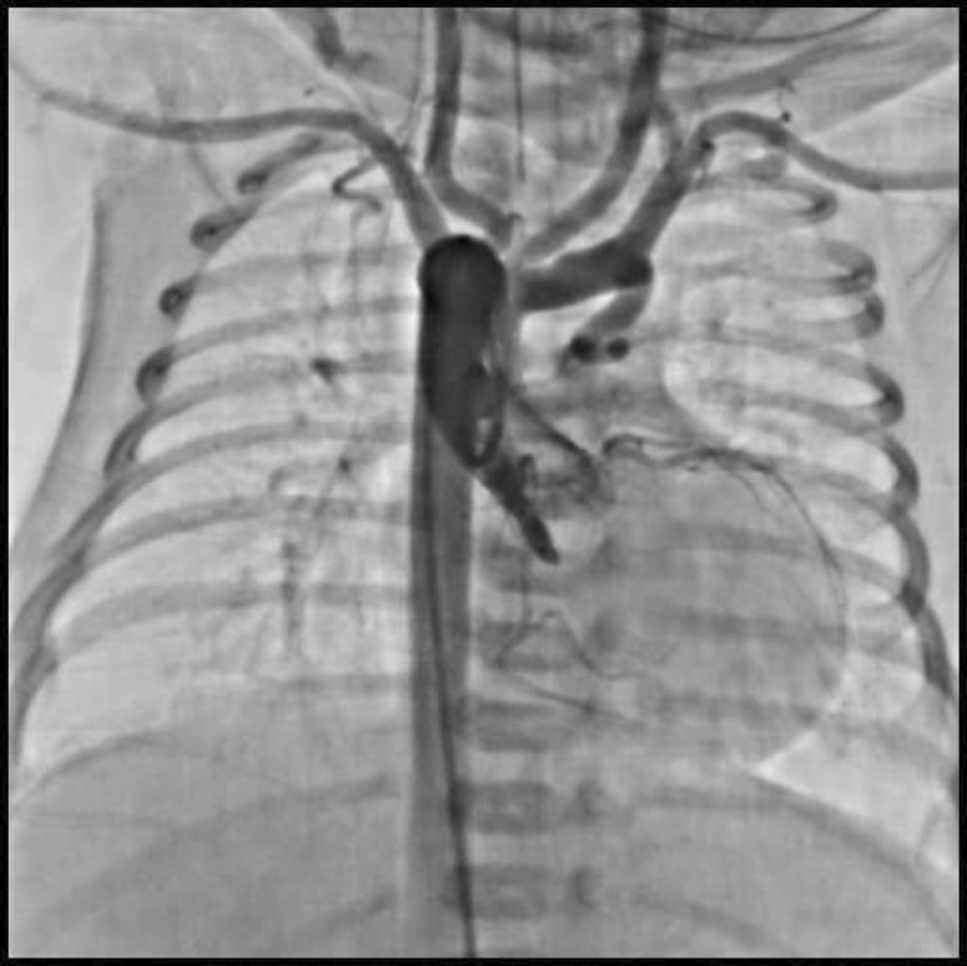
.

**Fig. 2:**
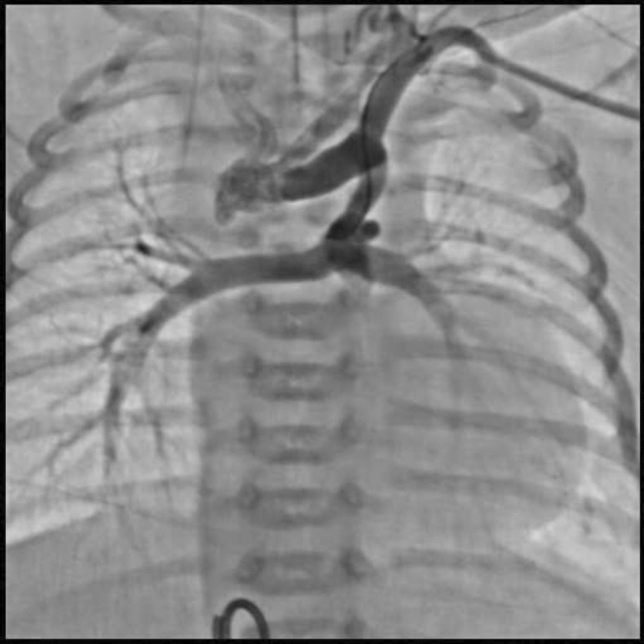
.

**Fig. 3:**
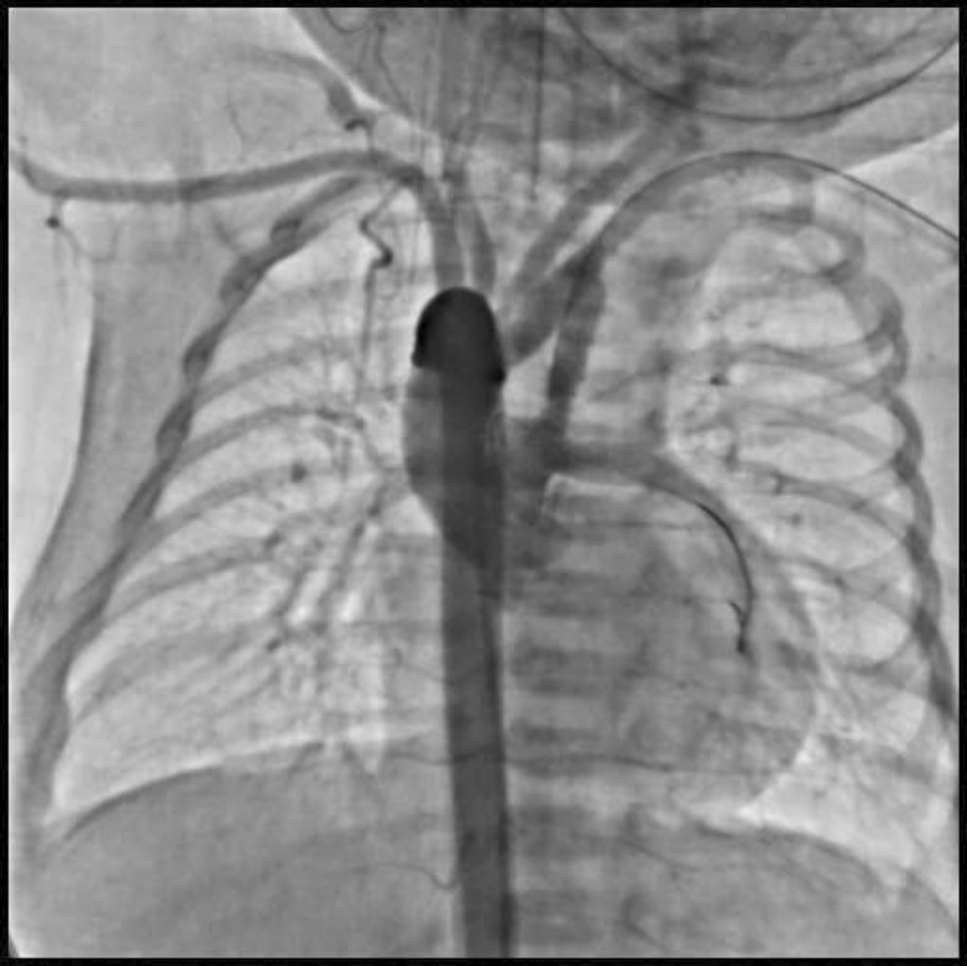
.

## 99

### Transcatheter vs Surgical Closure of Ostium Secundum Atrial Septal Defects: Trends and Comparison of Outcomes from 2003–2016 in Children in the United States

Krishna Kishore Umapathi^1^, Aravind Thavamani^2^, Joshua Murphy^1^

^1^Rush University, Chicago, USA. ^2^Rainbow Babies and Children’s Hospital, Cleveland, USA

**Abstract**

**Introduction:** Transcatheter (TC) closure of atrial septal defects (ASD) has become the gold standard modality in adults. However, small size and defective aortic rims in children make TC technically challenging and surgical closure (SC) might be preferred. The possibility of device erosion led to a 2012 FDA panel review for use of the Amplatzer Septal Occluder device that might have limited its use. We sought to investigate the trends of ASD closure using both modalities and its effect on healthcare utilization in the United States.

**Methodology:** We analyzed the National Inpatient Sample and Kid Inpatient Database from 2003 to 2016. Two cohorts, TC and SC, were created and compared to study the outcomes and temporal trends in healthcare usage in children (< 20 years) with ostium secundum ASD. Patients with complex congenital heart disease, other congenital anomalies, and genetic disorders were excluded.

**Results:** A decreasing trend in the use of TC for ASD was noted with a noticeable drop in TC-ASD since 2011–2012 (Figure 1). SC was more common in the younger cohort (1–4 years), males and Caucasians (all *p* < 0.001)—Table 1. While hemopericardium, infective endocarditis, and ventricular arrhythmias were significantly more common in SC (all, *p* < 0.001), arterial thrombus, stroke, device embolization (all, *p* < 0.001), and severe sepsis (*p* = 0.01) were significantly more common in TC. No significant differences were noted in cardiac tamponade, complete heart block, cardiac arrest, sepsis, and death between the groups. Among procedures, blood transfusion, cardiopulmonary resuscitation (both *p* < 0.001), and pacemaker insertion (*p* = 0.013) were significantly associated with SC with no significant difference noted in ECMO use between the groups (Table 2). Mean length of stay (LOS) and mean hospitalization charges (HC) are significantly higher in the SC group (3.93 ± 0.04 vs 1.87 ± 0.06 and 90,421 ± 835 vs 62728 ± 1200, both *p* < 0.001). Mean inflation-adjusted HC and LOS show an overall uptrend in both groups during the study period (Figure 2).

**Figure:**
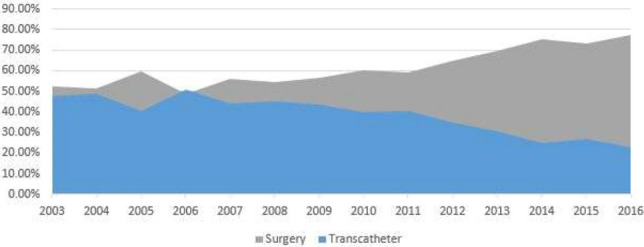
Overall trends in surgical and transcatheter closure of ASD during the study period

**Figure:**
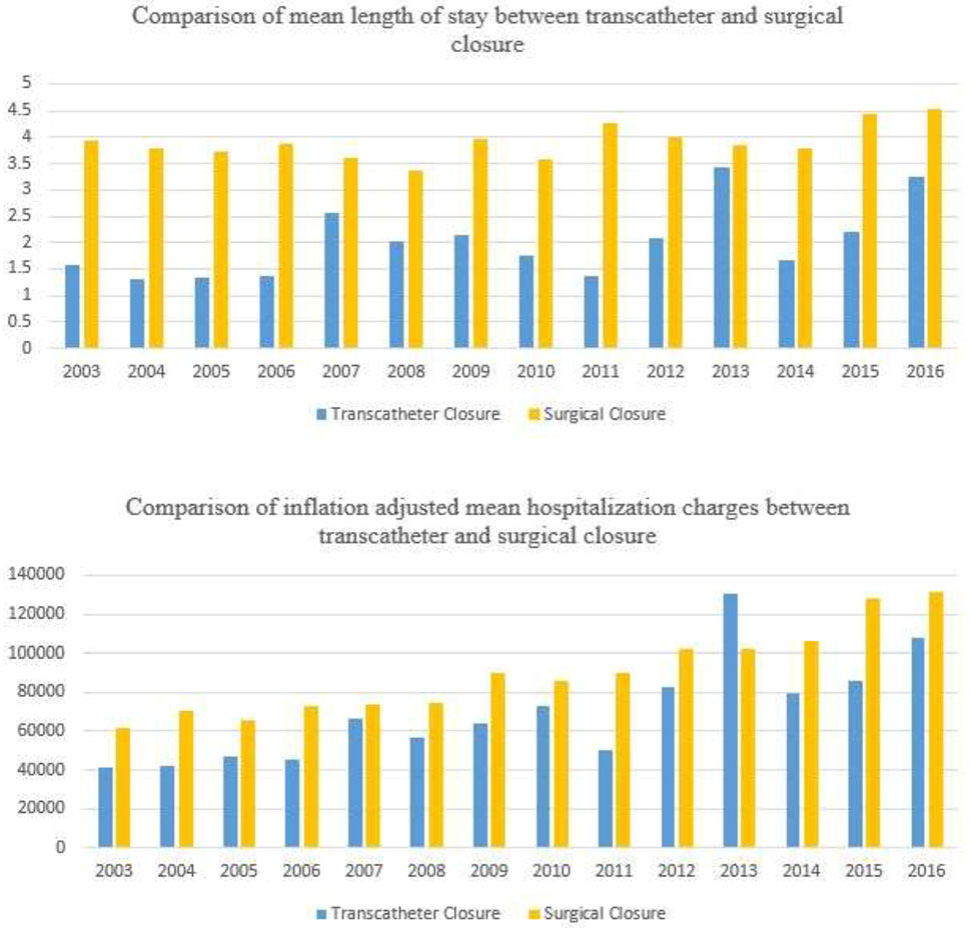
Trends of mean length of stay and related hospitalization charges over the study period

**Figure:**
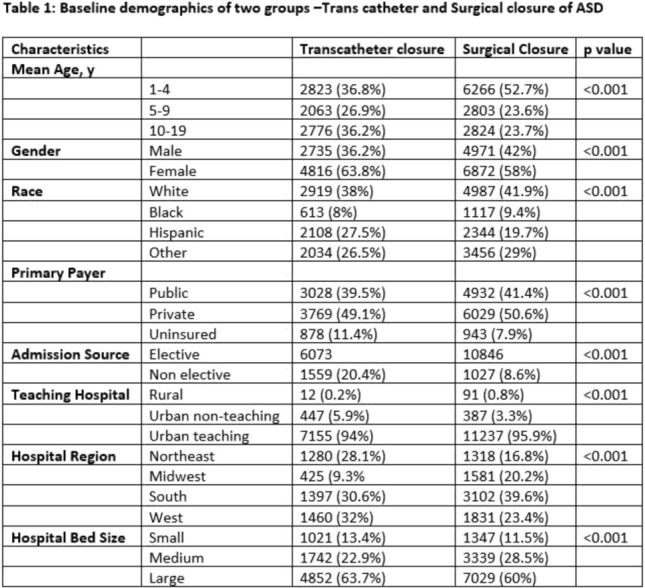
Baseline demographics of two groups—transcatheter and surgical closure of ASD

**Figure:**
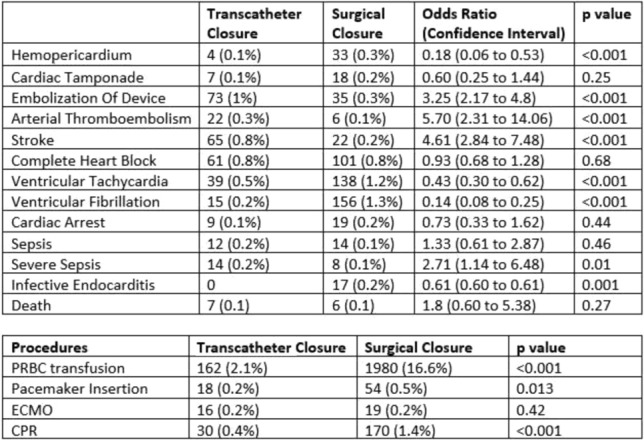
Comparison of comorbidities and complications between SC and TC ASD

**Conclusion:** There is a notable overall national trend favoring SC-ASD in children causing an impact on increasing healthcare resource use. Further studies are needed to study practice variations that are prevalent due to uncertainty in choosing between the two modalities of treatment to better help improve outcomes in ASD.

## 100

### Clinical Case: Inflammatory Multisystem Syndrome in Minor Infant with Giant Aneurisms of Coronary and Abdominal Aorta

Dr. Martha Elizabeth Rubio Hernandez^1^, Dr Carlos Escobedo Uribe^1^, Dr Luis Fernando Perez Gonzalez perez^1^, Dr Mario Martinez^1^, dra Veronica vasquez^2^

^1^Hospital Dr Ignacio Morones Prieto, San Luis Potosi, Mexico. ^2^Hospital Especialidades Jose Carrasco Arteaga, Cuenca, Ecuador

**Abstract**

Three-month-old female infant, who was admitted for 40-degree fever of 10 days of evolution, in poor general irritability, with 5 days of diarrheal stools, was treated with antipyretic and antimicrobial with no improvement on the two recent days vomiting, irritability, rejection of the oral route, with a punctate rash, erythematosus, in the chest, upper limbs as well as dry cough, dehydration, for which reason he entered the pediatric acute respiratory care unit in isolation at the risk of infection by SARCOV-2 due to suspicion.

Upon admission to the hospital, the patient had leukocytosis, anemia, decreased platelets and altered liver enzymes.

During her hospitalization with fever of 40 degrees of difficult handling, systematic desaturation of 50–60%. in paraclinics with elevated Ferritin 569, low hemoglobin 5.7 g, platelets 70,000, elevation of dimer D: 2.3.

Chest thorax with apical right infiltrate, light cardiomegaly.

Heart echocardiogram without congenital anatomical malformations, light pericardial effusion, mitral regurgitation, and light tricuspid, LVEF 65% Simpson, TAPSE 12 mm, PSAP 25 mmHg, both right dilated coronaries with rosary image measuring 2.5 mm in origin and 5 × aneurysm 7 mm diameter. Left coronary artery with an 8-mm-diameter aneurysm.

EKG sinus rhythm without data on ischemia or arrhythmia, Troponin I 0.001.

Immunoglobulin was started at 2 g/kg/dose to pass in 24 to 36 h, aspirin 80 mg/kg/day, continued with antibiotic.

Infant Febrile persists, 2 peaks of 38 and 39.2 C despite 72 h of antibiotic and immunoglobulin dose.

It was decided to administer a second bolus of immunoglobulin at 2 g/kg/dose, continue ASA and clopidogrel, and Methylprednisolone bolus of 30 mg/kg for 3 days.

Without improvement with persistent fever, a second dose of immunoglobulin was administered.

With good response to the afebrile patient treatment, without data of respiratory distress, tolerates the oral route, is adequately fed.

An angiotomography was performed, showing giant aneurysmal lesions in both coronaries, aneurysms in the left subclavian artery, mammary arteries, and a large aneurysm in the abdominal aorta.

At discharge, the patient was on enoxaparin 5 mg every 12 h, aspirin 5 mg/kg/day, Clopidogrel 0.2 mg/kg/day, prednisone, and weekly methotrexate.

**Conclusion**: From the studies of Dr Yamasaki in Mexico in 2012 and 2018, it was established that in some patients they can develop toxic shock due to kawsaki disease, however, due to the presence of covid-19, in children with negative pcr but with a positive or igg test for covid-19, develop a very aggressive presentation that can lead to involvement of large-caliber arteries such as the abdominal aorta. The genomic profile is pending.

## 101

### Patent Ductus Arteriosus Catheterization Closure in Preterm Infants: A Descriptive Study of an Institutional Experience

Heidi Kim^1^, Sushmita Yallapragada^1^, Rashmin Savani^1^, Vedanta Dariya^1^, Sana Ullah^1^, Kate Mangona^1^, Timothy Pirolli^1^, Larry Brown^2^, Surendranath Reddy^1^

^1^University of Texas Southwestern Medical Center, Dallas, USA. ^2^Parkland Health and Hospital System, Dallas, USA

**Abstract**

**Introduction**: A patent ductus arteriosus (PDA) in a preterm infant can lead to respiratory insufficiency and chronic lung disease as well as necrotizing enterocolitis due to systemic hypoperfusion. Recently, catheter-based PDA closure has emerged as an appealing alternative to invasive surgical ligation in preterm infants; however, evidence to support this procedure in infancy, particularly in the premature population, is still evolving.

**Methods**: A retrospective chart review was conducted to collect information regarding demographics, neonatal and maternal clinical characteristics, and procedural and post-catheterization data. Inclusion criteria included infants born at < 32 weeks of gestational age whose PDAs were closed via catheterization at our institution between January 2017 and August 2019. Infants with critical congenital heart disease and/or lethal congenital anomalies were excluded from this study.

**Results**: Twenty-seven patients were included in our study. Indications for PDA closure in our patient population included hemodynamically significant PDA on echocardiogram, difficulty weaning respiratory support, poor feeding and weight gain, and development of NEC. Median gestational age and weight were 26 3–7 weeks and 820 g (ranging from 23 3–7 to 30 weeks and 400 to 1540 g, respectively). Most infants tolerated the procedure well and without serious adverse outcomes. One infant developed an intimal flap post catheterization that resolved without invasive intervention, and 1 infant developed a PAH crisis after PDA closure with resolution of PAH prior to discharge. Statistically significant associations were found between PDA plug closures and decreased oxygen requirements, decreased pulmonary artery hypertension, decreased diuretic requirements, and decreased vasodilator requirements by time of discharge.

**Conclusions**: Successful device closures of PDAs are feasible and safe, and preliminary findings show promising outcomes without mortality or long-term morbidity following transcatheter PDA closure.

## 102

### Right Ventricular Outflow Tract Stenting in Symptomatic Infants Without the Use of a Long Delivery Sheath

Niall Linnane, Mohamed Nasef, Colin J McMahon, Orla Franklin, Terence Prendiville, Mark Walsh, Adam James, Paul Oslizlok, Kevin P Walsh, Damien Kenny

Children’s Health Ireland Crumlin, Dublin, Ireland

**Abstract**

**Introduction:** Right ventricular outflow tract (RVOT) stenting has been shown to improve pulmonary artery blood flow and pulmonary artery growth in symptomatic infants with tetralogy of Fallot, prior to complete repair. The stent is typically delivered to the RVOT using a long delivery sheath. The aim of this study was to describe the safety and efficacy of RVOT stenting without the use of a long delivery sheath.

**Methods:** Retrospective data analysis of patients under 1-year old undergoing RVOT stenting from January 2010 to January 2020 at a single tertiary pediatric cardiology center. Patient demographics and follow-up data are provided. Data are presented as medians with range in parentheses.

**Results:** Sixty-seven RVOT stents were deployed during 56 procedures into 47 patients (tetralogy of Fallot (*n* = 25), atrioventricular septal defects and tetralogy of fallot (*n* = 8), double outlet right ventricle (*n* = 5), and other conditions with right ventricular outflow tract obstruction (*n* = 9). The median age and weight at insertion were 41 days (range 2–204) and 3.67 kg (range 1.59–7.1), respectively. Thirty-one procedures were semi-elective and 25 were emergencies. Stent positioning was confirmed with transthoracic echocardiogram and/or RV angiography across the VSD from an aortic approach. The median total procedure and fluoroscopy times were 67.5 (range 15–145) and 19 min (1–107), respectively. The median radiation dose was 136cGY/cm^2^ (range 5–2002). Thirty-five patients were either admitted in ICU pre-procedure or required ICU admission post procedure with a median ICU length of stay of 3 days (range 1–48). The median length of stay was 8 days (range 1–258). There were 3 major complications and 2 minor complications. There were two deaths (included in the 3 major complications) within 30 days of the procedure. A patient with Cornelia de Lange Syndrome (1.8 kg) died following stent embolization and inability to wean from emergency cardiopulmonary bypass for stent retrieval and the second infant had an unexplained asystole arrest post procedure while awaiting transfer to ICU.

**Conclusion:** RVOT stenting has become commonplace for treating severe RVOTO in small symptomatic infants in the setting of a VSD. In this series, we demonstrate that the procedure is technically possible with minimal complications without the use of a long sheath.

## 103

### Early Outcomes for Patent Ductus Arteriosus Stenting in Neonates ≤ 2.5 kg with Duct-Dependent Pulmonary Circulation

Mohamed Al-Nasef^1,2,3^, Doaa Shahbah^4^, Sarosh Batlivala^5^, Colm R Breatnach^1^, Nial Linnane^1^, Kevin P. Walsh^1^, Paul Oslizlok^1^, Brian McCrossan^1^, Athar M Qureshi^4^, Bryan H. Goldestein^5^, Damien Kenny^1^

^1^Children Health Ireland, Dublin, Ireland. ^2^Prince Sultan Cardiac Center, Riyadh, Saudi Arabia. ^3^Salmaniya Medical Complex, Manama, Bahrain. ^4^Texas Heart Institute, Houston, USA. ^5^Cincinnati Children Hospital, Cincinnati, USA

**Abstract**

Patent Ductus Arteriosus (PDA) stenting has evolved as a valid alternative to surgical systemic-pulmonary shunts in patients with duct-dependent pulmonary circulation facilitating improved saturations and pulmonary artery growth. This may be more compelling for neonates ≤ 2.5 kg, in whom surgical palliation may be challenging. The objective of this study is to evaluate outcomes following PDA stenting in patients ≤ 2.5 kg from three large tertiary centers.

**Methods:** Retrospective review of all neonates ≤ 2.5 kg with duct-dependent pulmonary circulation who underwent stenting from three tertiary referral centers. Procedural details, pulmonary arterial growth, reinterventions, surgery type, and outcome were assessed.

**Results:** Forty-two PDA stents were implanted in a total of 31 patients (16 female) at median age of 8 days (IQR 5–15) and median weight of 2.1 kg (IQR 2–2.4 kg). Thirteen patients (41%) had femoral artery access, 8 patients (25%) had carotid artery access, and the remaining had axillary or umbilical cord artery access. Two patients developed femoral artery thrombosis, while one patient developed right carotid artery aneurysm, and another had intracranial thrombosis. Twenty-one patients (68%) had tortuosity indices of II or III. Oxygen saturations improved from median of 73% (IQR 62.5–79.5) to 88% (IQR 80–93%, *p* < 0.001). Two patients proceeded to emergency surgical shunts and one patient underwent a surgical shunt 74 days post PDA stenting. The median ICU stay was 3.5 days (IQR 2–6 days) and the median hospital stay was 25 days (IQR 14.5–70). Twenty patients (65%) required reintervention with either balloon dilation or re-stenting at a median age of 86.5 days (IQR 67.5–115.5). At 6-month follow-up, the median z-score for the left and right pulmonary artery diameters increased from − 1.1 (IQR − 1.71 to − 0.2) to − 0.7 (IQR − 1.27 to 0.1) (*p* 0.35) and − 1.31 (IQR − 1.8 to − 0.6) to − 0.09 (IQR − 1.2 to 0.33) (*p* 0.05), respectively. There was one procedure-related death and 4 interstage mortalities not directly related to the PDA stent.

**Conclusions:** PDA stenting in infants ≤ 2.5 kg is safe and effective, promoting pulmonary artery growth and improving oxygen saturations. Reintervention rates are relatively high.

## 104

### Usof the 7Q Micro Vascular Plug for Transcatheter Closure of Large Arterial Ducts in Infants Weighing Less Than 2.5 kg

Mohamed Al-Nasef^1^, Donnchadh O Sullivan^1^, Li Yen Ng^1^, Colm R Breatnach^1^, Kevin Walsh^1^, Paul Oslizlok^1^, Brian McCrossan^1^, Damien Kenny^1^, Shyam Sathanandam^2^

^1^Children Health Ireland, Dublin, Ireland. ^2^Heart center of Memphis, Memphis, USA

**Abstract**

**Background:** Transcatheter occlusion of the patent ductus arteriosus (PDA) in low and very low birth weight infants has become more commonplace, however, to date, there is only one FDA-approved PDA device for this group of patients, and it is not suitable for arterial ducts > 4 mm in diameter. We report a two-center experience using the 7Q Medtronic Micro Vascular Plug (MVP) to close large PDA’s in infants < 2.5 kg.

**Methods:** Retrospective review of departmental databases and medical charts to define patient cohort and collect demographic, procedural, and follow-up data.

**Results:** Twenty-two patients (12 male) with a median gestational age of 25.5 weeks (IQR 24–28) and median birth weight of 800 g (572–1075) underwent attempted PDA occlusion with 7Q MVP using exclusive transvenous approach. The median age and weight at time of PDA occlusion were 32 days (IQR 24–28) and 1100 g (IQR 960–1700), respectively. The median PDA length was 12 mm (IQR 11–12.65). The median aortic and pulmonary end of the PDA diameter were 5.1 mm (IQR 4.9–5.5) and 4.8 mm (IQR 4.6–5.3), respectively. Successful device occlusion was achieved in 95% of cases. There was one failed attempt as the arterial duct diameter was too large and therefore the device was removed. All except one patient had no residual shunt as demonstrated by the intraprocedural TTE. There were no procedural complications and no patients had increased Doppler velocities at either the left pulmonary artery or descending aorta post procedure.

**Conclusions:** The 7 Q MVP is safe and effective for providing transcatheter closure of large (> 4 mm) hemodynamically significant PDA’S in infants less than 2.5 kg. The lack of retention disk provides favorable device characteristics in avoiding impingement on surrounding structures.

